# Chiral DOTA chelators as an improved platform for biomedical imaging and therapy applications

**DOI:** 10.1038/s41467-018-03315-8

**Published:** 2018-02-27

**Authors:** Lixiong Dai, Chloe M. Jones, Wesley Ting Kwok Chan, Tiffany A. Pham, Xiaoxi Ling, Eric M. Gale, Nicholas J. Rotile, William Chi-Shing Tai, Carolyn J. Anderson, Peter Caravan, Ga-Lai Law

**Affiliations:** 1 Department of Applied Biology and Chemical Technology, The Hong Kong Polytechnic University, Hung Hom, Hong Kong SAR China; 2 The Athinoula A. Martinos Center for Biomedical Imaging, The Institute for Innovation in Imaging, Department of Radiology, Massachusetts General Hospital and Harvard Medical School, Charlestown, Massachusetts 02129 United States; 3 Department of Radiology, University of Pittsburgh, Pittsburgh, Pennsylvania 15213 United States; 4 Department of Medicine, University of Pittsburgh, Pittsburgh, 15261 Pennsylvania United States; 5 Departments of Pharmacology & Chemical Biology and Bioengineering, University of Pittsburgh, Pittsburgh, Pennsylvania 15213 United States

## Abstract

Despite established clinical utilisation, there is an increasing need for safer, more inert gadolinium-based contrast agents, and for chelators that react rapidly with radiometals. Here we report the syntheses of a series of chiral DOTA chelators and their corresponding metal complexes and reveal properties that transcend the parent DOTA compound. We incorporated symmetrical chiral substituents around the tetraaza ring, imparting enhanced rigidity to the DOTA cavity, enabling control over the range of stereoisomers of the lanthanide complexes. The Gd chiral DOTA complexes are shown to be orders of magnitude more inert to Gd release than [GdDOTA]^−^. These compounds also exhibit very-fast water exchange rates in an optimal range for high field imaging. Radiolabeling studies with (Cu-64/Lu-177) also demonstrate faster labelling properties. These chiral DOTA chelators are alternative general platforms for the development of stable, high relaxivity contrast agents, and for radiometal complexes used for imaging and/or therapy.

## Abstract (web summary)

MRI contrast agents containing the rare earth metal gadolinium are very effective, yet unstable and thus potentially hazardous. Here, the authors developed complexes between gadolinium and the scaffolding compound DOTA with increased stability, which also lend themselves to radiometal labelling.

## Introduction

Gadolinium-based contrast agents (GBCAs) are commonly used for diagnosis and monitoring of many diseases. Over 30 million doses of GBCAs are administered worldwide annually^1^. However, since 2006, it was recognised that free gadolinium ions are associated with a rare, but devastating toxicity called nephrogenic systemic fibrosis (NSF) in patients with renal impairment^2, 3^ leading to a contraindication of Gd-DTPA, Gd-DTPA-BMA and Gd-DTPA-BMEA in the patient population^4^. More recently, it has been found that gadolinium (Gd) can accumulate in the brains of patients, even those with normal renal function^5–7^. The European Medicines Agency (EMA) has recommended to restrict the clinical use of some acyclic (linear) Gd agents and suspend the authorisations of others in order to prevent any risks that could potentially be associated with Gd brain deposition.

GBCAs can be classified into macrocyclic and acyclic agents based on the nature of the chelating moiety. Association of Gd with NSF and brain deposition is higher for the acyclic Gd(III) chelates, which are more kinetically labile than the macrocyclic chelates^8^. The Food and Drug Administration also announced that additional text explaining that linear GBCAs carry a greater risk than macrocyclic agents will be added to the label. It is well-known that free Gd is toxic; the ionic radius of Gd^3+^ (108 pm) is close to that of Ca^2+^ (114 pm) causing Gd^3+^ to act as a potent antagonist of many types of voltage-gated calcium channels at very-low concentrations^9^. [GdDOTA]^−^, with the scaffold of 1,4,7,10-tetraazacyclododecane-1,4,7,10-tetraacetic acid (DOTA), has the highest thermodynamic and kinetic stability among the clinically used GBCAs^10^. However, several animal studies showed Gd deposition was also detected in brains following [GdDOTA]^−^ administration^11–13^, so the development of safer, more inert GBCAs is needed.

High relaxivity is also critical for MRI contrast agents. A key molecular factor for relaxivity is the water exchange rate at the Gd ion. [GdDOTA]^−^ and other GBCAs have water exchange rates that are too slow for optimal relaxivity^14^. In addition to providing a stable complex with respect to Gd release, the ideal chelator should also enable an optimal water exchange rate in the 10^8^–10^9 ^s^−1^ range^15–17^.

Beyond MR contrast agents, the DOTA chelator has shown great utility for binding radiometals like In-111 and Cu-64 for imaging and Lu-177 and Y-90 for therapy^18, 19^. Inertness of the complex with respect to metal release is a key feature here as well. In addition, mild, rapid labelling conditions are necessary properties of the chelator, especially when the chelator is conjugated to a thermally sensitive biomolecule.

Herein, we present the synthesis and structural properties of chiral DOTA system as a platform for safer and high relaxivity MR contrast agents, and for rapid radiolabelling with radiometals. Five chiral DOTAs with various chiral groups are synthesised. No substitution is performed on the acetate pendant arms in order to retain the natural chelating properties of the cavity formed from the eight coordinate points of N(4) and O(4). The chiral substituents on the opposite side of the metal ion can be used for targeting or conjugating with other molecules, while allowing the coordinated water molecules to exchange freely. We find that our complexes have optimal water exchange kinetics and high stability that transcends [GdDOTA]^−^ significantly, thereby providing an ideal platform to be merged with various macromolecules or nanoparticles through multilocus binding or conjugation to become contrast agents with high relaxivity for clinical diagnosis. Radiolabelling studies with Cu-64 and Lu-177 also demonstrate the faster labelling properties of chiral DOTA than DOTA, making them excellent candidates for use as radiometal chelators. Pharmacokinetics and biodistribution are readily tuned by the choice of chiral substituent.

## Results

### Synthesis of chiral DOTA complexes

**Fig. 1:**
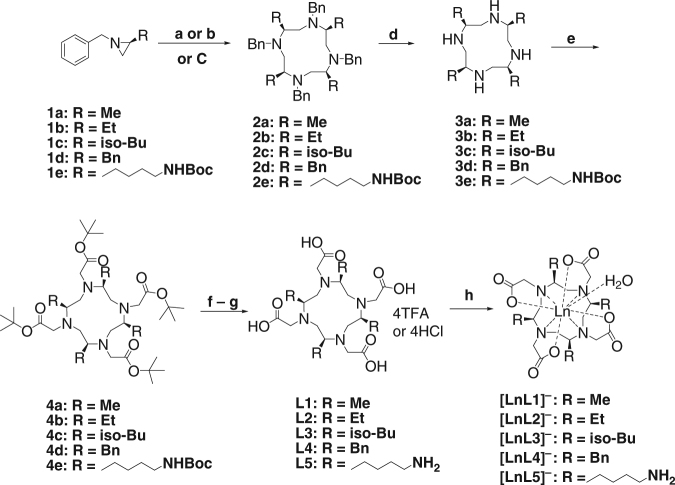
Synthetic procedures of chiral DOTA complexes. The chiral cyclens were synthesised from chiral aziridines followed by the incorporation of protected acetate arms and subsequent deprotections to give the final ligands. (**a**) TsOH/EtOH, RT (**1a**); (**b**) BF_3_·Et_2_O/Benzene, reflux (**1b** – **1d**), 24 h; (**c**) TsOH/ACN, RT (**1b**, **1e**); (**d**) Pd(OH)_2_/C, ammonium formate, trifluoroethanol, 50–75 °C, 16 h; (**e**) tert-butyl 2-bromoacetate, K_2_CO_3_, dry acetonitrile, 50–60 °C, 16 h; (**f**) TFA/DCM (1:1), 16 h; (**g**) 1 M HCl (**L2** – **L4**); (**h**) LnCl_3_·6H_2_O, pH 7.0, 80 °C, 12 h (Ln = Eu(III), Yb(III) and Gd(III)), note for clarity the counterions are not depicted

Five chiral DOTAs (**L1**–**L5**) with various chiral groups were synthesised (Fig. 1, Supplementary Fig. 1). We hypothesised that the chiral substituents would preorganize the chelator in a conformation favourable for metal ion binding, thereby increasing chelation kinetics. We further hypothesised that the chiral substituents would lock the conformation of the chelator making subsequent dechelation very slow. The synthesis of the twelve-membered ring of cyclen (1,4,7,10-tetraazacyclododecane) from an aziridine compound and the corresponding chiral substituted compounds have been studied for several decades^20^. However, literature examples of chiral cyclens are rare. The two backbones reported (with four methyl and four hydroxymethyls)^21, 22^ required very complicated synthetic procedures, which may be the main reason for their apparent unpopularity. The method of total synthesis of chiral cyclen with four 4-methoxybenzyl groups is even more daunting^23^. Interestingly, the better studied chiral cyclen compound (with four ethyl groups)^20, 24^ has no DOTA derivatives developed. In our work, we have improved and employed synthetic routes that simplifies the synthesis and purification of chiral cyclens.

The chiral cyclens (**2a** – **2e**) were synthesised from chiral aziridines (synthesised from natural amino acids), catalysed by either TsOH or Lewis acid boron trifluoride diethyl etherate^20–22^. We found that if TsOH is used, changing the solvent from ethanol to acetonitrile gave higher conversions and isolated yields.

**Fig. 2:**
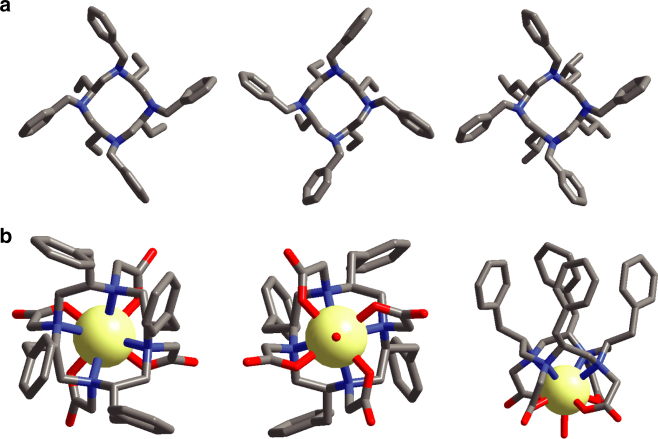
Crystal structures of ligands 2b, 2c and complex [GdL4]^−^. **a** Crystal structure of **2b** (*SSSS*-λλλλ), **2b** (*SSSS*-δδδδ) and **2c**. **b** Crystal structure of **[GdL4]**^**−**^ from top view, bottom view and side view. Atom labels: C (grey), N (blue), and O (red); H atoms omitted for clarity

The resulted twelve-membered ring compounds are 4-fold symmetric with the chiral substituents symmetrically arranged around the cyclen ring at one side of the plane and the benzyl groups located on the other side. This was confirmed by both NMR spectroscopy and X-ray crystallography (Fig. 2, Supplementary Table 1), with all four substituents in the *S* configuration, indicating that their absolute configurations did not change in the cyclization reactions. To further validate this, we crystallised both conformations of the enantiomeric **2b** from different phenyl ring solvents. **3a**–**3e** are also 4-fold symmetric, confirmed by ^1^H and ^13^C NMR spectroscopy as there is only one set of resonances while **4a**–**4e** are dissymmetric, as the four bulky tert-butyl acetate groups contributed to a crowded molecular environment with little freedom for rearrangement. Yet after removing the tert-butyl groups by deprotection, **L1**–**L5** were obtained in the form of either trifluoroacetic or hydrochloride salts and became 2-fold symmetric, with two sets of resonances with a ratio of ~1:1 observed. The respective lanthanide chiral DOTA complexes were synthesised by mixing the ligands and the metal ions and stirred at 80 °C for 12 h (pH 7.0). Single crystals of [**GdL4]**^**−**^ suitable for single X-ray diffraction were grown by slow evaporation of a solution mixture of DMF/H_2_O 1:10 (v/v). The geometry of **[GdL4]**^**−**^ was found to be TSAP/side (vide infra).

### Structural isomerism of chiral DOTA complexes

**Fig. 3:**
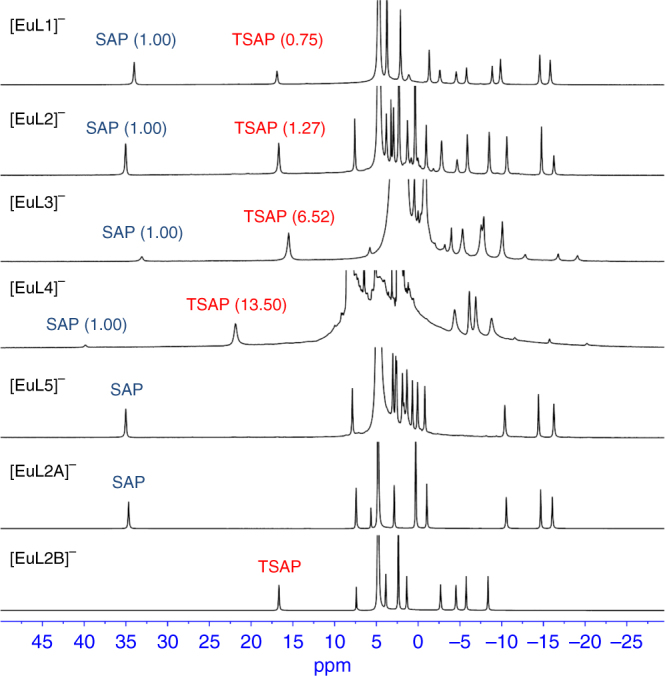
^1^H NMR spectra of Eu complexes. ^1^H NMR spectra of **[EuL1]**^**−**^ to **[EuL5]**^**−**^ with isolated SAP isomer **[EuL2A]**^**−**^ and TSAP isomer **[EuL2B]**^**−**^ of **[EuL2]**^**−**^ as isomer identification reference. The spectra gave ligand-dependent TSAP/SAP isomer ratios when Eu(III) is complexed with different ligands; attributed to the different substituents on the macrocyclic ring. The integrations of both isomer peaks are listed in parentheses with the integration of SAP isomer set as 1

In aqueous solution, the ratio of TSAP/SAP isomers of Eu(III) and Yb(III) complexes in DOTA-like systems can be easily identified by ^1^H NMR as two well-defined sets of six resonances are observed for the diastereotopic CH_2_CO and ring NCH_2_CH_2_N protons^25^. For Gd(III) complexes, although they cannot be analysed by NMR, their ratios of isomers are expected to be similar to the Eu(III) because the ionic radius of Gd(III) is very similar to that of Eu(III). No detectable change in the ratio of isomers was observed in the ^1^H NMR spectra of the complexes after storing for 6 months at room temperature. The ^1^H NMR spectra of the five Eu(III) chiral DOTA complexes are compared in Fig. 3.

It has been established that in solution, the square antiprismatic (SAP) structure is characterised by a shift to a higher frequency of the closest of the ring axial protons, while this resonance is less shifted for the twisted square antiprism (TSAP) isomer^26^. The isomer ratio of TSAP/SAP increased from 0.75 for **[EuL1]**^**−**^ to 1.27 for **[EuL2]**^**−**^ (in D_2_O, pD ~7.0). The trend is more pronounced with **[EuL3]**^**−**^ (6.52) and **[EuL4]**^**−**^ (13.50) as their TSAP isomers became more dominant (for these compounds, a 1:1 mixture of deuterated dimethyl sulfoxide and deuterium oxide was used to overcome solubility issues). This variation of isomer ratio accords with the general observations that increasing the steric demand at the bound metal ion favours the TSAP geometry^25^. However, only the SAP isomer was obtained for **[EuL5]**^**−**^, which may be related to the change in solvation environment around the butyl chain caused by the polar terminal amine group; more interestingly, we found these isomers could be easily isolated by reversed-phase HPLC (except for **[EuL1]**^**−**^).

**Fig. 4:**
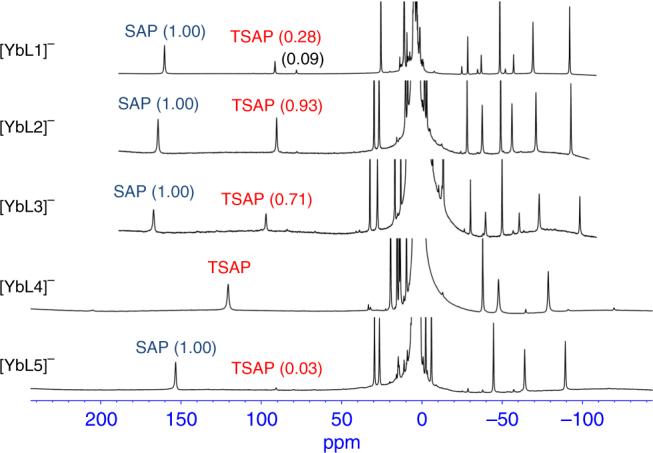
^1^H NMR spectra of Yb complexes. ^1^H NMR spectra of **[YbL1]**^**−**^ to **[YbL5]**^**−**^. The spectra gave ligand-dependent TSAP/SAP isomer ratios when Yb(III) is complexed with different ligands; attributed to the different substituents on the macrocyclic ring. The integrations of both isomer peaks are listed in parentheses with the integration of SAP isomer set as 1

To further demonstrate the variation tendency of TSAP/SAP isomer ratio between these five ligands, the ^1^H NMR spectra of the Yb(III) complexes were studied as well (Fig. 4). The ratio of TSAP/SAP isomers for **[YbL1]**^**−**^ (0.28) and **[YbL2]**^**−**^ (0.93) are relatively lower than the Eu(III) analogues; a minor isomer (0.09) that was described in **[YbL1]**^**−**^ was also observed^27^. The ratio of **[YbL3]**^**−**^ is similar to **[YbL2]**^**−**^ and those for **[YbL4]**^**−**^ and **[YbL5]**^**−**^ are consistent with their europium analogues. The difference in chemical shifts for complexes of **L4** is attributed to the aromatic nature of the benzyl substituent compared to the alkyl nature of the other compounds.

When the complexation conditions of **[EuL2]** and **[YbL2]** were used for **[GdL2]**^**−**^, the ratio of TSAP/SAP of **[GdL2]**^**−**^ was ~1:1 in the RP-HPLC trace, and the isolated yields for these two isomers were 46% (first peak, SAP) and 48% (second peak, TSAP), respectively. It is important to note that if the pH was adjusted to ~8.0 right after the addition of ligand, 70% of SAP and 25% of TSAP isomers would be obtained. This also emphasises that these two isomers formed are not interconvertible, and that the ratios of TSAP/SAP are determined by reaction kinetics. This makes it synthetically practical to control the isomerization by regulating the complexation conditions. Such phenomena were also observed with the similar NB-DOTA systems^28^. Another interesting discovery described in the Ln-NB-DOTA system is that the change in size/ionic radii of the Ln(III) metal ion does not influence the regioisomeric distribution^28^, and this also applies for our complexes (Supplementary Fig. 2).

### Stability studies of the chiral DOTA complexes

**[EuL2A]**^**−**^ and **[EuL2B]**^**−**^ showed extreme inertness upon heating at 90 °C (pD 7.0 and 6.0, respectively) for 3 days (Supplementary Fig. 3 & 4); no signs of racemization were observed in [**EuL2A]**^**−**^ either after heating for 3 days at pD 1.0 (Supplementary Fig. 5), nor did the presence of 1 equivalent of Zn^2+^ caused any instability (Supplementary Fig. 6).

**Fig. 5:**
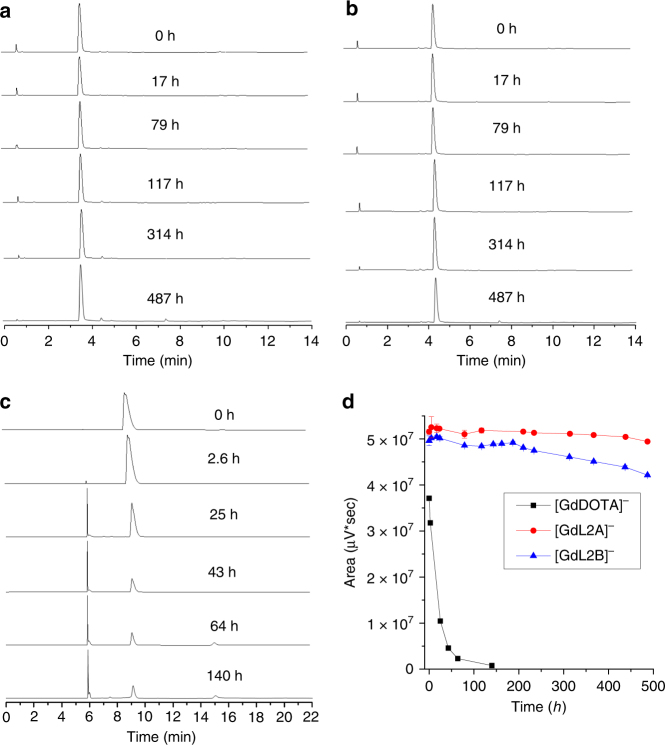
Stability of Gd complexes. **a**–**c** RP-HPLC traces of **[GdL2A]**^**−**^, **[GdL2B]**^**−**^ and [GdDOTA]^−^ in 1N HCl at room temperature over time. **d** Change in peak area integration over time. Quantitative analysis with RP-HPLC suggest **[GdL2A]**^**−**^ and **[GdL2B]**^**−**^ exhibit much higher stability than [GdDOTA]^−^ in acidic medium at room temperature. Error bars for **[GdL2A]**^**-**^ and **[GdL2B]**^**-**^ refer to the standard error of mean (S.E.M.), statistical data fitting analysis (n = 3; s.e.m.)

The kinetic stability of GBCAs, which reflects the dissociation of the Gd ions from its chelate is used to predict the stability in vivo^10^. The proton-assisted dissociation and direct attack of the endogenous ions are regarded as two main pathways that induced the dissociation of the chelates^10^. The inertness of our Gd(III) complexes was maintained up to 7 days after addition of 1000 equivalents of a competitive ligand, DTPA (Supplementary Fig. 7 & 8). Monitored by RP-HPLC^29^, **[GdL2A]**^**−**^ and **[GdL2B]**^−^ were also stable under both acidic and alkaline conditions, showing negligible decomplexation in 1N HNO_3_ (Supplementary Fig. 9 & 10) and 0.1N NaOH (Supplementary Fig. 11 & 12) over 10 days. The harshest condition tried was setting the complexes of **[GdL2A]**^**−**^, **[GdL2B]**^−^ and [GdDOTA]^−^ in 1N HCl (Fig. 5, Supplementary Figs. 13 - 15). **[GdL2A]**^**−**^ and **[GdL2B]**^−^ are much more stable than [GdDOTA]^−^; there was almost no decomplexation for **[GdL2A]**^**−**^ (SAP isomer), and only minimal decomplexation for **[GdL2B]**^**−**^ (TSAP isomer) after 487 h, while the half-life of [GdDOTA]^−^ was found to be shorter than 25 h. Their stability was maintained and no interconversion was observed with one equivalent of DOTA ligand in 1N HCl solutions of **[GdL2A]**^**−**^ and **[GdL2B]**^−^ (Supplementary Fig. 16). The complex **[GdL2A]**^**−**^ is also more stable than [GdDOTA]^−^ in the presence of 100 equivalents of zinc chloride at 50 °C. The half-life of [GdDOTA]^−^ was ~100 h under this condition, while no obvious decomplexation was observed for the complex of **[GdL2A]**^**−**^ during the same time (Supplementary Fig. 17 & 18).

**Fig. 6:**
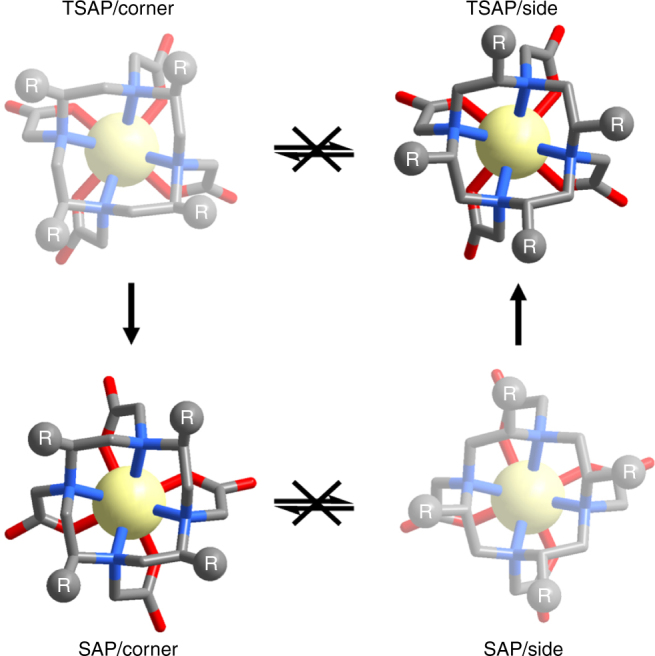
Restricted isomerism of [GdL3]^−^. Four stereoisomers are possible for [LnDOTA]^−^, but two of these structures are inaccessible with our system, they are represented by the washed out structures. SAP/corner and TSAP/side structures derived from the partial solved crystallographic data of **[GdL3]**^**−**^; R represents the chiral substituent. Atom labels: C (grey), N (blue) and O (red); H atoms omitted for clarity

So why are the chiral DOTA complexes more stable than [GdDOTA]^−^? In general, four different diastereomers are formed in the process of complexation with DOTA. Two pairs of enantiomers result in four stereoisomers that could interconvert in solution by ring-flip or rotation of the acetate pendant arms to give either monocapped SAP or monocapped TSAP geometries^30^. With four substituents, two of these isomeric structures are restricted and hence are not interconvertible (Fig. 6). The freezing of the mobility on the macrocyclic ring stabilised the chelate itself, while the steric hindrance prevents the interaction of the metal ion and the outer species, such as the transmetallation and acid catalysed decomplexation. In past studies with one benzyl group substituted on the macrocyclic ring, the resultant complexes showed relatively lower stability than [GdDOTA]^−^. The presence of corner and side regioisomers is illustrated in complexes of Ln-NB-DOTA^28^ and Ln-NB-DOTAM^31^. There are two pairs of interconvertible isomers for Ln-NB-DOTA^28^, however, due to the symmetry and sterics introduced into our systems, such regioisomers are eliminated (Fig. 6). This also indicates that the α-substitutions on the acetate arms (for [LnM4DOTMA]^−^)^32^ caused the structure to be too crowded, and lowered the stability to yield interconvertible isomers.

### Water exchange rates and relaxivity properties

It is evident that our chiral DOTAs can be selectively controlled to form TSAP/SAP isomers. This is very important because it is known that the TSAP/SAP isomers can exhibit very different water exchange kinetics of the inner-sphere coordinated water. Table 1 shows the summary of the mean residence time of the water co-ligand at 310K and enthalpy of activation of water exchange for Gd(III) complexes of **L1–L5** and their similar compounds.

**Table 1:** Coordination and fitting parameters ^17^O relaxation profiles

	[GdL1]^−^	[GdL2A]^−^	[GdL2B]^−^	[Gd(*R*)L2A]^−^	[Gd(*R*)L2B]^−^	[GdL5]^−^	[GdDOTA]^−^^43, 59^		Gd-DOTMA^43^		Gd-NB-DOTA^60^	Gd-NB-DOTMA(*S-SSSS*)^44^	Gd-NB-DOTMA(*S-RRR*)^44^
Coordination	Mix	SAP	TSAP	SAP	TSAP	SAP	SAP	TSAP	SAP	TSAP	Mix	SAP	TSAP
*τ*_M_^310K^ (ns)	13 ± 0.4	6.6 ± 0.4	1.1 ± 0.1	8.1 ± 0.4	2.9 ± 0.1	16 ± 0.8	141	27	156	26	111	77	9.4
Δ*H*^‡^ (kJ mol^-1^)	16 ± 1.4	43 ± 2.0	29 ±3 .2	33 ± 2.1	12 ± 1.7	18 ± 2.5	58.1 ± 2.5	41.9 ± 3.1	53.4 ± 2.9	40.3 ± 2.2	35.5	26.4	27.7

The mean residence time of the water co-ligand at 310 K (*τ*_M_^310K^) and enthalpy change of activation of water exchange (Δ*H*^‡^) for Gd(III) complexes. Each measurement was repeated three times and an average value is given. Error values refer to statistical data fitting analysis (n = 3; s.e.m.)

All our complexes have very-fast water exchange kinetics (fitting profiles were shown in Supplementary Fig. 19). The water residence time (*τ*_M_^310K^, inverse of exchange rate) of **[GdL1]**^**−**^, with one coordinated water molecule, was measured to be 13 ± 0.4 ns; it is a weighted average for all the coordination isomers present. The *τ*_M_^310K^ values of **[GdL2A]**^**−**^ and **[Gd(*R*)L2A]**^**−**^, diastereomer and enantiomer of **[GdL2]**^**−**^, respectively, were measured to be 6.6 ± 0.4 ns and 8.1 ± 0.4 ns; whereas the *τ*_M_^310K^ of **[GdL2B]**^**−**^ and **[Gd(*R*)L2B]**^**−**^ are 1.1 ± 0.1 ns and 2.9 ± 0.1 ns. **[GdL5]**^**−**^, exclusively in SAP geometry, has a *τ*_M_^310K^ of 16 ± 0.8 ns. These very-short water residence times are all close to the optimum value for the inner-sphere contribution in the MRI *T*_1_-shortening contrast agents as predicted by the Solomon–Bloembergen–Morgan (SBM) theory.

### Biodistribution studies and in vivo MRI

Most commercially available Gd(III)-based MRI contrast agents are excreted rapidly and exclusively through the renal pathway. One of the few exceptions is an acyclic compound, Gd-EOB-DTPA, which has a mixed clearance (50% renal, 50% biliary) and is used for imaging of the liver and biliary system^33^. Gd-BOPTA and MS-325 have predominantly renal elimination with <10% elimination through the biliary system. The blood half-life of MS-325 is unusually long owing to extensive serum albumin binding^34, 35^. We illustrated with our studies that there is a significant variation in the biodistribution profiles despite the small structural differences of the chiral substituents (Supplementary Fig. 20). Gd biodistribution in BALB/c mice was measured by ICP-MS at 5, 15, 30, 60, 180 and 360 min following injection. For the control/parent [GdDOTA]^−^, we observed rapid elimination from the body via the kidneys as expected^36^. Low levels of Gd(III) are present at 6 h post injection. On the other hand, with the tetramethyl-substituted **[GdL1]**^**−**^, near equal renal and hepatic clearance was observed. The tetraethyl derivative **[GdL2]**^**−**^ was mainly distributed in the liver but its retention time in the liver was quite short; with negligible amounts of Gd(III) detected after 60 min from the ICP-MS studies. The specific and unusual localisation of **[GdL2]**^**−**^ in the liver makes it a potential hepatobiliary-specific MRI contrast agent. Another interesting discovery was observed for **[GdL5]**^**−**^, where the localisation profile is mainly in the kidney and showed no tendency of clearance after 6 h; thus, we hypothesise that the four primary amino groups on the tetraaza ring may be recognised by transporters in the cortex of the kidney. Moreover, it is important and encouraging to note that minimal distribution in the brain was observed for all the compounds, implying our contrast agents are less toxic for clinical use.

**Fig. 7:**
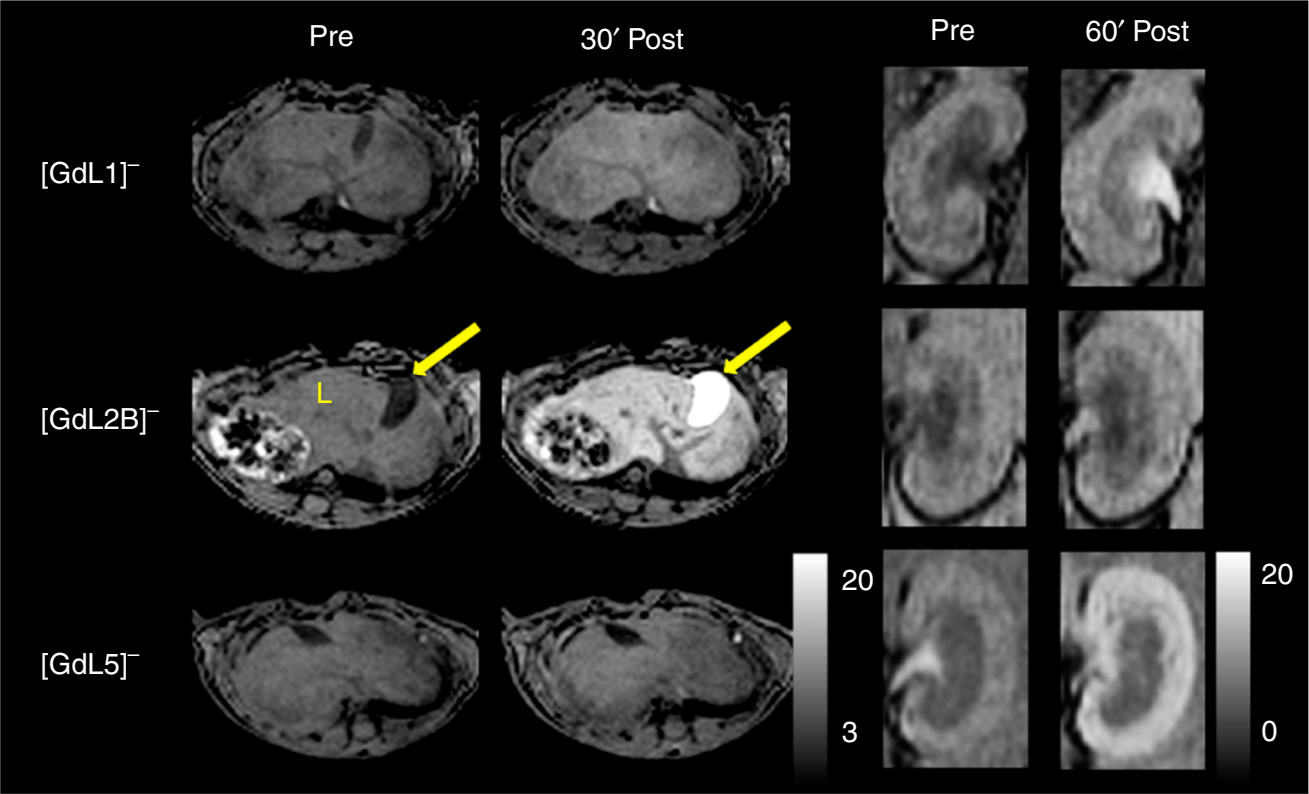
MR imaging in mice. Dynamic *T*_1_-weighted MR images at 4.7 tesla in C57/Bl6 mice before, after 0.1 mmol per kg injection of **[GdL1]**^**−**^, **[GdL2B]**^**−**^ and **[GdL5]**^**−**^. Axial images of the liver (L) and gall bladder (arrow) coronal images of the kidneys highlighting the differences in elimination route and organ enhancement among the complexes

Dynamic *T*_1_-weighted MR imaging at 4.7 tesla was performed with C57/Bl6 mice before, during, and after 0.1 mmol per kg injection of each compound (Fig. 7). For **[GdL1]**^**−**^, some enhancement of the liver and gall bladder and enhancement of the kidneys were observed as the contrast agent cleared, but 60 min post injection, the signal in the kidneys was similar to that at baseline. For **[GdL2B]**^**−**^, profound enhancement of the liver and gall bladder was observed, indicating efficient uptake by hepatocytes and elimination into the bile. Kidney enhancement was also observed at early time points, but by 60 min the kidney signal approached baseline. **[GdL2A]**^**−**^ showed a very similar behaviour to **[GdL2B]**^**−**^ but not **[GdL5]**^**−**^, where very little enhancement in the liver was observed.

In the kidneys, enhancement of the entire organ was observed immediately after injection. Over time, the signal in the medulla and renal pelvis approached baseline levels whereas the renal cortex remained strongly enhanced, indicative of retention of the compound **[GdL5]**^**−**^. Ex vivo biodistribution 2 h after injection confirmed the imaging findings with over 90 nmol Gd per g kidney in the animal receiving **[GdL5]**^**−**^, compared to ≤20 nmol Gd per g kidney with other contrast agents. The concentration of Gd(III) in the gall bladder was extremely high (854 and 1066 nmol per g) for **[GdL2A]**^**−**^ and **[GdL2B]**^**−**^, too (Supplementary Table 2).

### Radiometal labelling properties of L2

The labelling conditions were first evaluated with non radioactive ions. LCMS showed the labelling of **L2** with Cu(II) was fast at pH 6.0 at room temperature. The complexation of **L2** and Cu(II) salt completed nearly immediately after Cu(II) addition (Supplementary Fig. 21). Next our ligand was tested with cold Lu(III) as ^177^Lu(III) is a good radioisotope in radiotherapy. The labelling of **L2** with Lu(III) occurred at pH 7.0 at room temperature with the metal to ligand ratio 1:1, while higher temperature is needed to get higher conversion, which was monitored by LCMS (Supplementary Fig. 22 & 23).

Similar to cold labelling, instantaneous formation of ^**64**^**CuL2** with the radioactive isotope ^64^Cu was observed using radio-HPLC (pH 6.1, room temperature) with near quantitative radiochemical yield. Radiochemical yield of 100 % was achieved after brief heating at 40 °C for 30 min (Supplementary Fig. 24, Supplementary Table 3). In comparison, the radiochemical yield of ^64^CuDOTA under the same conditions was 89% upon mixing at room temperature and 95% after heating at 40 °C for 30 min (Supplementary Fig. 25).

**Fig. 8:**
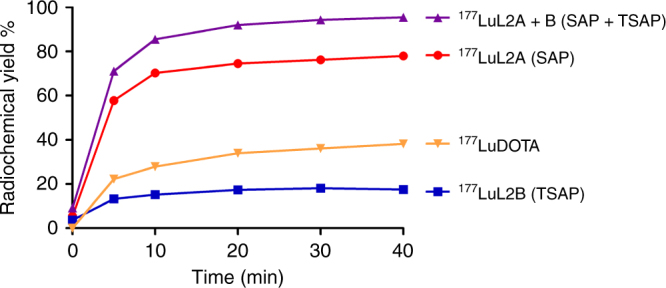
Radiolabeling yields of ^177^Lu complexes. A graph of the radiolabeling yields of **L2** and DOTA with ^177^Lu(III) against time at 95 °C in pH 5.0 buffer, showing that **L2** is a better chelator for ^177^Lu(III)

In another radiolabeling study with ^177^Lu(III), it was found that at least 80 °C was required to obtain **[**^**177**^**LuL2]**^**−**^. The radiochemical yield of **[**^**177**^**LuL2]**^**−**^ was near quantitative after 20–40 min at 95 °C (pH 5.0) with the highest radiochemical yield over 95% (Fig. 8, Supplementary Fig. 26, Supplementary Table 4). Compared to the formation of **[**^**177**^**LuL2]**^**−**^ at the same conditions, [^177^LuDOTA]^−^ formation was slower with a radiochemical yield of 38% when heated at 95 °C (Fig. 8, Supplementary Fig. 27). Last but not least, **[**^**177**^**LuL2]**^**−**^ can also be obtained in pH 7.0 with an excellent radiochemical yield of 97% (95 °C, 20 min), which is not achievable at such mild pH conditions with DOTA. All of these results indicate that **L2** is a much better ligand than DOTA for complexation of these two radioisotopes.

The stability of the complex is also a key parameter that needs to be considered for a radiometal chelate^19^. The stability of CuDOTA and **CuL2** was compared by UV-Visible spectra. In 1N HCl, CuDOTA showed rapid decomplexation, and the half-life of the complex at these conditions was similar to the [GdDOTA]^−^ (<24 h) (Supplementary Fig. 28). The chiral version of **CuL2** under the same conditions was much more stable. Because the ionic radii of Lu(III) is very close to Yb(III), so we used the Yb(III) as a surrogate to study the stability. Monitored by ^1^H NMR, the complex of [YbDOTA]^−^ in 1N DCl also decomplexed very fast, but no obvious decomplexation was observed for the complex of **[YbL2]**^**−**^ (Supplementary Fig. 29). Additionally, we examined in vitro serum stability of ^**64**^**CuL2** at 37 °C. The radiochemical purity of ^**64**^**CuL2** decreased by less than 0.3 and 6.3% after 1 h and 24 h of incubation, respectively.

## Discussion

The conformation of the Gd(III)-based complexes plays a significant role in the water exchange rate *k*_ex_ (*k*_ex_ = 1/*τ*_M_)^37^. It has been shown that the *k*_ex_ in the TSAP configuration is 10–100 times faster than SAP, with the former useful as *T*_1_-shortening contrast agents, and the latter desirable as paramagnetic chemical exchange saturation contrast agents^38, 39^. Chiral DOTAs directly influenced the conformation of their lanthanide complexes. The ratio of formation of TSAP and the SAP isomers of these chiral DOTA lanthanide complexes can be controlled by the choice of functional groups used; as the bulkiness of these substituents increases, the ratio of TSAP/SAP also increases; over 93% of TSAP isomer of **[LnL4]**^**−**^ was obtained when benzyl groups were used. However, when four aminobutyl groups, which are more flexible and bulky, were introduced in **[LnL5]**^**−**^, ~100% of SAP geometry was formed. High temperature ^1^H NMR experiments confirmed that the ratios of isomers are stable and displayed no obvious change over various temperatures. Studies with different Ln(III) ions also revealed no change in the isomer ratios, dismissing the influence of metal ion size. Besides MRI, the control of the stereoisomerism of the complexes is also very important for some other applications: The stereoisomeric purity of chiral luminescent lanthanide complexes are crucial for circularly polarised luminescence (CPL) applications^30, 40;^ Such rigid paramagnetic complexes are also ideal pseudocontact shift tags for proteins for NMR studies in solution and even in cells^41, 42^.

Gd-DOTMA and Gd-NB-DOTMA (*S-RRRR*), have relatively fast water exchange rates in the TSAP geometry^43, 44^. However, the study of Gd-NB-DOTMA found that a faster water exchange rate gave a less efficiency than the slower one as the hydration state (*q r*^−6^) is inversely proportional^45^. However, we found no difference in relaxivities between the SAP and TSAP isomers in our study. From the structures of the complexes, we noted that the substitution on the α-position of acetate arms of the DOTA complex may change the cavity of the chelator more than the substitutions on the tetraaza ring; this is because the hydrophobic bulky substituents near the coordinated water might affect the interaction of the inner and outer sphere water molecules. While for our chiral DOTA complexes, the coordination of the water molecule in the first sphere of the metal ions are retained like their parent [GdDOTA]^−^ complex, for the complexes with very-short water residence time (s), like **[GdL2B]**^**−**^, the water residence time is optimal for designing contrast agents with high relaxivity at high magnetic fields (≥3 tesla)^46–48^.

The *r*_1_ values of **[GdL1]**^**−**^, **[GdL2]**^**−**^ (**[GdL2A]**^**−**^, **[GdL2B]**^**−**^, **[Gd(*R*)L2A]**^**−**^, **[Gd(*R*)L2B]**^**−**^) and **[GdL5]**^**−**^ are largely invariant in water at 1.4 T, 37 °C. Given the similar molecular weights and similar chemical structures, one expects similar rotational correlation times for these complexes. The similar relaxivities suggest that the hydration number for these compounds is the same and *q* = 1 by analogy to the Gd-DOTA-type complexes (Table 2). The molecular weights of all the complexes are greater than [GdDOTA]^−^, which results in modestly higher relaxivities. We also measured relaxivities in 4.5% (w/v) human serum albumin (HSA) solution and in human plasma. The relaxivities are slightly elevated in these media but not more than [GdDOTA]^−^, suggesting little to no plasma protein binding. The favourable properties of these Gd complexes, such as fast and tunable water exchange kinetics, kinetic inertness with respect to Gd release, and ease of derivatization indicate that the chiral DOTA technology will serve as a very-useful platform in the design of high relaxivity and targeted MRI contrast agents^49–51^. For example, our chiral cyclen platform enables four inert substituents on the other side of the tetraaza plane to be potentially conjugated with four binding moieties to minimise the rotation of the whole molecule, therefore enabling maximum relaxivity enhancement. Such a strategy to achieve multilocus binding has been proven successful in the literature^52–54^.

**Table 2:** Relaxivities of Gd complexes

	Water (1.4T)		4.5% w/v HSA (1.4T)		Human Plasma (1.4T)	
Sample	*r*_1_	*r*_2_	*r*_1_	*r*_2_	*r*_1_	*r*_2_
**[GdL1]** ^**-**^	4.1	5.1	4.5	5.3	4.6	5.5
**[GdL2A]** ^**-**^	4.2	5.5	4.7	5.5	4.7	5.6
**[GdL2B]** ^**-**^	3.9	4.9	4.8	6.0	4.9	6.1
**[Gd(** ***R*** **)L2A]** ^**-**^	3.9	5.0	4.5	4.9	4.6	4.8
**[Gd(** ***R*** **)L2B]** ^**-**^	3.9	4.9	4.8	6.1	4.9	6.1
**[GdL5]** ^**-**^	4.1	5.9	5.1	6.3	5.1	6.3
[GdDOTA]^-^	3.2	3.2	4.1	4.8	4.0	4.0

Summary of relaxivities of **[GdL1]**^**-**^, **[GdL2A]**^**-**^, **[GdL2B]**^**-**^, **[Gd(*R*)L2A]**^**-**^, **[Gd(*R***)**L2A]**^**-**^, **[GdL5]**^**-**^ and [GdDOTA]^-^ in water, HSA and human plasma.

Our chiral modifications of the ring have forced the four acetate groups to form a natural cavity, which enables better chelation. This is in contrast to the normal DOTA chelate in which, with only four acetate groups, the pendant arms form a more disordered structure, thus having a lower entropy in the process of sequestering the metal ions; hence, we predict that leads to a much more rapid complexation rate. This resonates with our hypothesis that the formation of natural cavity in our chiral DOTA ligands from having four extra substituents on the same side simplifies the structural preorganisation step in the complexation leading towards fast complexation.

In summary, we demonstrated the synthesis of a series of chiral DOTA chelators with four substituents symmetrically arranged around the cyclen ring. Our chiral DOTA Gd(III) complexes display faster water exchange kinetics (*τ*_M_ 1.1–16 ns at 310K) than [GdDOTA]^−^ and are stable at harsh temperature and extreme pH environments. To the best of our knowledge, only one lanthanide complex has been published with comparable kinetic inertness thus far^55^.These complexes also have unique biodistribution profiles for organ-specific applications, such as the hepatobiliary-specific **[GdL2]**^**−**^ and show near-zero distribution in mice brains. The work shows that our chiral DOTA chelators, in terms of structural rigidity, inertness, water exchange kinetics and fast radiolabelling properties have clearly transcended the DOTA ligand, and show promise as an ideal platform for the development of safer and more efficient agents for potential applications as protein tags for NMR and electron paramagnetic resonance (EPR) studies, highly efficient MRI contrast agents and radiometal chelators for PET or SPECT imaging.

## Methods

### General materials and methods

Unless otherwise noted, all chemicals were of reagent-grade and were purchased from Sigma-Aldrich or Acros Organics and used without further purification, (2 *S*)-1-Benzyl-2-ethylaziridine, (*S*)-1-benzyl-2-isobutylaziridine and (*S*)-1,2-dibenzylaziridine were ordered from Beijing Peridachem Co., Ltd. ^1^H and ^13^C NMR spectra were recorded on a Bruker Ultrashield 400 Plus NMR spectrometer (at 400 MHz and 100 MHz, respectively) or a Bruker Ultrashield 600 Plus NMR spectrometer (at 600 MHz and 150 MHz, respectively). Unless otherwise specified, chemical shifts *δ* were expressed in parts per million (ppm) based on the residual solvent signal in DMSO-d^6^ (*δ* 2.50 ppm), D_2_O (*δ* 4.79 ppm), CD_3_OD (*δ* 3.31 ppm) or tetramethylsilane in CDCl_3_ (*δ* 0.00 ppm), and coupling constants *J* are given in Hz. Coupling patterns are abbreviated as s (singlet), d (doublet), t (triplet), q (quartet), dd (doublets of double), ddd (doublets of doublets of doublet), dt (doublets of triple), dtd (doublets of triplets of doublet), dq (doublets of quartet), and m (multiplet). Liquid chromatography-mass spectrometry was performed using an Agilent 1100 series apparatus with an LC/MSD trap and Daly conversion dynode detector with UV detection at 220, 254, and 280 nm. The method used on this system is as follows: Restek ultraaqueous C18 column (100 × 4.6 mm); eluent A: 10 mM ammonium acetate in water, B: 90% acetonitrile/10% 10 mM ammonium acetate in water; gradient: 5% B was held for 2 min before increasing the fraction of B to 50% over 10 min. The column was washed with 95% B for 2 min and then ramped to 5% B. The system was re-equilibrated at 5% B for 2 min. Reverse-phase semi-preparative purification was performed on either of these two systems. System A: Rainin Dynamax HPLC system with UV detection from 220 to 280 nm using a Restek Ultraaqueous C18 Column (250 × 10 mm). The mobile phase A was water with 10 mM ammonium acetate added; mobile phase B was 90% acetonitrile/10% water with 10 mM ammonium acetate added. The method used for purification is as follows: starting from 95% A/5% B, the fraction of B increased to 50% over 23 min. The column was washed with 95% B for 2 min and then ramped to 5% B. The system was re-equilibrated at 5% B for 3 min. System B: Waters HPLC system with UV detection from 210 to 280 nm using a Waters C18 Column (250 × 19 mm). The mobile phase A was water with 10 mM ammonium formate added; mobile phase B was 90% acetonitrile/10% water with 10 mM ammonium formate added; The method used for purification is as follows: starting from 95% A/5% B, the fraction of B increased to 50% over 23 min. The column was washed with 95% B for 2 min and then ramped to 5% B. The system was re-equilibrated at 5% B for 3 min. Ultraperformance liquid chromatography-high resolution mass spectrometry was performed using an Agilent 1260 Infinity Series apparatus with Agilent 6540 UHD Accurate-Mass Q-TOF LC/MS detection in the range of 100–3000. The method used on this system is as follows: Agilent Eclipse Plus C18 RRHD 1.8 µm column (50 × 2.1 mm), eluent A: water with 0.1% formic acid, eluent B: acetonitrile. Radio-HPLC was performed using an Agilent 1260 Infinity Series apparatus with Phenomenex Luna 5 u C18(2) 100 Å (150 × 4.6 mm); eluent A: water with 0.1% trifluoroacetic acid, eluent B: acetonitrile with 0.1% trifluoroacetic acid.

### Syntheses

All the compounds were fully characterized. Experiment details and characterisations are given in Supplementary Methods (NMR spectra were shown in Supplementary Figs. 30–82, high resolution ESI-MS of complexes were shown in Supplementary Figs. 83–92).

### Relaxivity and water exchange rate measurements

Relaxivity measurements were performed on a Bruker mq60 Minispec at 1.41T and 37 °C. Longitudinal (*T*_1_) relaxation times were measured using an inversion recovery experiment with 10 inversion times of duration ranging between 0.05 × *T*_1_ and 5 × *T*_1_. Transverse (*T*_2_) relaxation times were measured using a CPMG pulse sequence. Relaxivity (*r*_1_, *r*_2_) was determined from the slope of a plot of 1/*T*_1_,_2_ vs [Gd] for at least 4 concentrations of Gd(III) (data shown in Supplementary Figs. 93–110). The *T*_2_ relaxation times of ^17^O at 11.7T were estimated from the full-width at half-height of the H_2_^17^O resonance at temperatures ranging from 278 to 368K. Reduced relaxation rates (1/*T*_2r_) were calculated by taking the difference of 1/*T*_2_ measured in the presence and absence of Gd(III) and dividing by the mole fraction of coordinated water molecules, assuming *q* = 1. This data was plotted vs reciprocal temperature and fit to a 4-parameter model dependent on water exchange and electronic relaxation as described previously^56^. However, at the high magnetic field used, and because of the very-fast water exchange rates observed, there was negligible contribution of *T*_1e_, and the data were refit to a two-parameter model using the water exchange rate at 310K (*τ*_M_) and enthalpy of activation for water exchange (Δ*H*^‡^) as adjustable parameters. The Gd-O hyperfine coupling constant, A/ħ, was assumed to be -3.8 × 10^6 ^rad s^−1^ ^57, 58^. Samples were prepared in neat H_2_O adjusted and enriched with a small amount of H_2_^17^O.

### Gd quantification

Gd concentrations were determined using an Agilent 8800-QQQ ICP-MS system. All samples were diluted with 0.1% Triton X-100 in 5% nitric acid. Lutetium internal standard was steadily introduced to the ICP-MS from an external source during the analysis. A linear calibration curve for each metal ranging from 0.1 ppb to 200 ppb was generated daily for the quantification.

### Protein binding

Measurements were performed on a series of solutions ranging between 15-400 µM Gd complex in either 4.5% wt/v (HSA) or human blood plasma. A volume of 500 µL of each solution was placed within a Millipore Ultra Free MC 30 kDa cutoff filtration vessel and 20 µL of the solution was forced through the filter by centrifugation. Gd content in each unfiltered solution and filtrate were quantified by ICP-MS. The percentage of each complex bound to albumin was determined from the difference in Gd concentrations between unfiltered solution and filtrate.

###  In Vivo MR imaging

Imaging was performed at 4.7T using a small-bore animal scanner (Bruker Biospec). Pilot dynamic MRI studies were performed with **[GdL1]**^−^, **[GdL2A]**^−^, **[GdL2B]**^**−**^ and **[GdL5]**^**−**^; n = 1 per compound. Six to eight weeks male C57/Bl6 mice were anaesthetised with isoflurane (1%–2%) and placed in a specially designed cradle with body temperature maintained at 37 °C. The tail vein was catherised for intravenous delivery of the contrast agent while the animal was positioned in the scanner. A pneumatic pillow was placed on the mouse to enable respiratory monitoring (SA Instruments), and the animal’s body temperature was maintained with warm air. The imaging protocol utilised a three-dimensional fast low angle shot (3D FLASH) with a 12 °C flip angle, 9.00 ms repetition rate, 2.39 ms echo time, 4.8 cm × 2.4 cm × 2.4 cm field of view with a 0.3 mm isotropic resolution. Images were acquired with either 2 or 8 averages giving an acquisition time of 2 min or 8 min, respectively. Baseline images were acquired with both 2 and 8 averages, then the Gd complex (0.1 mmol per kg) was injected intravenously and the sequence with 2 averages was repeated for a period of 10 min to monitor blood clearance. At 10, 30, 45, 60, 75 and 90 min after probe injection, the sequence with 8 averages was repeated to monitor the clearance of contrast from the liver and kidneys. After imaging was performed, the mouse was removed from the scanner and euthanized at 2 h post injection and the tissues were harvested for Gd quantification by ICP-MS.

### Gd complexes biodistribution studies

Complexes **[GdL1]**^**−**^, **[GdL2]**^−^ and **[GdL5]**^**−**^ were prepared by dissolving in saline solution, while [GdDOTA]^−^ (Dotarem) was diluted in saline solution. Eight-week-old male BALB/c mice (purchased from BioLASCO, Taiwan) each weighing approximately 25 g were administered with a single dose of 0.025 mmol/kg compound (**[GdL1]**^**−**^, **[GdL2]**^**−**^, [GdDOTA]^−^ and **[GdL5]**^**−**^; 3 mice per compound per time point) in a 100 μl volume via lateral tail vein injection. The mice were then euthanized, and their blood collected by cardiac puncture at each time point (5, 15, 30, 60, 180 or 360 min post injection). A control group of 4 mice was treated with saline and killed at time point 0. The liver, lung, heart, kidneys, spleen and brain were removed from the animals and weighed. Gd content in tissue and blood samples was quantified via ICP-MS analysis. All experiments were performed in accordance with the NIH Guide for the Care and Use of Laboratory Animals and approved by the Institutional Animal Care and Use Committee of Massachusetts General Hospital or by the Department of Health of the Hong Kong Government and the Animal Subjects Ethics Sub-Committee of Hong Kong Polytechnic University.

### ^64^Cu labelling

Vials containing 5 nmol of **L2** or **DOTA** (Macrocyclics, Plano, TX) [2.5 μL of 2 mM solutions in water], and 50 μL of 0.5 M NH_4_OAc buffer (pH 6.05) were mixed with 1.5 µL of ^64^CuCl_2_ solution (1.0 mCi, from Washington University). The reaction solutions were mixed and briefly centrifuged. A small aliquot was taken immediately and diluted with 50 µL of 0.5 M NH_4_OAc (pH 6.05) and analysed by radio-HPLC. The mixtures were then heated to 40 °C for 20 min. A small aliquot was taken and analysed by radio-HPLC to quantify radiochemical yields.

### ^177^Lu labelling

Vials containing 4 nmol of **L2** (2 μL of 2 mM solution in water), and 100 μL of 0.5 M NH_4_OAc buffer (pH range from 4.0 to 7.0) were mixed with 0.2 μL of ^177^LuCl_3_ (in 0.1 M HCl; MURR, Columbia, MO). The reaction mixtures were mixed, briefly centrifuged and left to react at specific temperatures. A small aliquot of the crude labelling mixtures was taken at each time point and analysed by radio-HPLC to quantify radiochemical yields.

### Data availability

The X-ray crystallographic data has been deposited at the Cambridge Crystallographic Data Centre with deposition number (CCDC 1517731), (CCDC 1517730), (CCDC 1517732), (CCDC 1517729), (CCDC 1445318) and (CCDC 1497014). This data can be obtained free of charge via www.ccdc.cam.ac.uk/conts/retrieving. html. All other data supporting the finding in this study are available within the article and its Supplementary Information, as well as from the authors upon reasonable request.

## Electronic supplementary material

Supplementary Information
