# Supplementary material for: Chiral DOTA chelators as an improved platform for biomedical imaging and therapy applications
Source: Nat Commun. 2018 Feb 27;9:857. doi: 10.1038/s41467-018-03315-8 (PMC5829242; doi:10.1038/s41467-018-03315-8)
Supplement: Supplementary file 1 — Supplementary Information [file 41467_2018_3315_MOESM1_ESM.pdf]

Supplementary Information for “Chiral DOTA Chelators as an Improved Platform for Biomedical Imaging and Therapy Applications”

Lixiong Dai, Chloe M. Jones, Wesley Ting Kwok Chan, Tiffany A. Pham, Xiaoxi Ling, Eric M. Gale, Nicholas J. Rotile, William Chi-Shing Tai, Carolyn J. Anderson, Peter Caravan, and Ga-Lai Law

Supplementary Figures

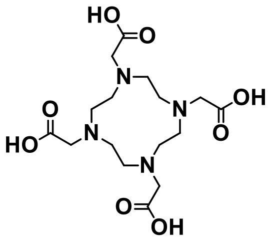

DOTA

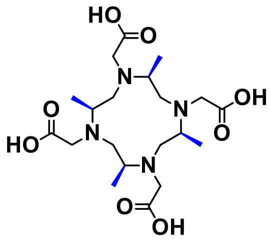

L1

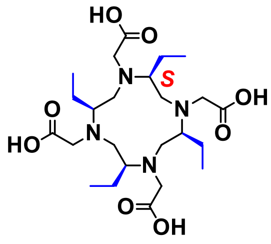

L2

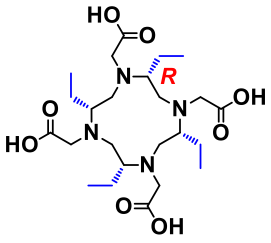

(R)L2

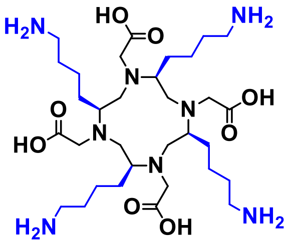

L5

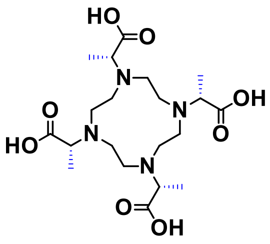

DOTMA

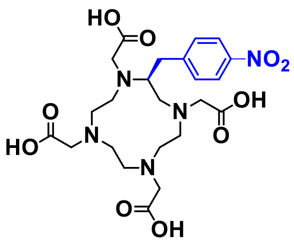

NB-DOTA

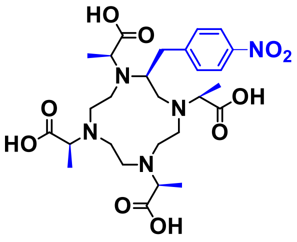

NB-DOTMA *(S-SSSS)*

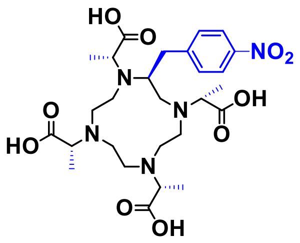

NB-DOTMA *(S-RRRR)*

Supplementary Figure 1 | Ligand structures compared in main text of Table 1. The chiral groups were highlighted with blue colour and the configurations of **L2** and **(*R*)L2** were labelled with red letters.

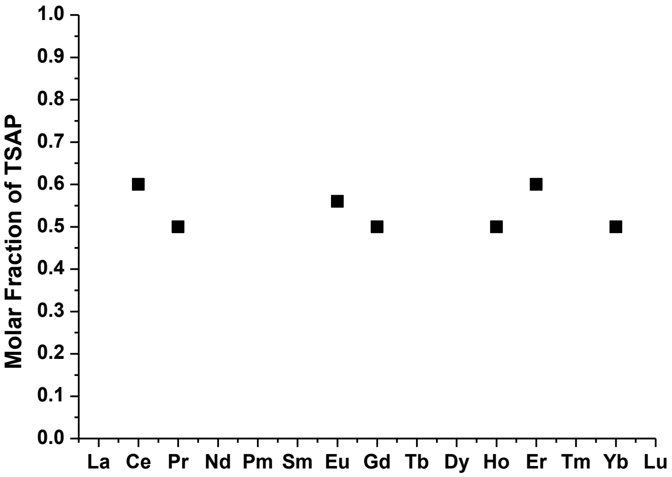

Supplementary Figure 2 | Mole fractions of the TSAP isomers of [LnL2]$^-$. With the same complexation condition of **[EuL2]**$^-$.

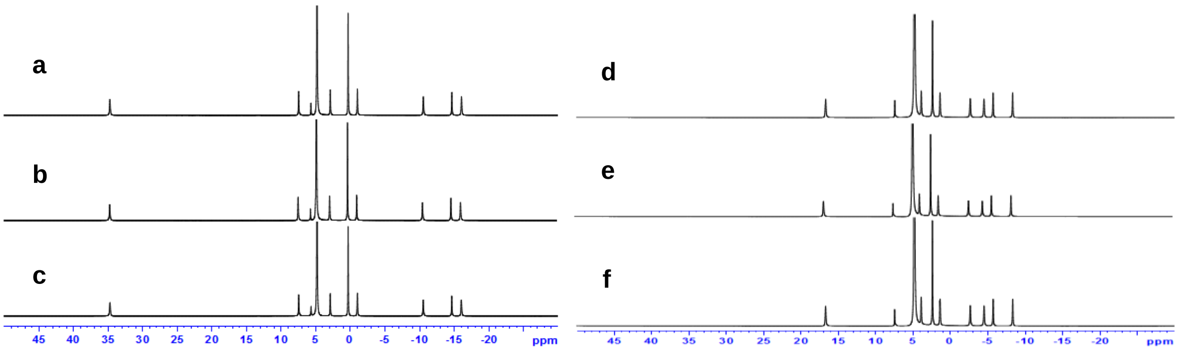

Supplementary Figure 3 | The ^{1}H NMR spectra of [EuL2A]$^-$ and [EuL2B]$^-$ after heating. a ^{1}H NMR spectrum of **[EuL2A]**$^-$ before heating; **b** ^{1}H NMR spectrum of **[EuL2A]**$^-$ after heating at 90 °C for 24 h; **c** ^{1}H NMR spectrum of **[EuL2A]**$^-$ after heating at 90 °C for 96 h. **d** ^{1}H NMR spectrum of **[EuL2B]**$^-$ before heating; **e** ^{1}H NMR spectrum of **[EuL2B]**$^-$ after heating at 90 °C for 24 h; **f** ^{1}H NMR spectrum of **[EuL2B]**$^-$ after heating at 90 °C for 96 h. The samples were cooled to ambient temperature prior to acquisition of NMR data.

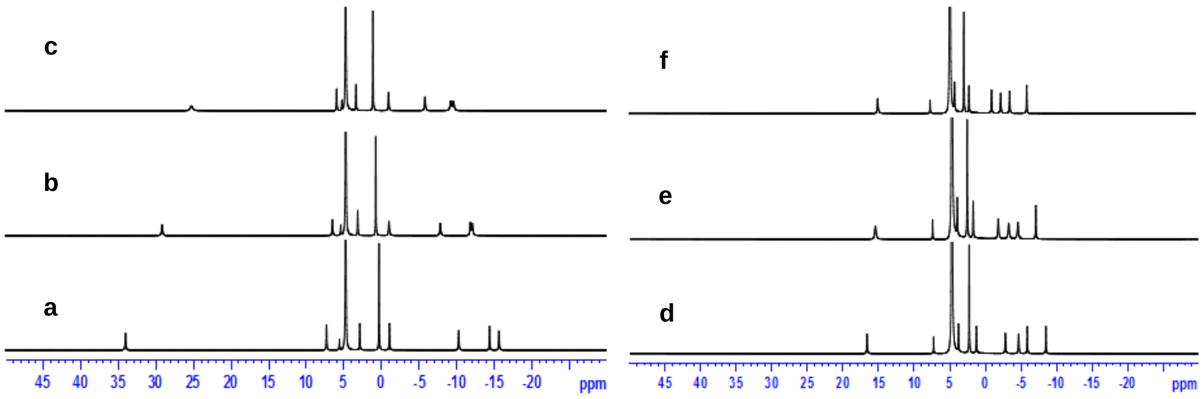

Supplementary Figure 4 | ^{1}H NMR spectra variation upon temperature rise. a ^{1}H NMR spectrum of **[EuL2A]**$^{-}$ at room temperature; **b** ^{1}H NMR spectrum of **[EuL2A]**$^{-}$ at 60 °C; **c** ^{1}H NMR spectrum of **[EuL2A]**$^{-}$ at 90 °C; **d** ^{1}H NMR spectrum of **[EuL2B]**$^{-}$ at room temperature; **e** ^{1}H NMR spectrum of **[EuL2B]**$^{-}$ at 60 °C; **f** ^{1}H NMR spectrum of **[EuL2B]**$^{-}$ at 90 °C.

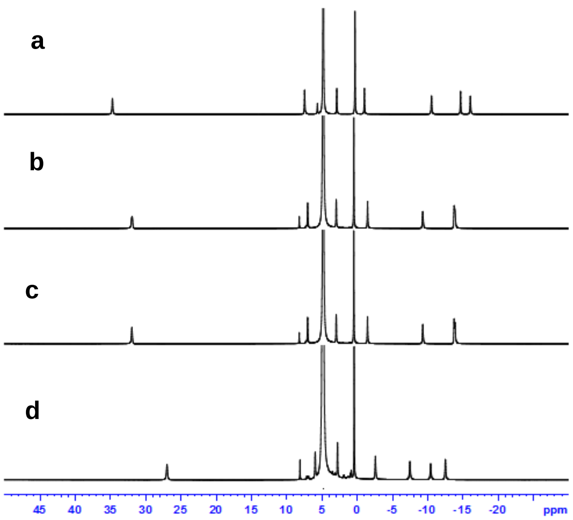

Supplementary Figure 5 | ^{1}H NMR spectra variation upon pD values changing for [EuL2A]$^{-}$. a ^{1}H NMR spectrum of **[EuL2A]**$^{-}$ in D_2O at pD 4.8; **b** ^{1}H NMR spectrum of **[EuL2A]**$^{-}$ in D_2O at pD 1.0; **c** ^{1}H NMR spectrum of **[EuL2A]**$^{-}$ in D_2O at pD 1.0, after heating at 85 °C for 15 h; **d** ^{1}H NMR spectrum of **[EuL2A]**$^{-}$ in D_2O at pD 0.2, after heating at 85 °C for 22 h. (The samples were cooled to ambient temperature prior to acquisition of NMR data).

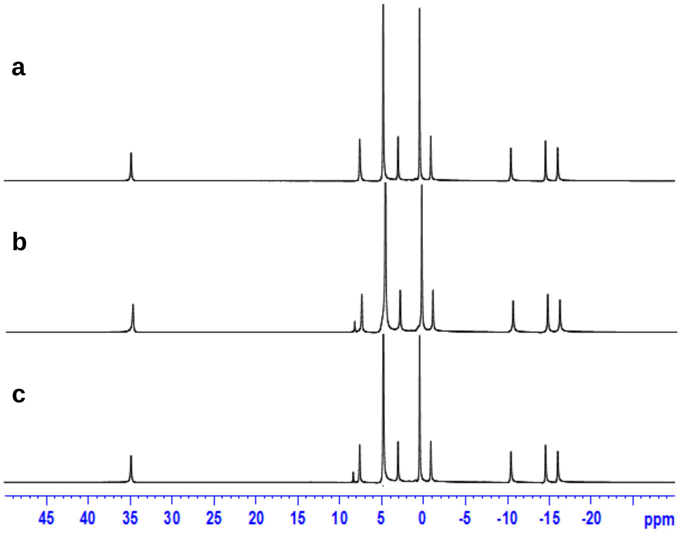

Supplementary Figure 6 | ^{1}H NMR spectra variation upon adding Zn^{2+} for [EuL2A]$^-$. a ^{1}H NMR spectrum of **[EuL2A]$^-$** in D_2O; **b** ^{1}H NMR spectrum of **[EuL2A]$^-$** after adding 1 eq. of $Zn(OTf)_2$ and 1 eq. of Na_2HPO_4; **c** ^{1}H NMR spectrum of **[EuL2A]$^-$** in the presence of Zn^{2+} and shaking for 44 h at 37 °C. It is known that free Eu^{3+} displaced by Zn^{2+} would precipitate from the solution as $EuPO_4$.[1]

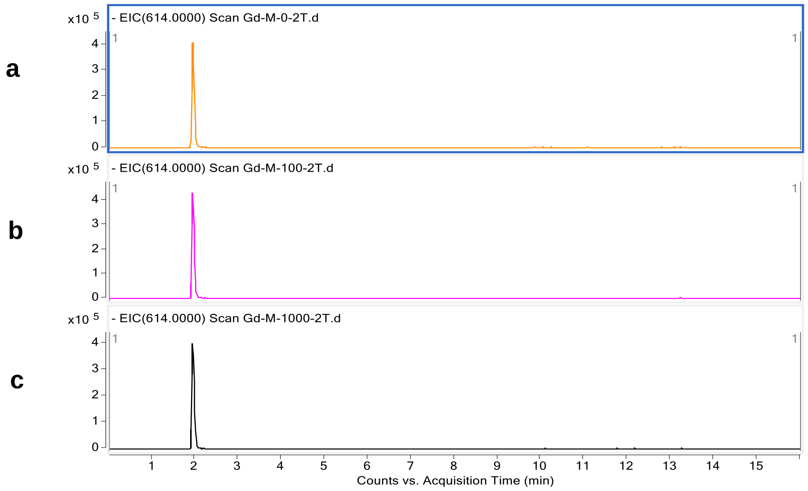

Supplementary Figure 7 | Competitive batch titration of [GdL1]$^-$. a Extracted UPLC-HRMS spectrum of **[GdL1]$^-$** in absence of DPTA; **b** Extracted UPLC-HRMS spectrum of **[GdL1]$^-$** in the presence of 100 eq. of DPTA at room temperature for 7 days; **c** Extracted UPLC-HRMS spectrum of **[GdL1]$^-$** in the presence of 1000 eq. of DPTA at room temperature for 7 days. The concentration of the **[GdL1]$^-$** was 14.8 μM in 0.1 M ammonium acetate.

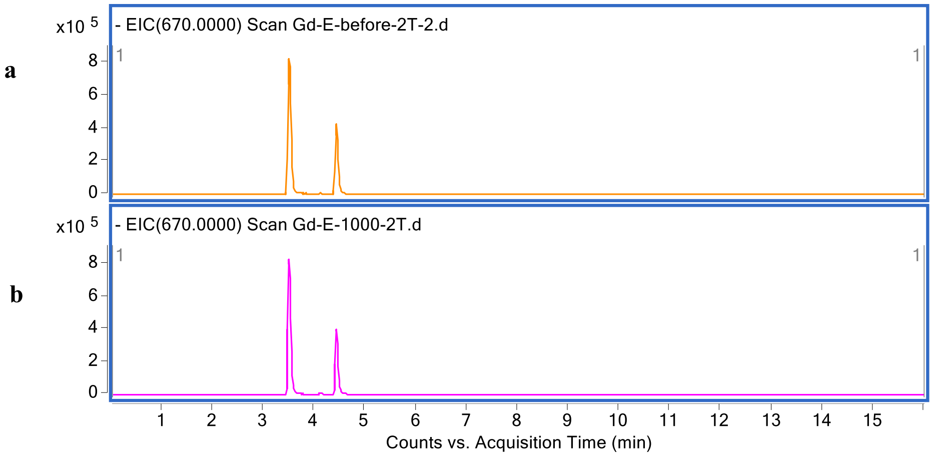

Supplementary Figure 8 | Competitive batch titration of [GdL2]⁻. a Extracted UPLC-HRMS spectrum of **[GdL2]⁻** in absence of DPTA; **b** Extracted UPLC-HRMS spectrum of **[GdL2]⁻** in the presence of 1000 eq. of DPTA at room temperature for 7 days. The concentration of the **[GdL2]⁻** was 21.6 μM in 0.1 M ammonium acetate.

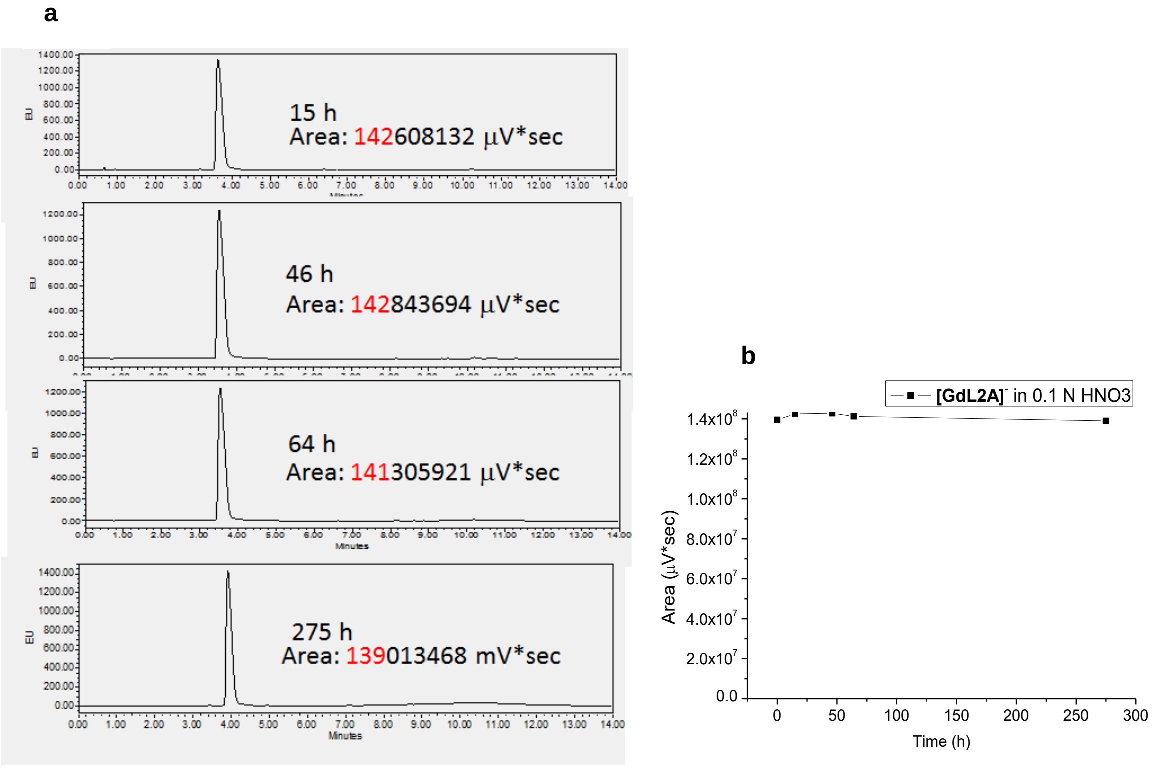

Supplementary Figure 9 | Stability test of [GdL2A]⁻ in 0.1 N HNO₃. a RP-HPLC trace of **[GdL2A]⁻** in 0.1 N HNO₃; **b** The variation of the integral area. Sample: 5.7 mg of **[GdL2A]⁻** in 1 ml 0.1 N HNO₃, using the condition A, injection volume 5 μl (the instrument was calibrated by **[GdL2A]⁻** and **[GdL2B]⁻** in water every day).

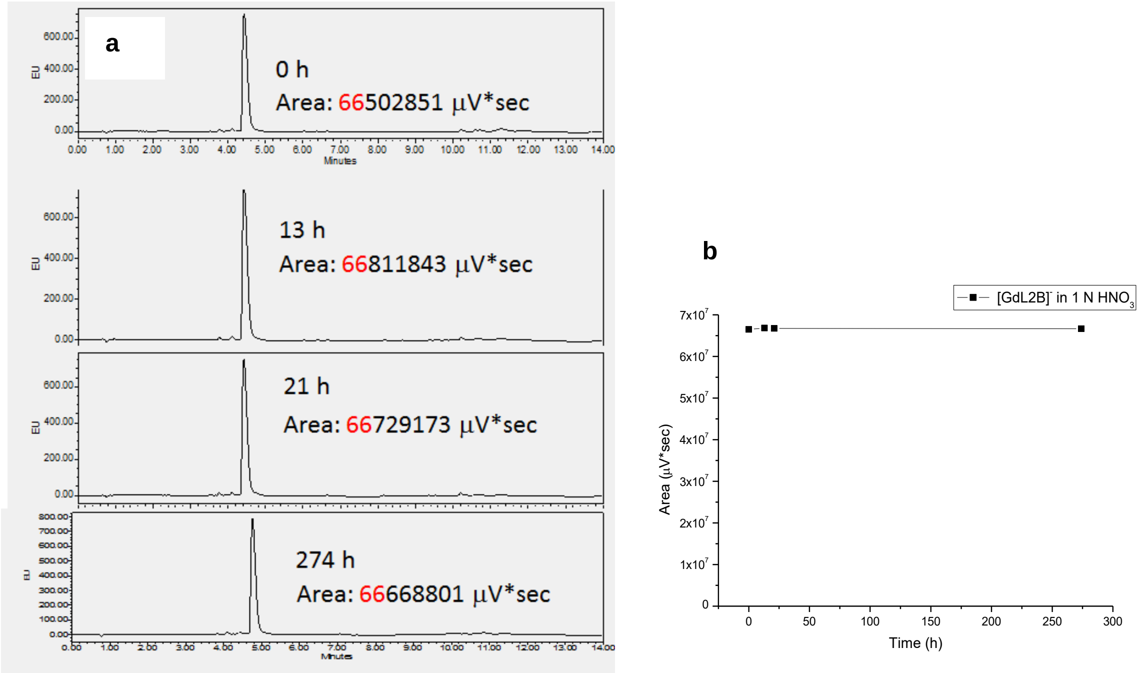

Supplementary Figure 10 | Stability test of [GdL2B]⁻ in 0.1 N HNO_3. a RP-HPLC trace of **[GdL2B]⁻** in 0.1 N HNO3; **b** The variation of the integral area. Sample: 1.7 mg of **[GdL2B]⁻** in 1 ml 0.1 N HNO_3, using the condition A, injection volume 10 µl (the instrument was calibrated by **[GdL2A]⁻** and **[GdL2B]⁻** in water every day).

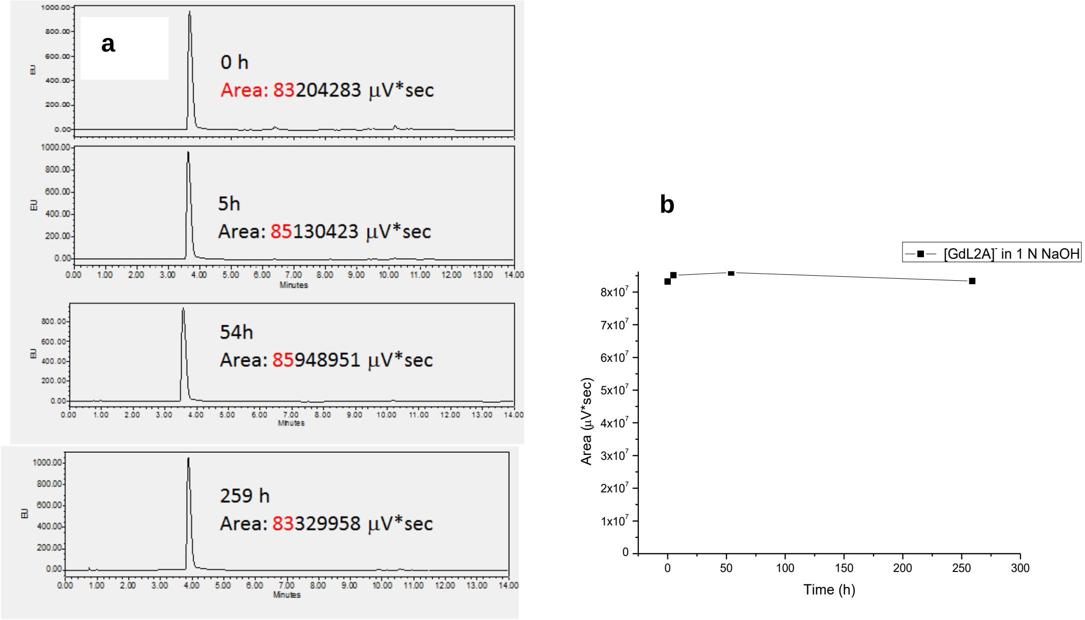

Supplementary Figure 11 | Stability test of [GdL2A]⁻ in 0.1 N NaOH. a RP-HPLC trace of **[GdL2A]⁻** in 0.1 N NaOH; **b** The variation of the integral area. Sample: 1.9 mg of **[GdL2A]⁻** in 1 ml 0.1 NaOH, using the condition A, injection volume 10 µl (the instrument was calibrated by **[GdL2A]⁻** and **[GdL2B]⁻** in water every day).

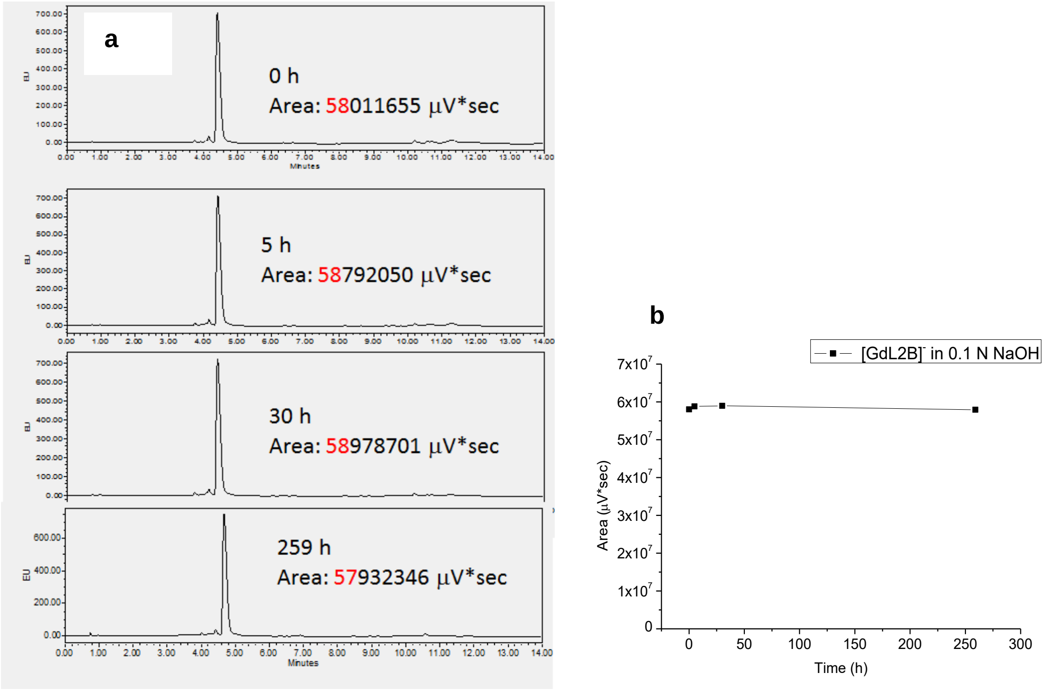

Supplementary Figure 12 | Stability test of [GdL2B]$^-$ in 0.1 N NaOH. a RP-HPLC trace of **[GdL2B]$^-$** in 0.1 N NaOH; **b** The variation of the integral area. Sample: 1.5 mg of **[GdL2B]$^-$** in 1 ml 0.1 N NaOH, using the condition A, injection volume 10 µl (the instrument was calibrated by **[GdL2A]$^-$** and **[GdL2B]$^-$** in water every day).

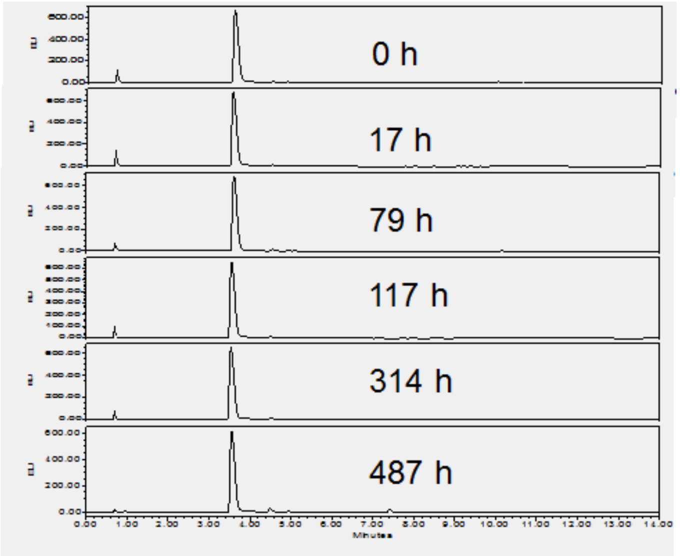

Supplementary Figure 13 | RP-HPLC trace of [GdL2A]$^-$ in 1 N HCl. Sample: 1 mg in 1 ml 1 N HCl, injection volume 10 µl.

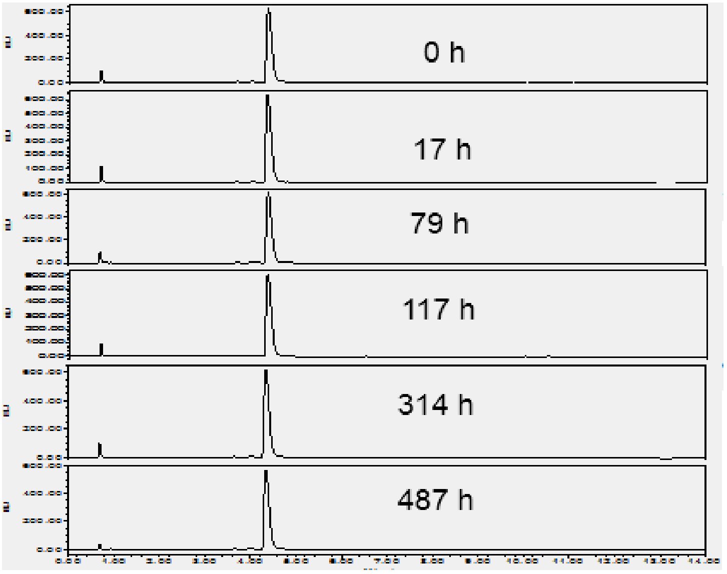

Supplementary Figure 14 | RP-HPLC trace of [GdL2A]$^-$ in 1 N HCl. Sample: 2.6 mg **[GdL2B]$^-$** in 1 ml 1 N HCl, Injection volume 5 µl.

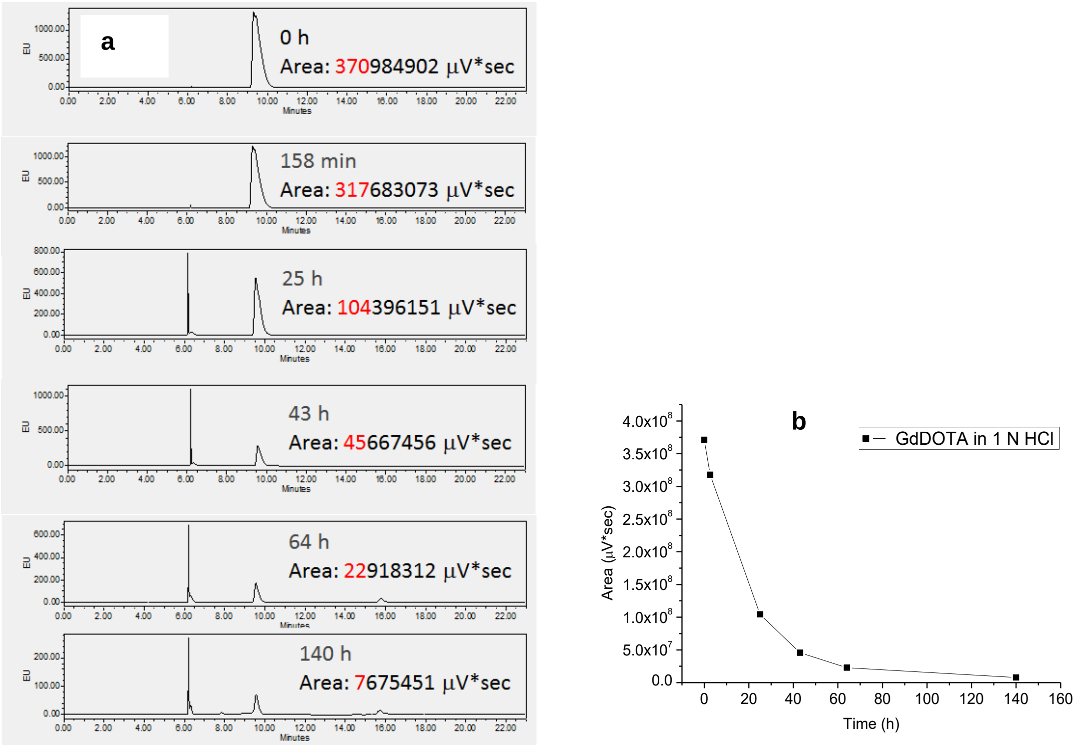

Supplementary Figure 15 | Stability test of [GdDOTA]$^-$ in 1 N HCl. a RP-HPLC trace of [GdDOTA]$^-$ in 1 N HCl; **b** The variation of the integral area. Sample: 15 µl of 0.5 M [GdDOTA]$^-$ (Dotarem®) diluted with 1.5 ml of 1 N HCl. Using the condition B (used the HILC column to extend the retention time). Injection volume 5 µl. In the main text for the ease of comparison, the integral areas were divided 10 (the profile was confirmed by another batch of sample as a positive control with different concentration of complexes under the same conditions, the instrument was calibrated by [GdDOTA]$^-$ in water every day).

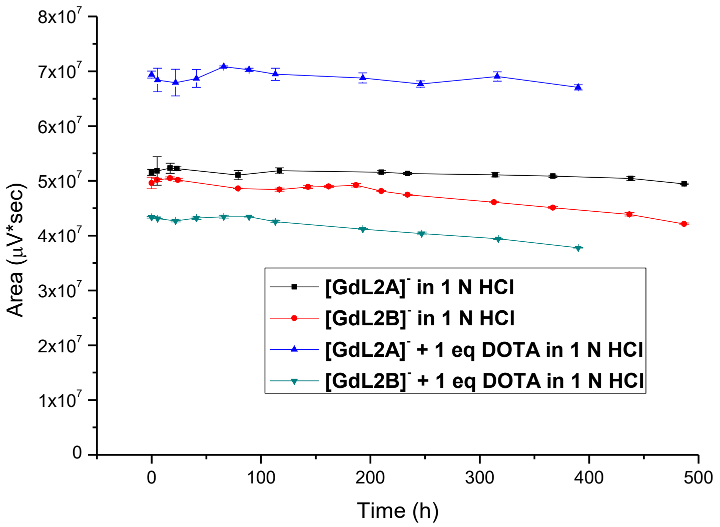

Supplementary Figure 16 | Stability test of [GdL2A]$^-$ and [GdL2B]$^-$ in 1N HCl. Variation of the integral areas of **[GdL2A]$^-$** and **[GdL2B]$^-$** in 1 N HCl with and without DOTA over time. Three injections of UPLC analysis were performed per each point, error bars indicate the standard error of mean (S.E.M.).

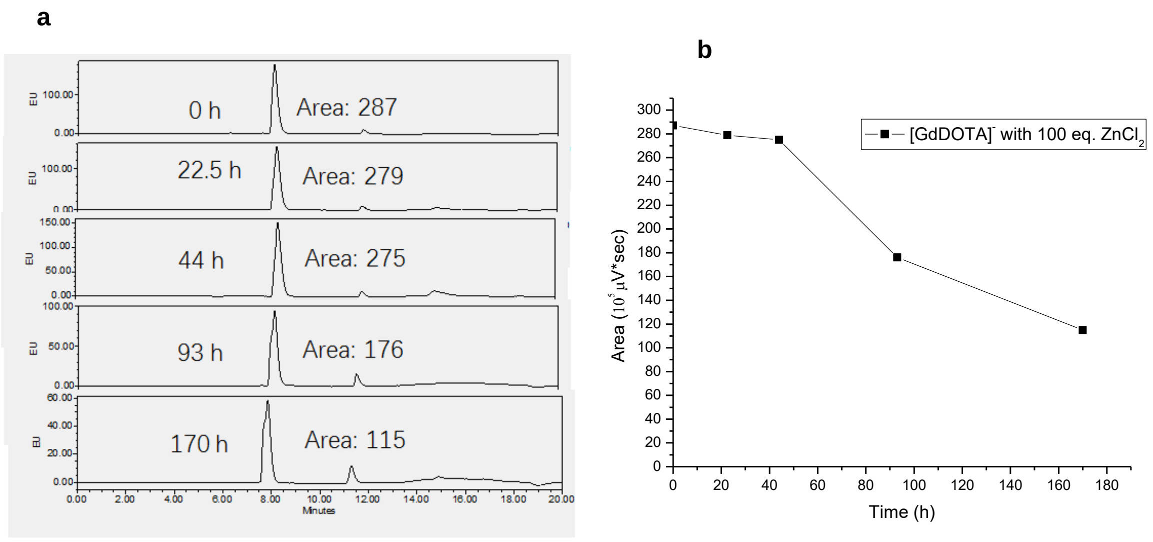

Supplementary Figure 17 | Stability test of [GdDOTA]$^-$ over Zn(II). a RP-HPLC trace of [GdDOTA]$^-$ in the presence of 100 equivalents of $ZnCl_2$; **b** The variation of the integral area. Sample: 5.8 µl of 0.5 M [GdDOTA]$^-$ (Dotarem®) diluted with 1.5 ml of $ZnCl_2$ (39.6 mg) solution then put on the shaker at 50 °C. Using the condition B, injection volume 10 µl. The integral area was divided by 10^5 for clarity (the profile was confirmed by another batch with different concentration of complex under the same conditions, the instrument was calibrated by [GdDOTA]$^-$ in water every day).

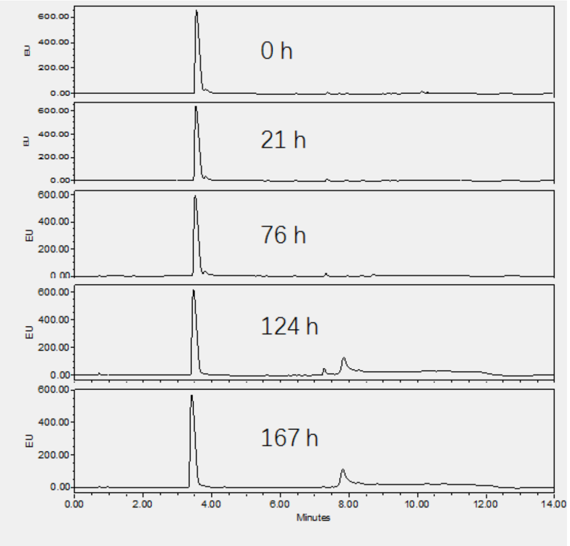

Supplementary Figure 18 | Stability test of [GdL2A]$^-$ over Zn(II). RP-HPLC trace of **[GdL2A]$^-$** in the presence of 100 equivalents of $ZnCl_2$. Sample: 2.0 mg of sample was dissolved in 1.5 ml of $ZnCl_2$ (39.6 mg) solution then put on the shaker at 50 °C, using the condition B, injection volume 10 µl.

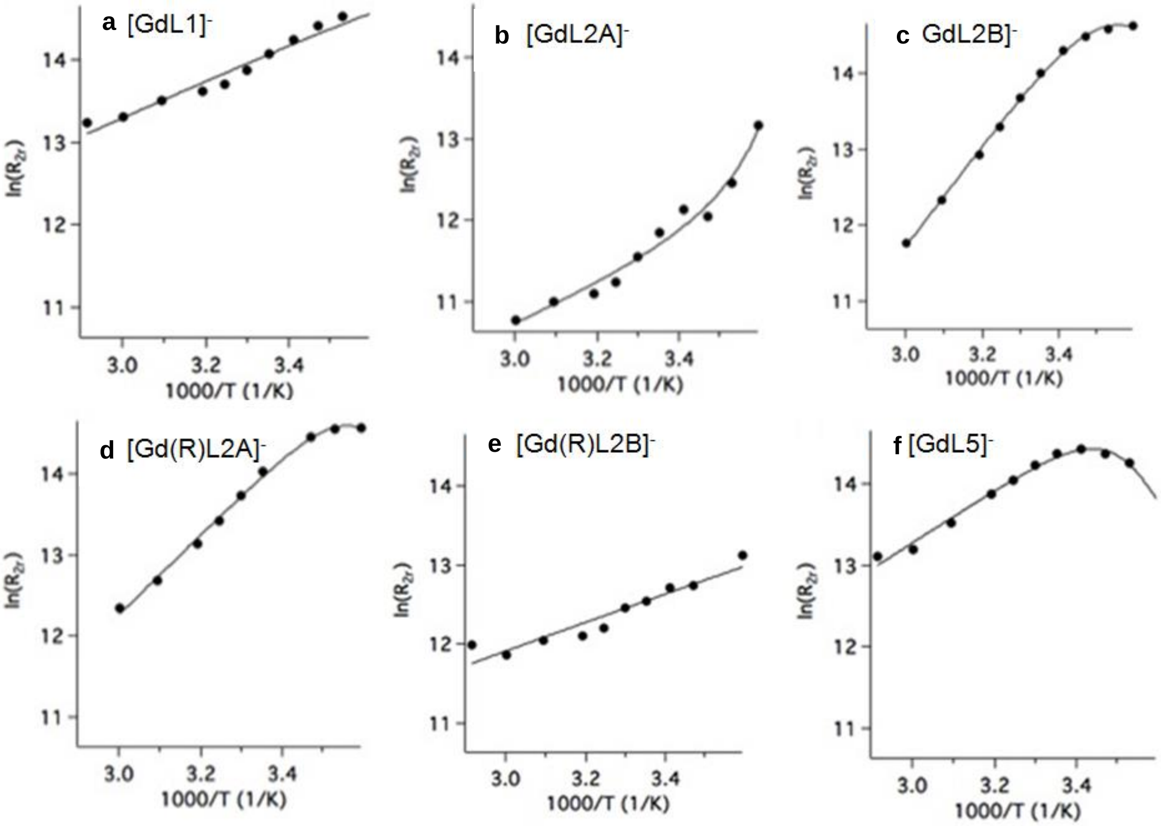

Supplementary Figure 19 | Fitting profiles of the $H_2{}^{17}O$ reduced relaxation rates. Temperature dependence on the reduced $H_2{}^{17}O$ transverse relaxation rates of solutions of **[GdL1]**$^-$, **[GdL2A]**$^-$, **[GdL2B]**$^-$, **[GdL(*R*)2A]**$^-$, **[GdL(*R*)2B]**$^-$ and **[GdL5]**$^-$ are shown in **a** – **f**, respectively (the temperature was calibrated by measuring the chemical shift difference between CH_2 and OH peaks of ethylene glycol).

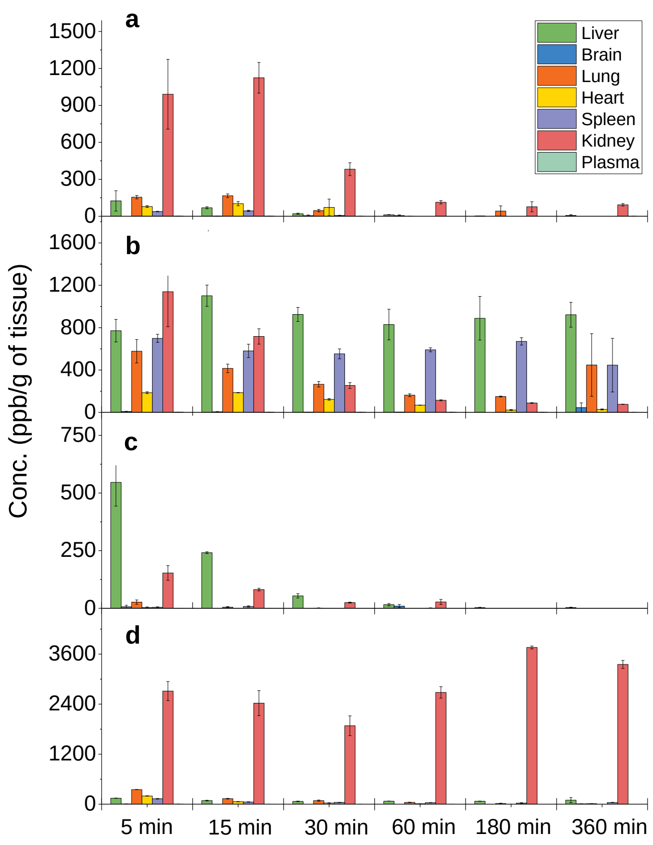

Supplementary Figure 20 | The biodistribution results of Gd(III) complexes. The biodistribution of [GdDOTA]$^-$, [GdL1]$^-$, [GdL2]$^-$ and [GdL5]$^-$ in mice over time are shown in **a** – **d.** Three mice per compound per time point were euthanized and the concentrations of the Gd in different tissues were determined by ICP-MS; error bars indicate the standard error of mean (S.E.M.).

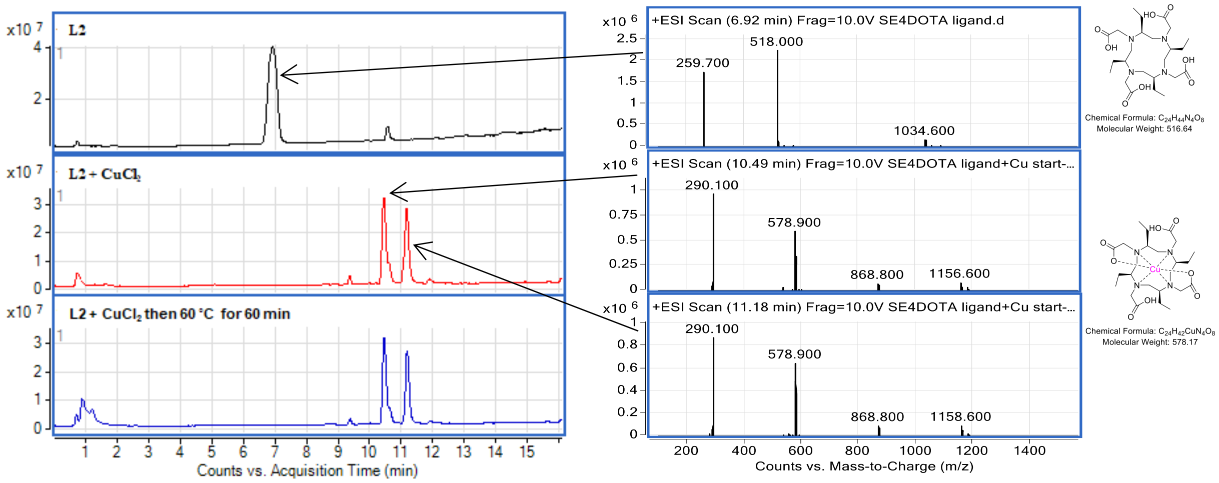

Supplementary Figure 21 | Cold labelling of Cu(II) with L2. Ligand **L2** (0.4 mg) (final 247 μM) in 3 ml of 1 M NaOAc buffer (pH 6.0), the solution of $CuCl_2$ (6.6 μL, 1.0 eq.) in water was added into the mixture, took out 50 μL each time and diluted to 500 mL for analysis. LCMS of the ligand **L2**, LC-MS after mixing with Cu(II) about one minute at RT and heating at 60 °C for 60 minutes. (MS detector)

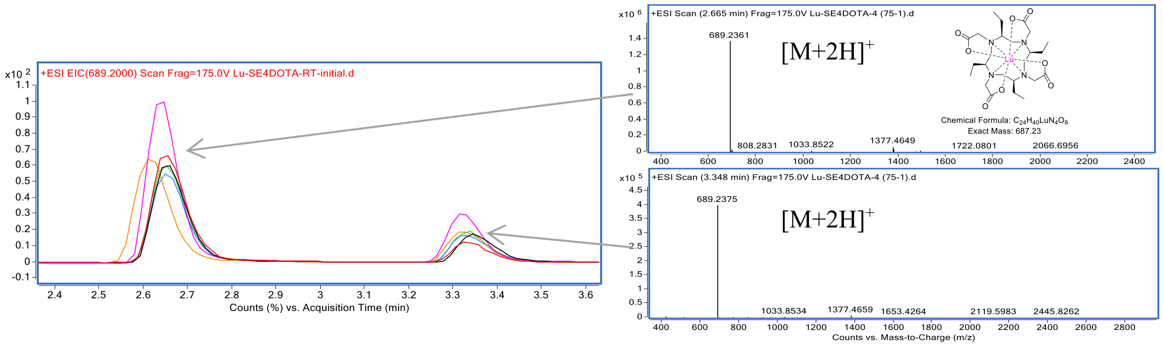

Supplementary Figure 22 | Cold labelling of Lu(III) with L2 in HEPES buffer. Condition: Ligand / metal = 1: 1, HEPES buffer (0.1 M, pH 7.02), ligand concentration 1.2 × 10^{-4} M. Monitoring the MS of the complex by LCMS. RT 1 h, then 37 °C for 40 min, then 75 °C for 15 min, then 80 °C for 18 h (the highest peak corresponding to the reaction of 80 °C, 18 h). The results showed the complexation occurred at room temperature, however, need more metal or higher temperature to get fully complexation.

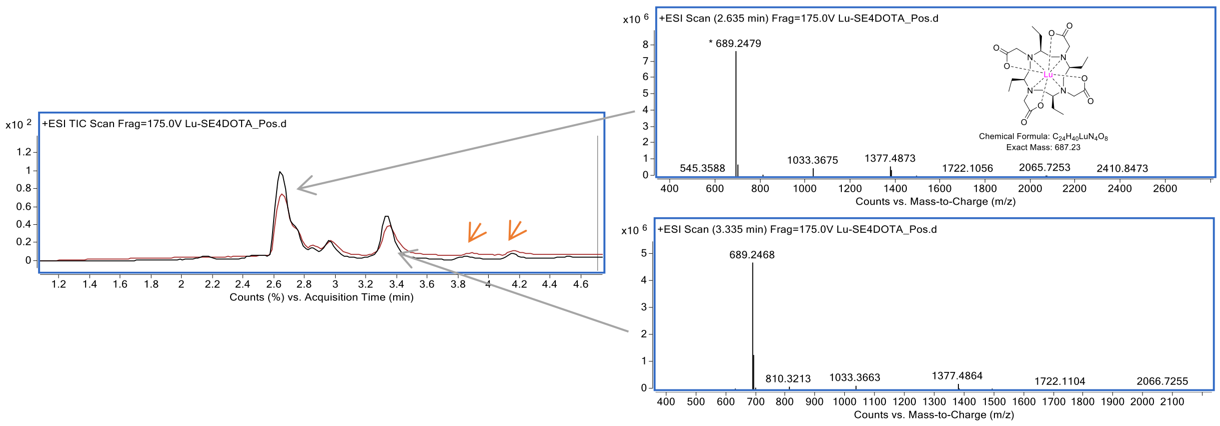

Supplementary Figure 23 | Cold labelling of Lu(III) with L2 in TRIS buffer. Condition: Ligand / metal = 1: 1, TRIS buffer (0.1 M, pH 7.01), ligand concentration 2.1 × 10^{-4} M, 85 °C for 1 h. Monitored by LCMS, the ligand is easily coordinate with metal ions, especially for potassium cation (two peaks). The orange arrows indicate ligand plus K, and no ligand signal of 517.3 was observed.

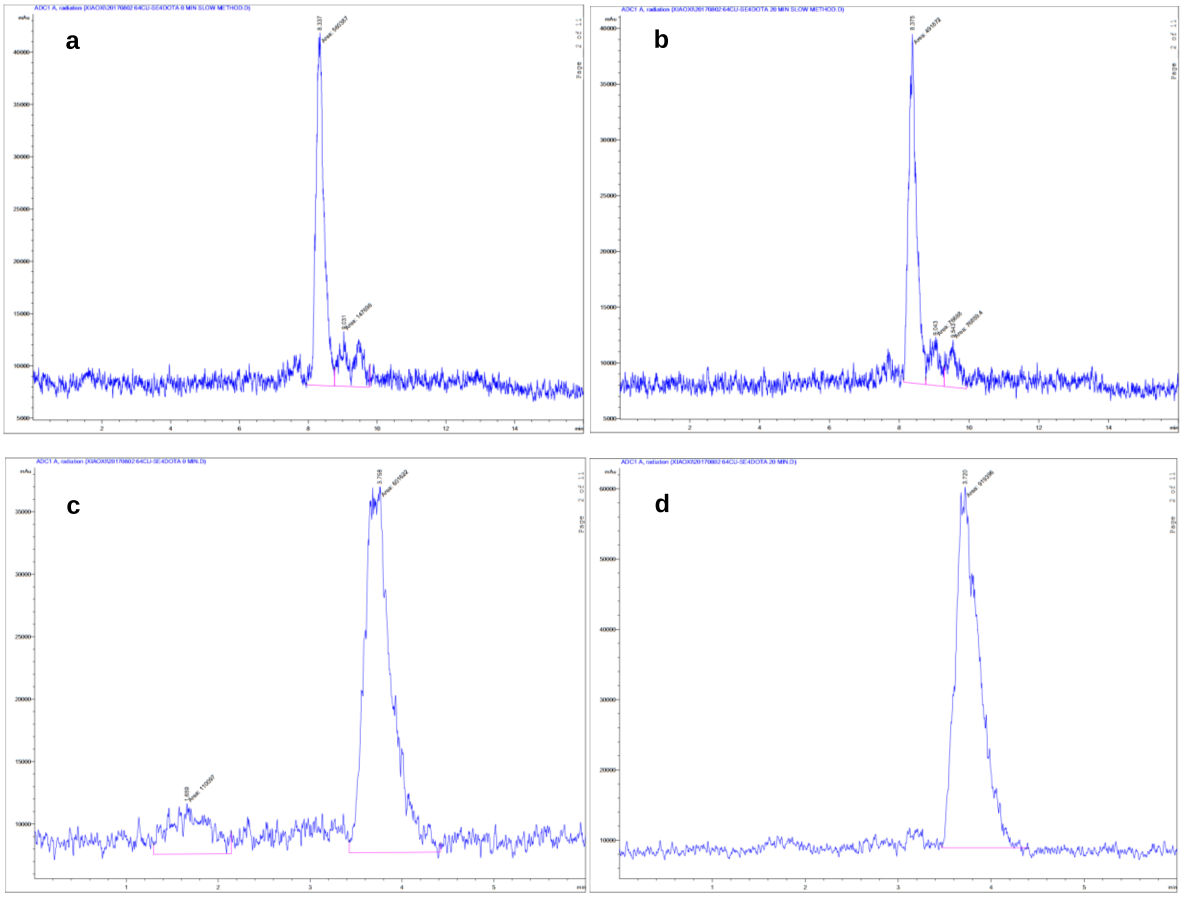

Supplementary Figure 24 | Radio-HPLC chromatograms of ^{64}CuL2. a HPLC chromatograms of **^{64}CuL2** after reaction in pH 6.05 at 40 °C for 0 min; **b** HPLC chromatograms of **^{64}CuL2** after reaction in pH 6.05 at 40 °C for 20 min. **c** HPLC chromatograms of **^{64}CuL2** using rapid HPLC method after reaction in pH 6.05 at 40 °C for 0 min; **d** HPLC chromatograms of **^{64}CuL2** using rapid HPLC method after reaction in pH 6.05 at 40 °C for 20 min.

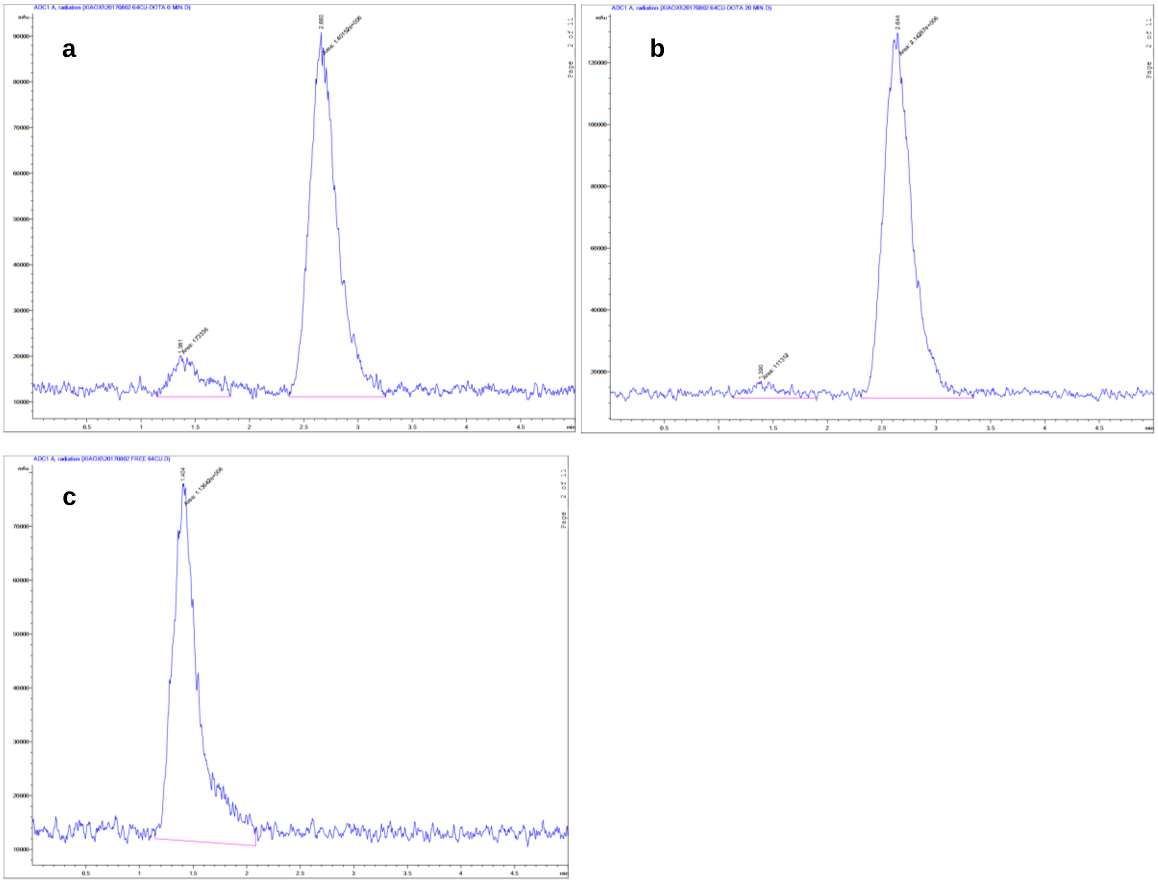

Supplementary Figure 25 | Radio-HPLC chromatograms of ^{64}CuDOTA. a HPLC chromatograms of ^{64}CuDOTA after reaction in pH 6.05 at 40 °C for 0 min; **b** HPLC chromatograms of ^{64}CuDOTA after reaction in pH 6.05 at 40 °C for 20 min; **c** HPLC chromatograms of $^{64}CuCl_2$ in pH 6.05.

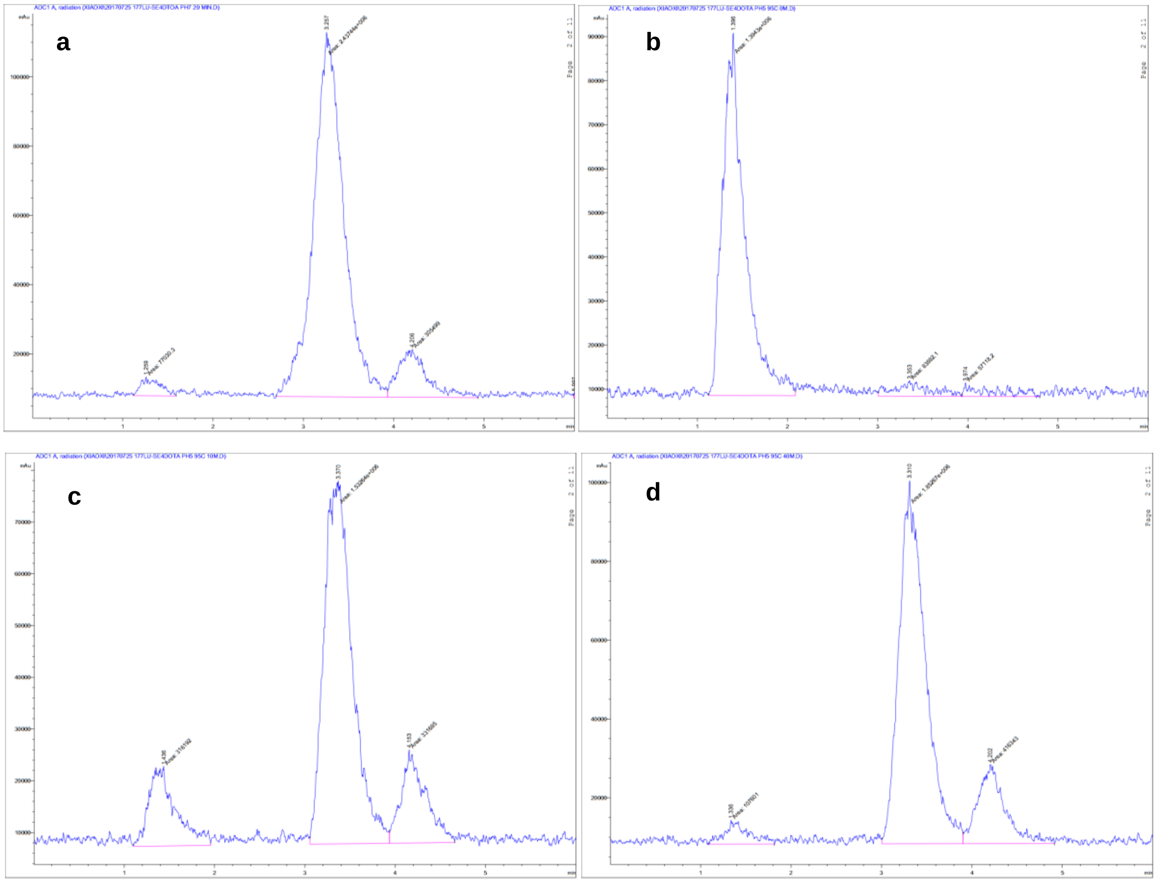

Supplementary Figure 26 | Radio-HPLC chromatograms of $[^{177}\mathrm{LuL2}]^-$. a HPLC chromatograms of **$[^{177}\mathrm{LuL2}]^-$** after reaction in pH 7.00 at 95 °C for 20 min; **b** HPLC chromatograms of **$[^{177}\mathrm{LuL2}]^-$** after reaction in pH 5.01 at 95 °C for 0 min; **c** HPLC chromatograms of **$[^{177}\mathrm{LuL2}]^-$** after reaction in pH 5.01 at 95 °C for 10 min; **d** HPLC chromatograms of **$[^{177}\mathrm{LuL2}]^-$** after reaction in pH 5.01 at 95 °C for 40 min.

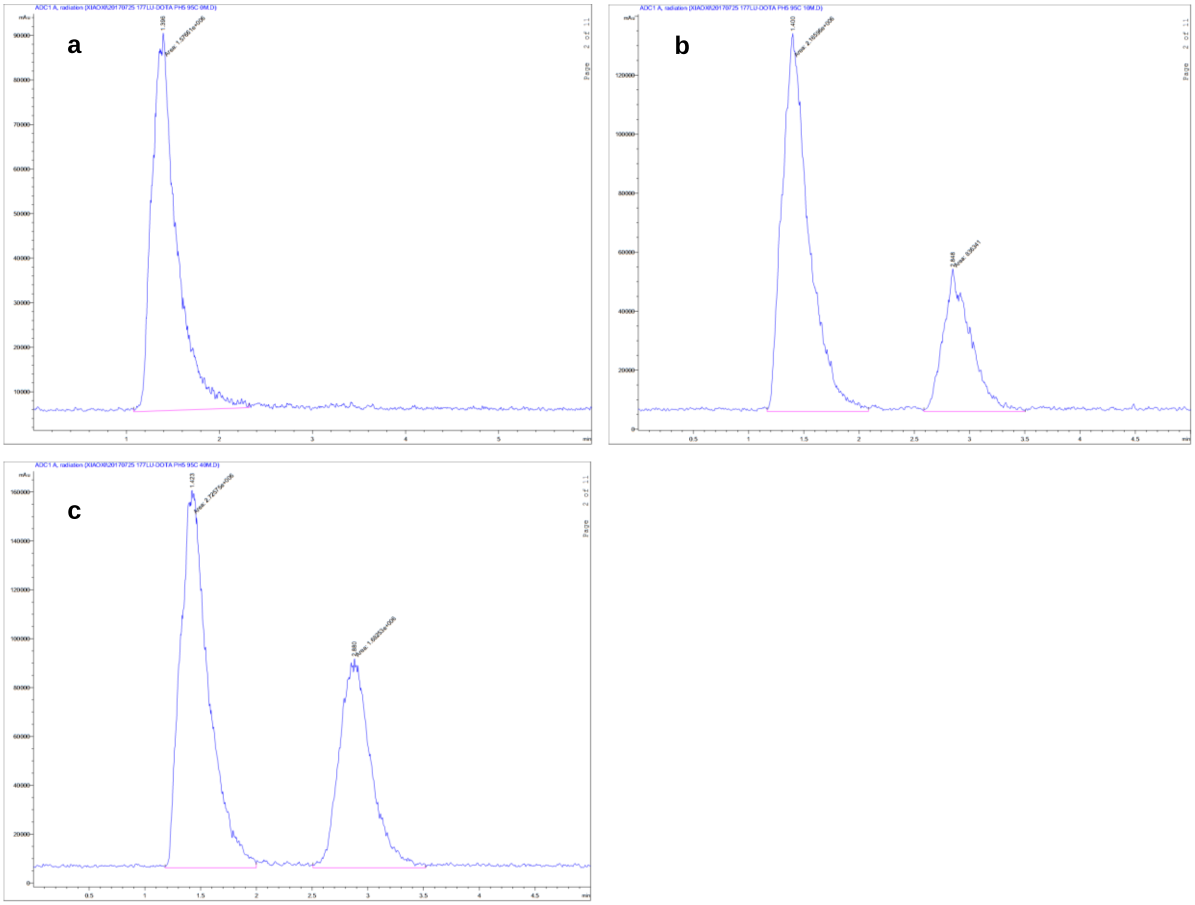

Supplementary Figure 27 | Radio-HPLC chromatograms of [^{177}LuDOTA]$^-$. a HPLC chromatograms of [^{177}LuDOTA]$^-$ after reaction in pH 5.01 at 95 °C for 0 min; **b** HPLC chromatograms of [^{177}LuDOTA]$^-$ after reaction in pH 5.01 at 95 °C for 10 min; **c** HPLC chromatograms of [^{177}LuDOTA]$^-$ after reaction in pH 5.01 at 95 °C for 40 min.

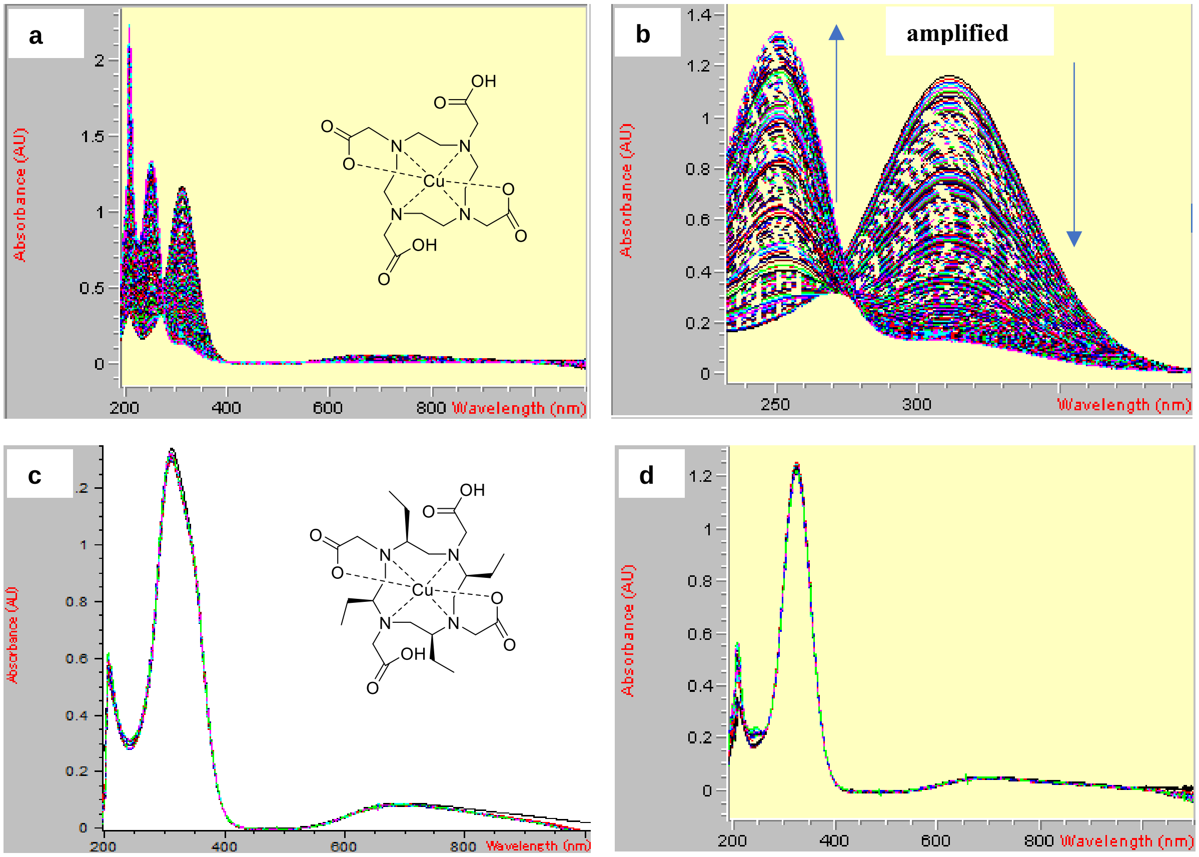

Supplementary Figure 28 | Comparison of the stability between CuDOTA and CuL2. The stability between CuDOTA and **CuL2** in 1 N HCl was compared by measuring their UV-visible spectra. **a** Variation of UV-Visible spectra of CuDOTA in 1 N HCl upon 30 h (10 mins a spectrum); **b** Amplified spectra of CuDOTA in 1 N HCl; **c** Variation of UV-Visible spectra of **CuL2** (1st peak) in 1 N HCl upon 30 h (1 h a spectrum); **d** Variation of UV-Visible spectra of **CuL2** (2nd peak) in 1 N HCl upon 30 h (1 h a spectrum).

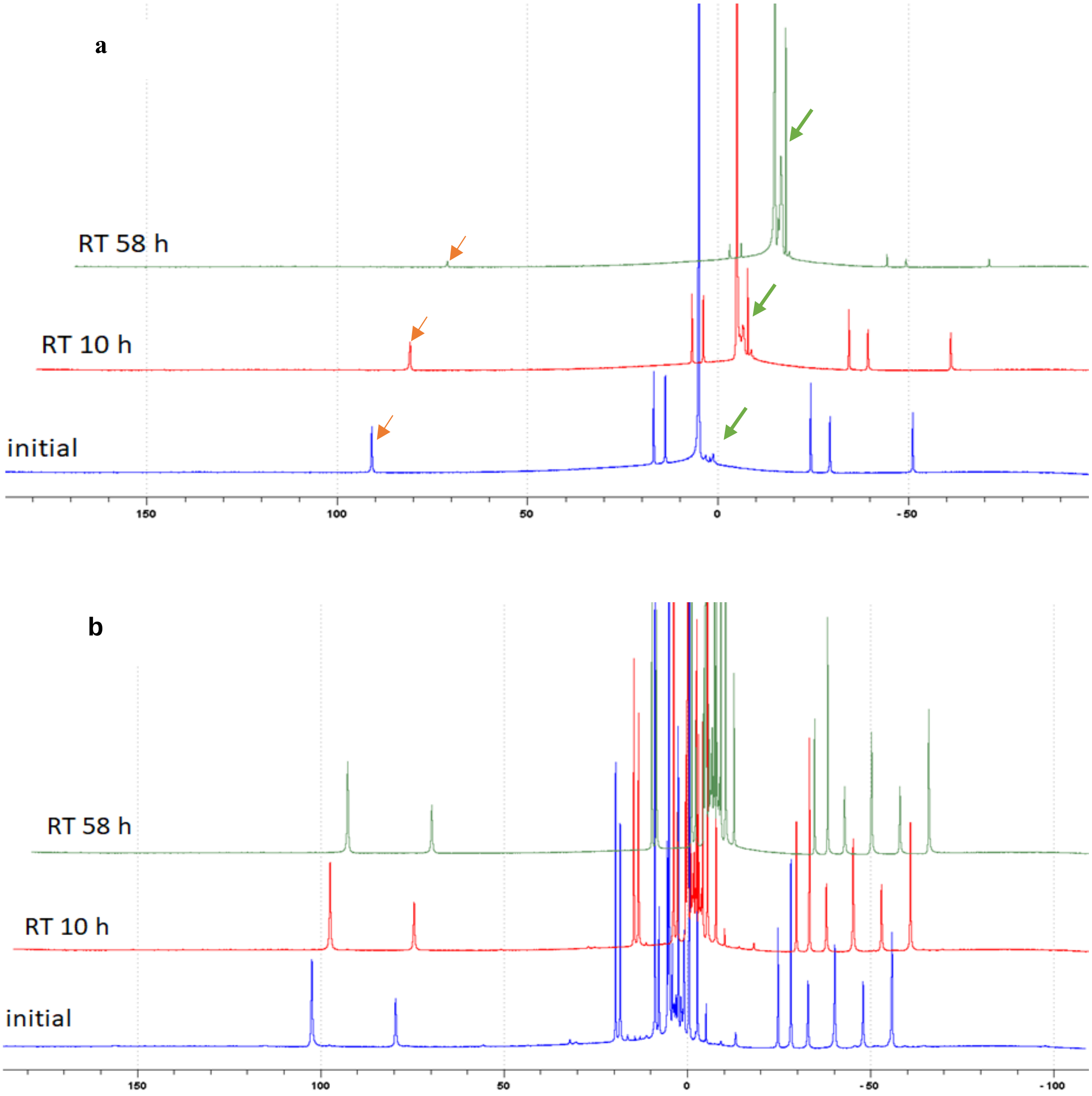

Supplementary Figure 29 | Comparison of the stability between [YbDOTA]⁻ and [YbL2]⁻. The stability between [YbDOTA]⁻ and **[YbL2]⁻** in 1 N DCl was compared by measuring their ^{1}H NMR spectra. **a** Variation of ^{1}H NMR spectra of [YbDOTA]⁻ in 1 N DCl upon time (the orange arrows show the characteristic complex peaks, the green arrows show the characteristic ligand peaks); **b** Variation of ^{1}H NMR spectra of **[YbL2]⁻** in 1 N DCl upon time (RT).

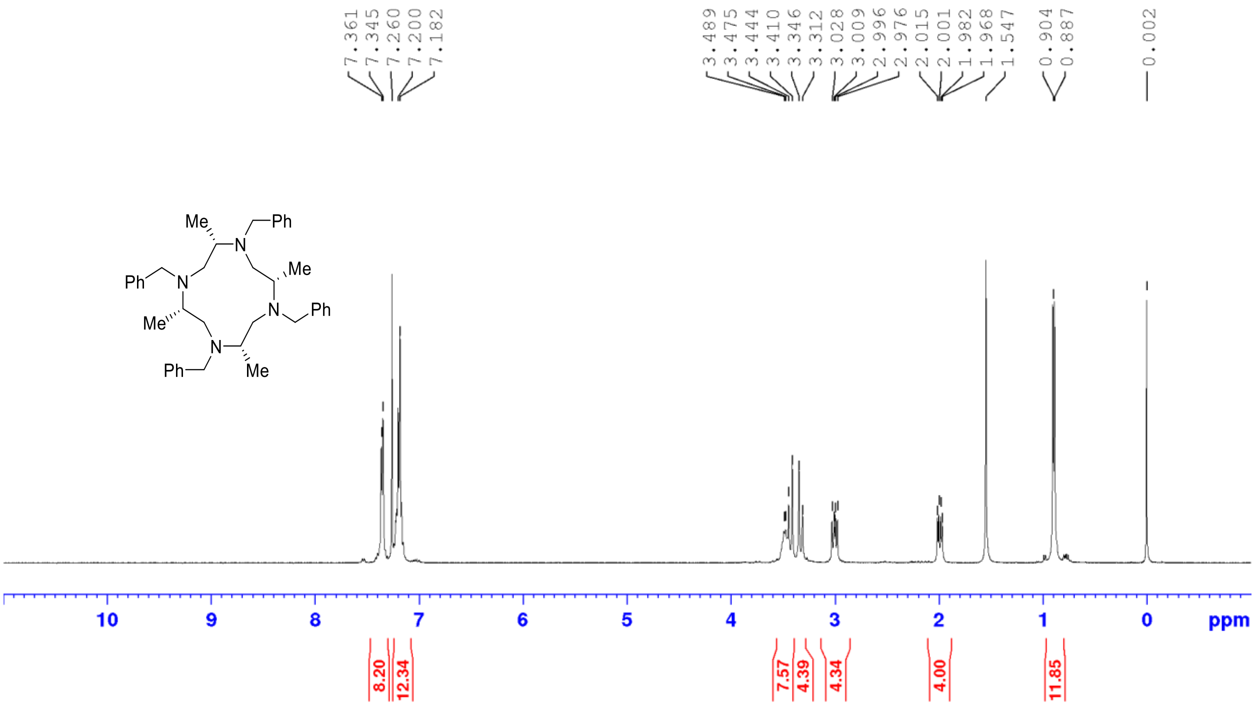

Supplementary Figure 30 | ^{1}H NMR spectrum of compound 2a.

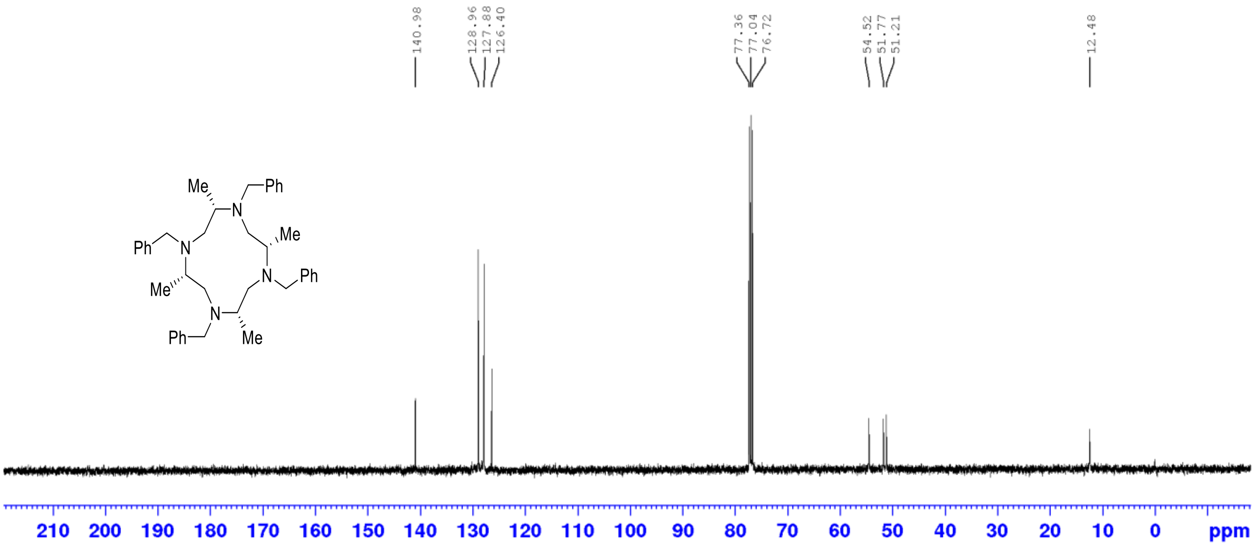

Supplementary Figure 31 | ^{13}C NMR spectrum of compound 2a.

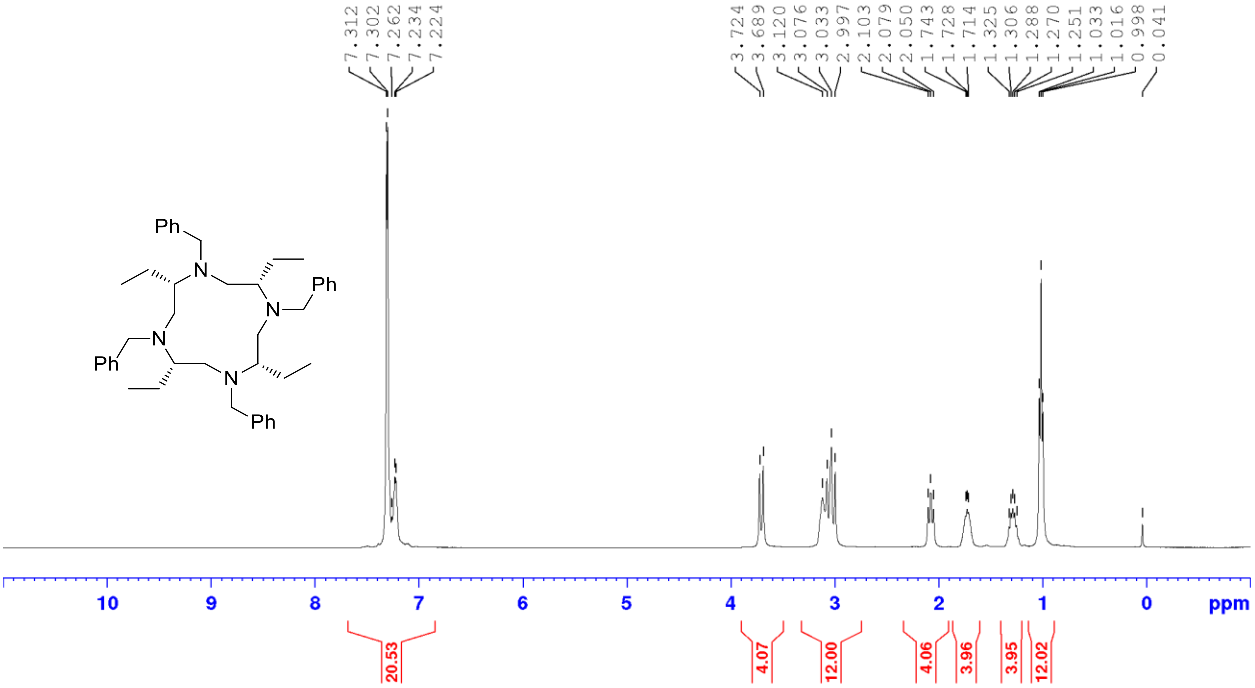

Supplementary Figure 32 | ^{1}H NMR spectrum of compound 2b.

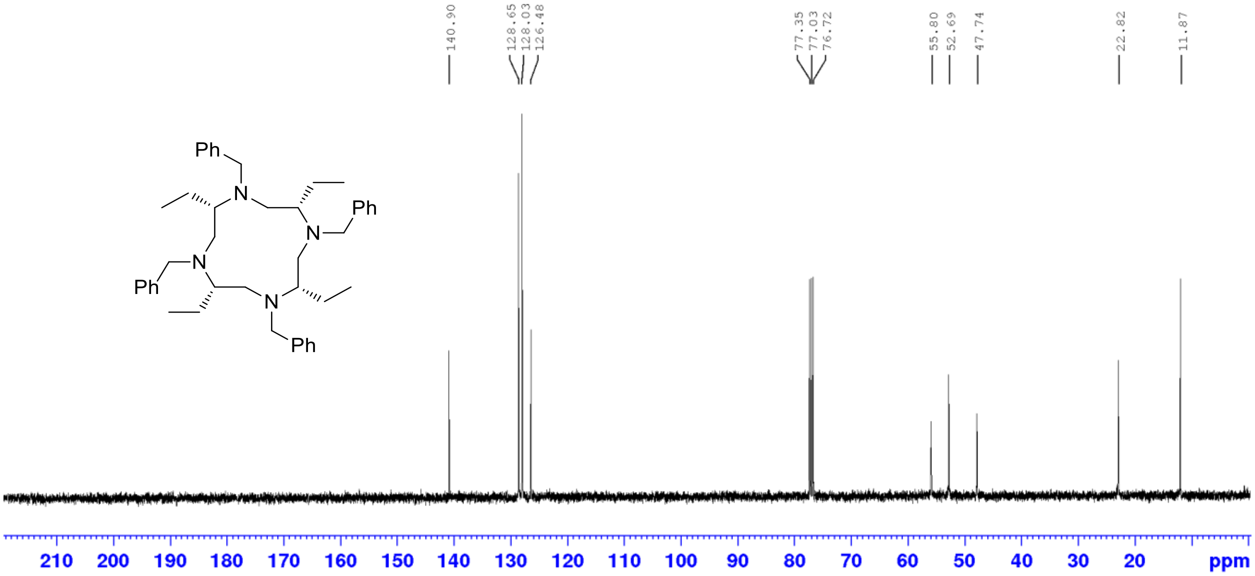

Supplementary Figure 33 | ^{13}C NMR spectrum of compound 2b.

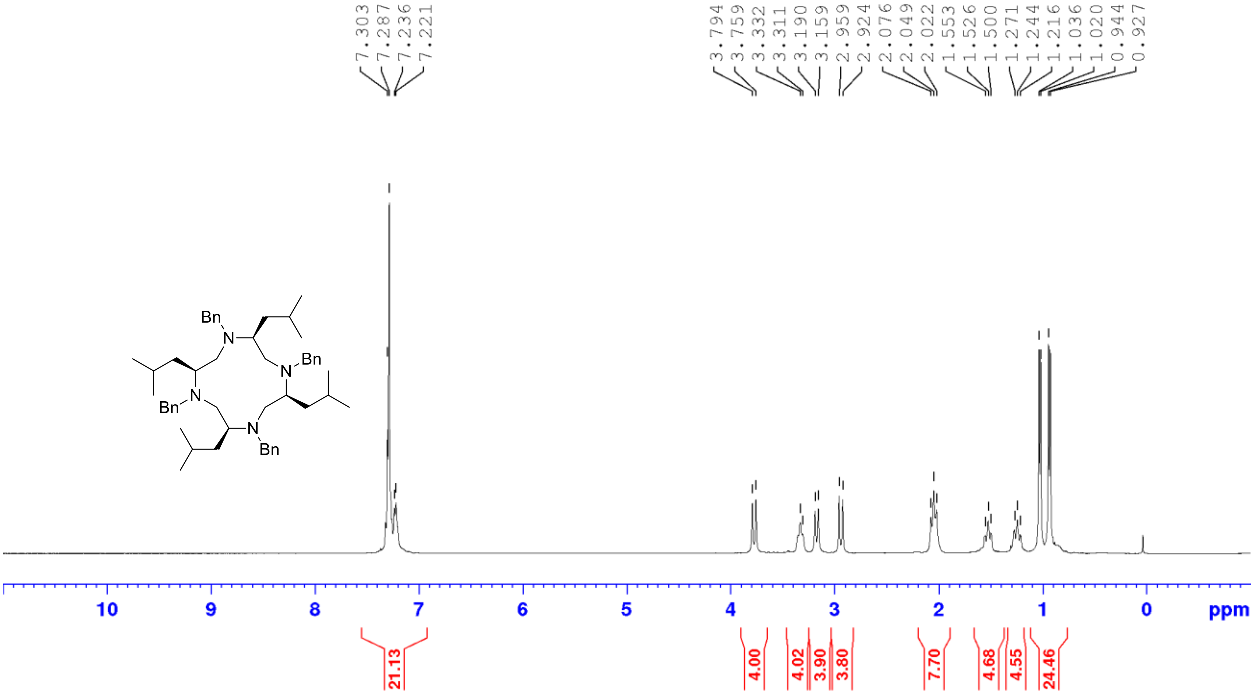

Supplementary Figure 34 | ^{1}H NMR spectrum of compound 2c.

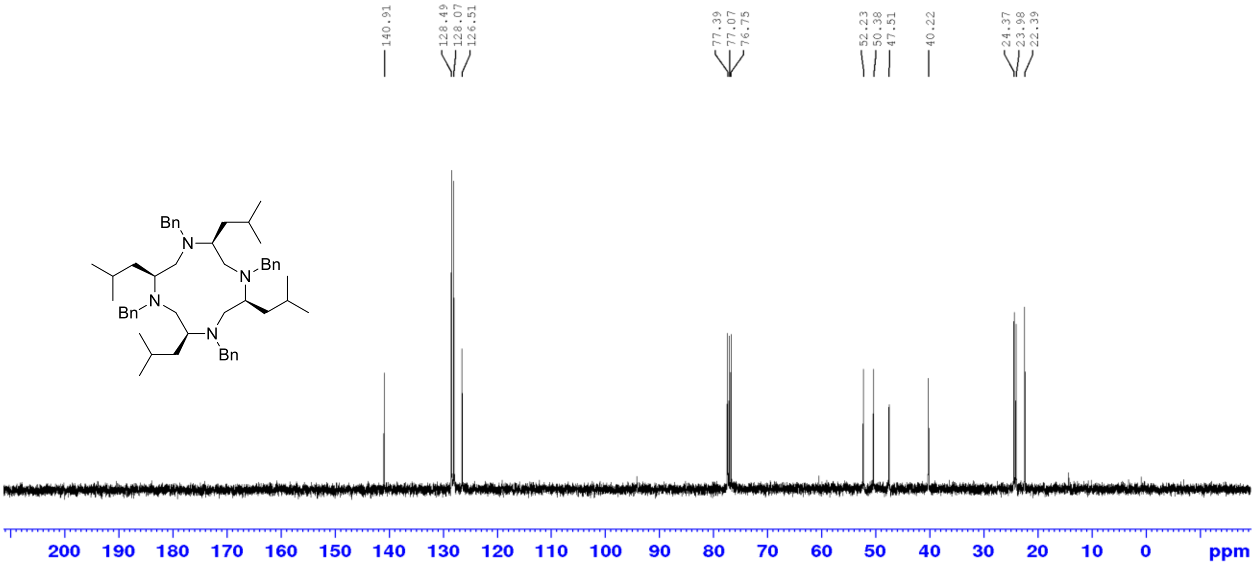

Supplementary Figure 35 | ^{13}C NMR spectrum of compound 2c.

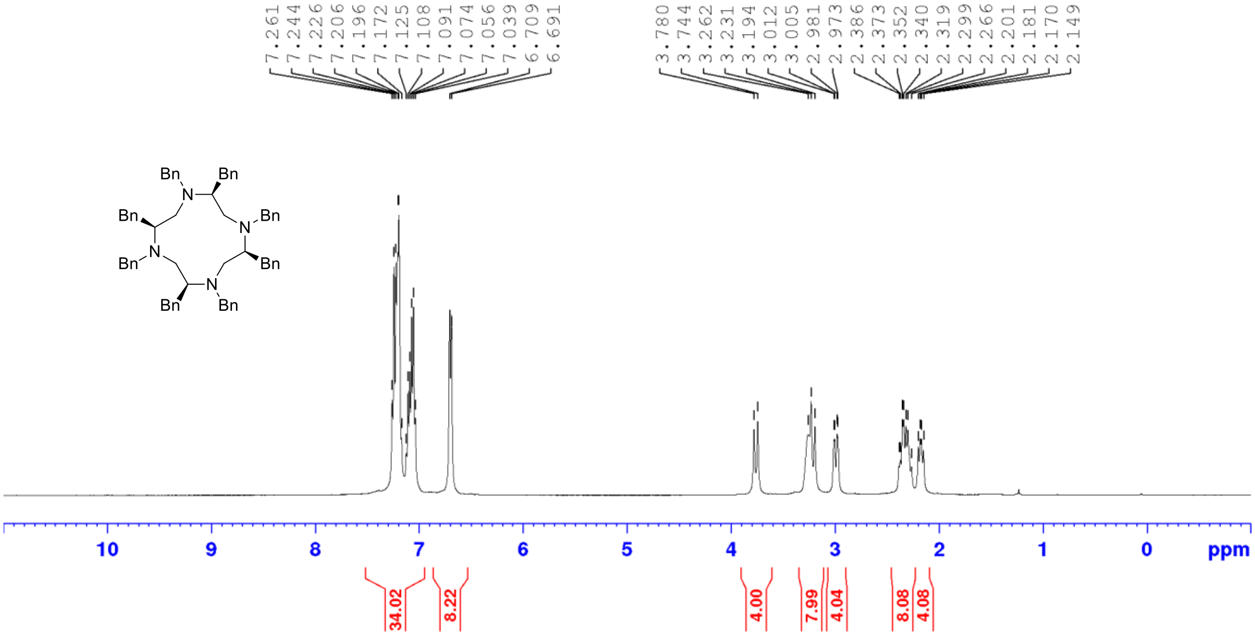

Supplementary Figure 36 | ^{1}H NMR spectrum of compound 2d.

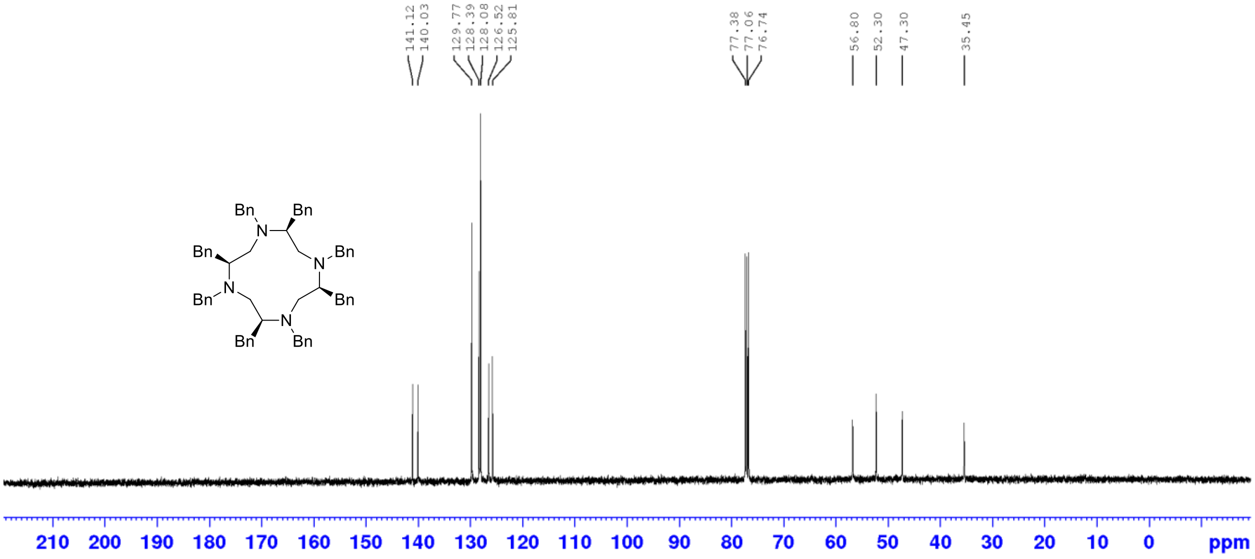

Supplementary Figure 37 | ^{13}C NMR spectrum of compound 2d.

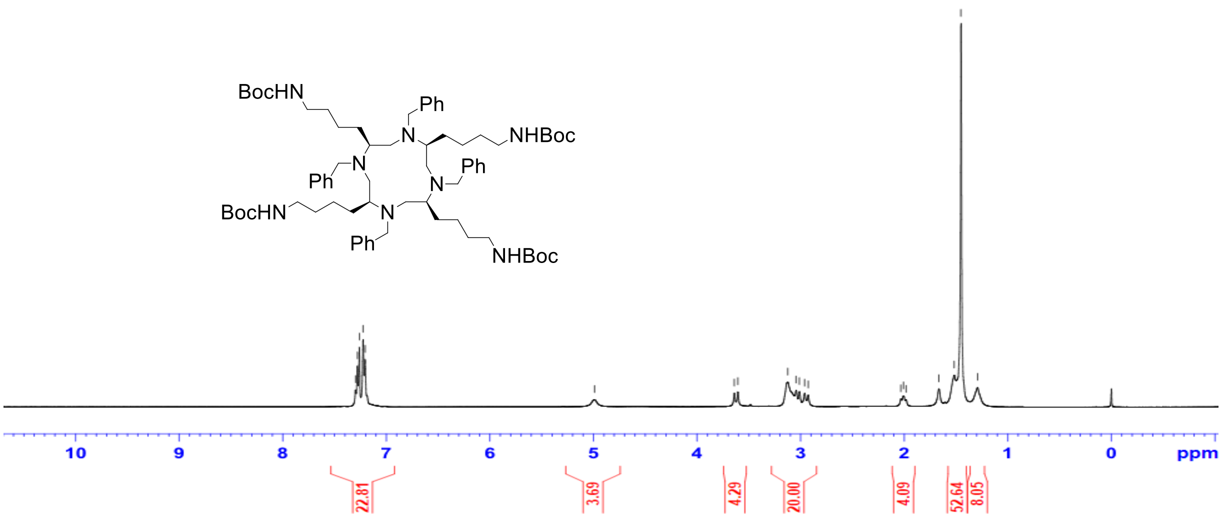

Supplementary Figure 38 | ^{1}H NMR spectrum of compound 2e.

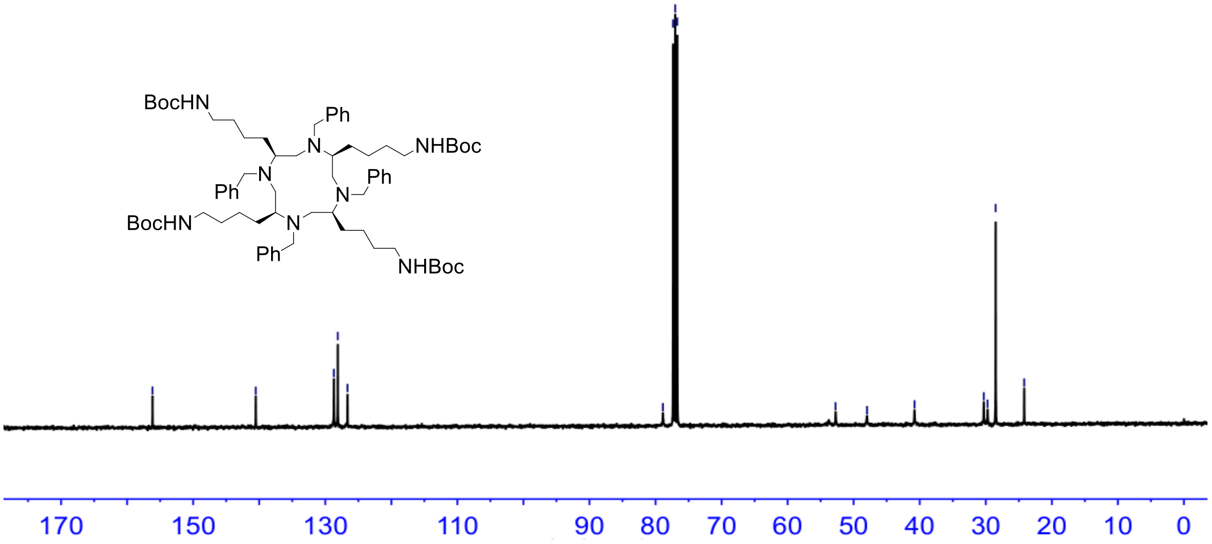

Supplementary Figure 39 | ^{13}C NMR spectrum of compound 2e.

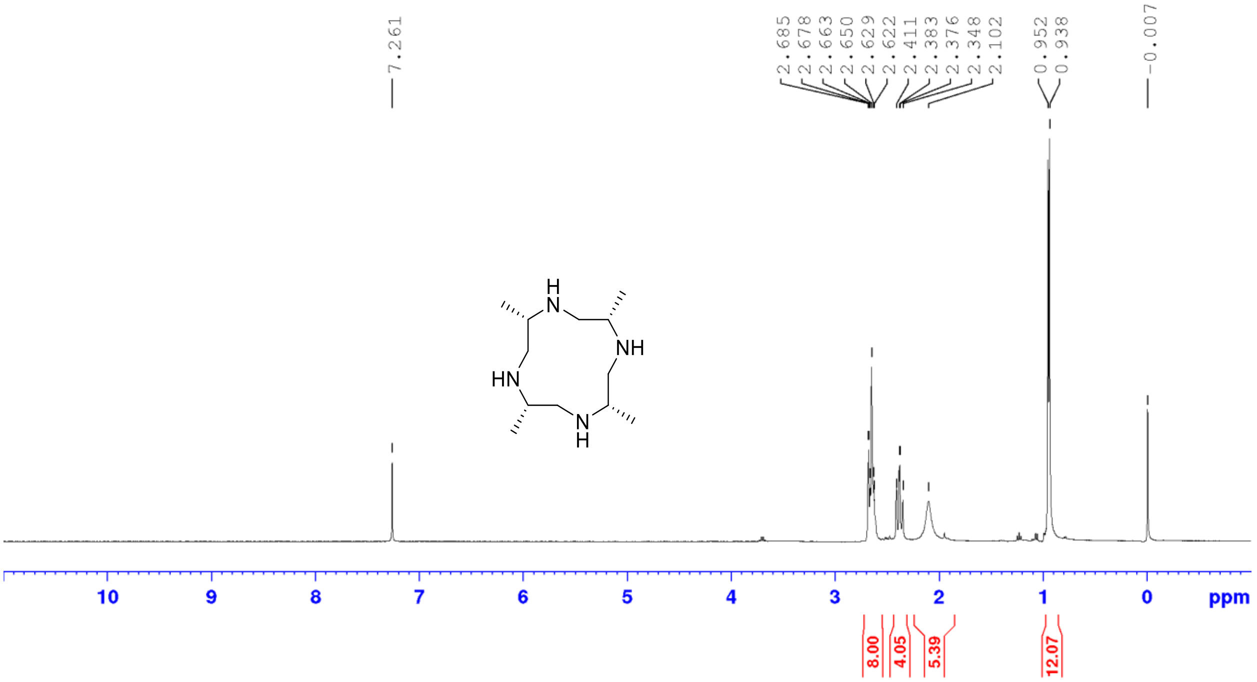

Supplementary Figure 40 | ^{1}H NMR spectrum of compound 3a.

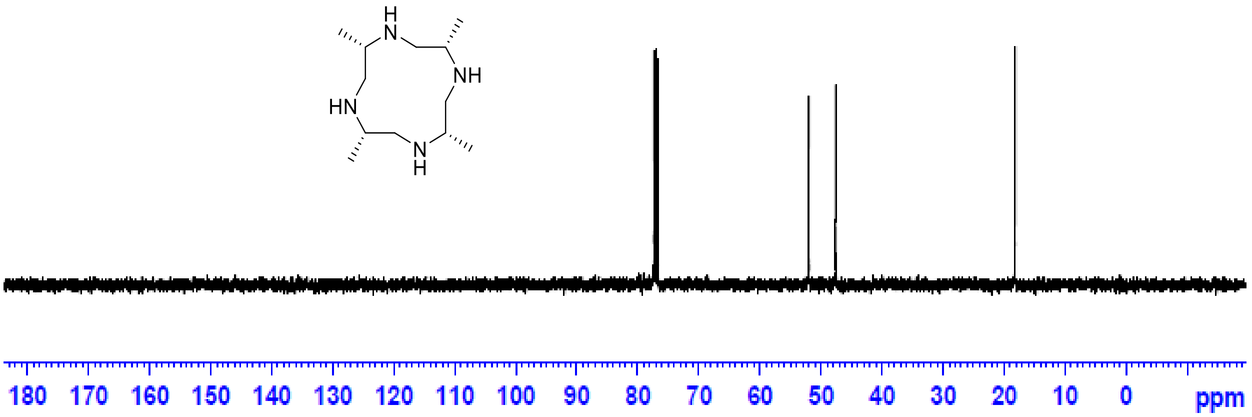

Supplementary Figure 41 | ^{13}C NMR spectrum of compound 3a.

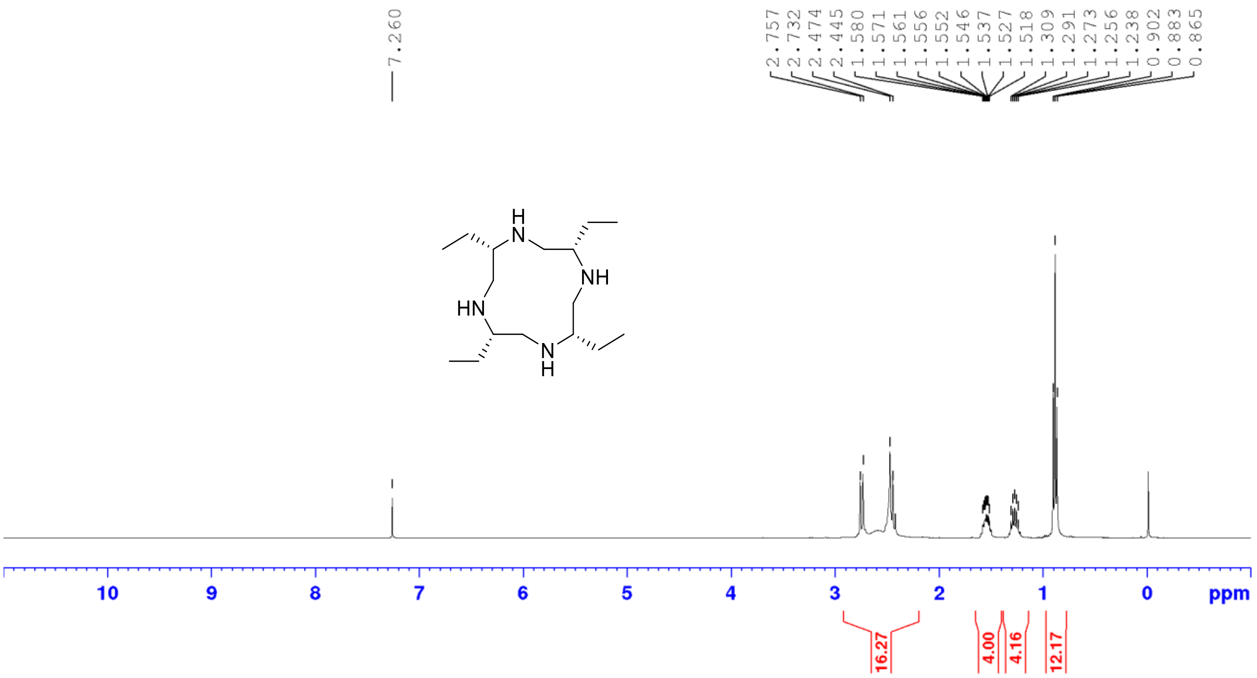

Supplementary Figure 42 | ^{1}H NMR spectrum of compound 3b.

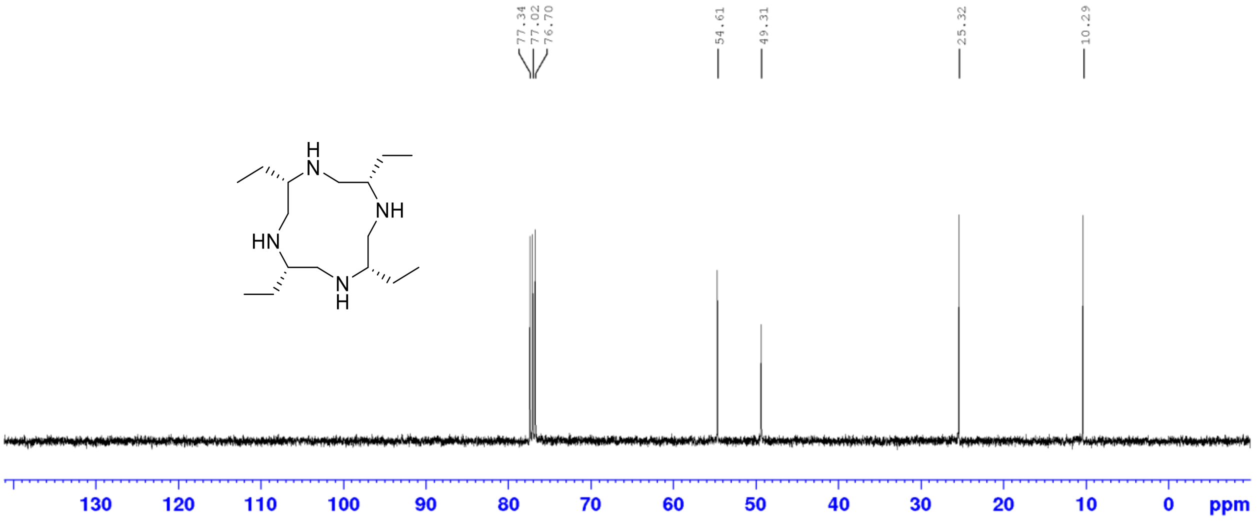

Supplementary Figure 43 | ^{13}C NMR spectrum of compound 3b.

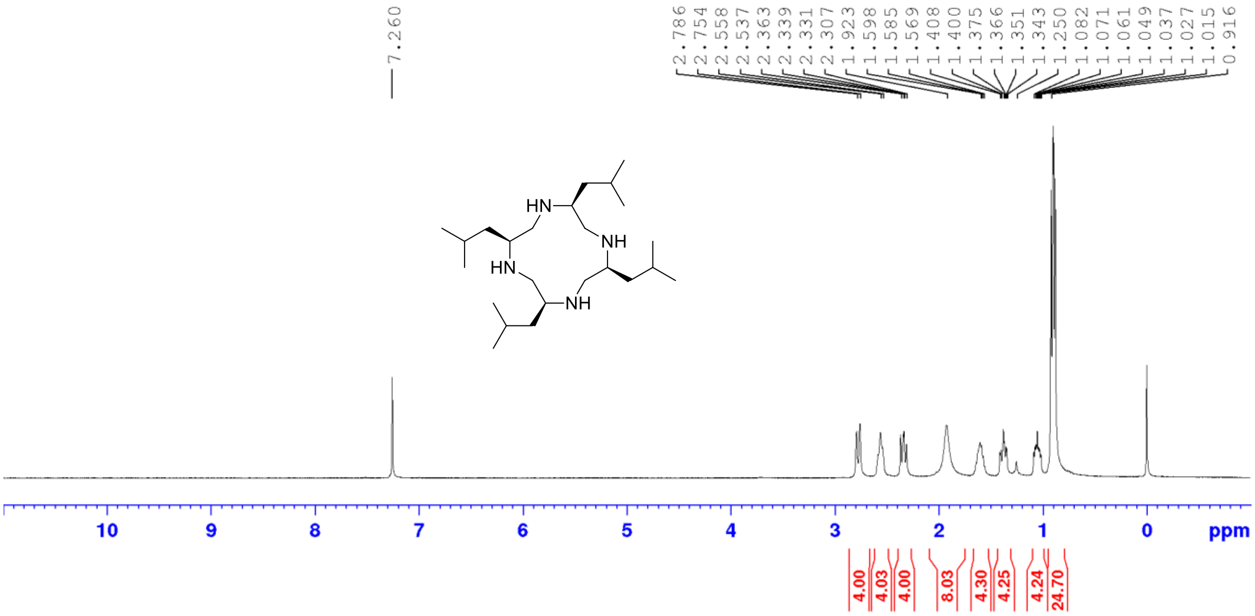

Supplementary Figure 44 | ^{1}H NMR spectrum of compound 3c.

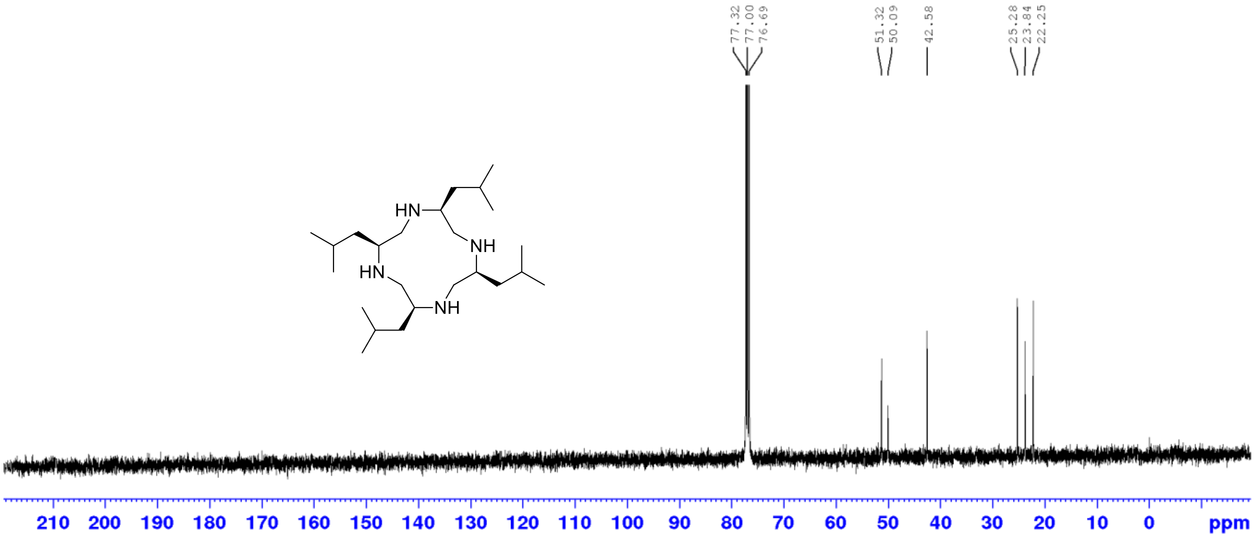

Supplementary Figure 45 | ^{13}C NMR spectrum of compound 3c.

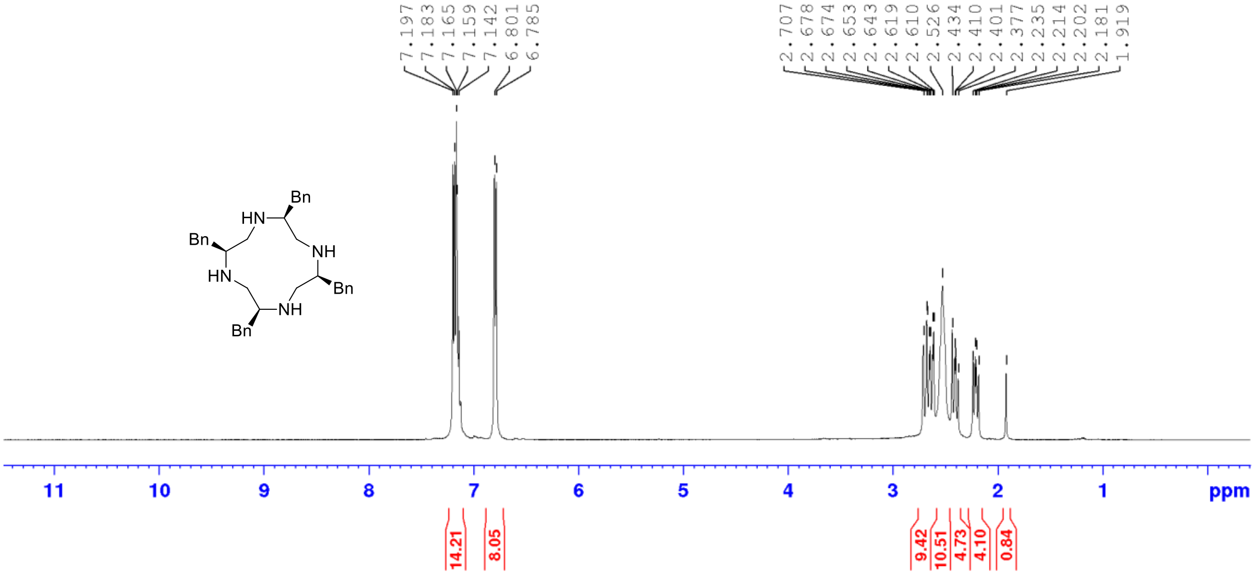

Supplementary Figure 46 | ^{1}H NMR spectrum of compound 3d.

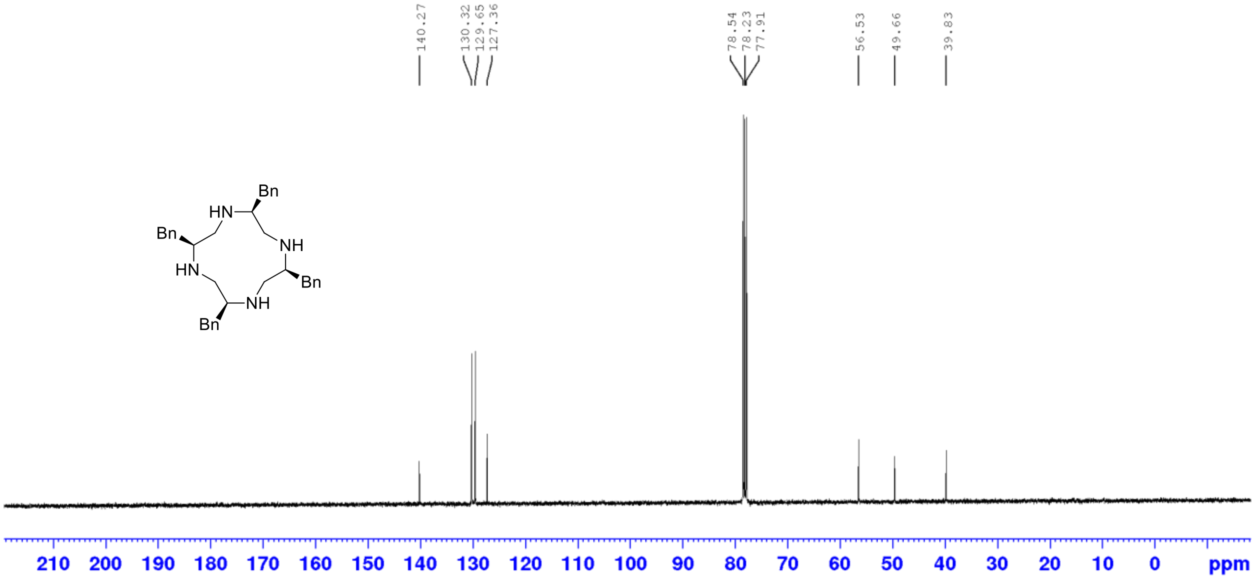

Supplementary Figure 47 | ^{13}C NMR spectrum of compound 3d.

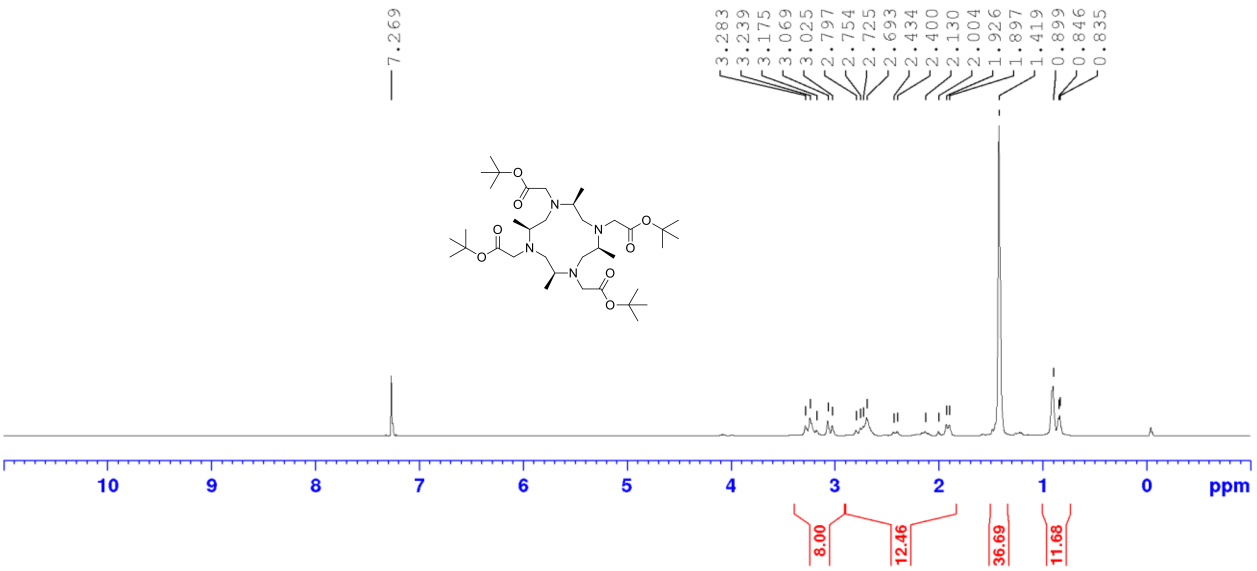

Supplementary Figure 48 | ^{1}H NMR spectrum of compound 4a.

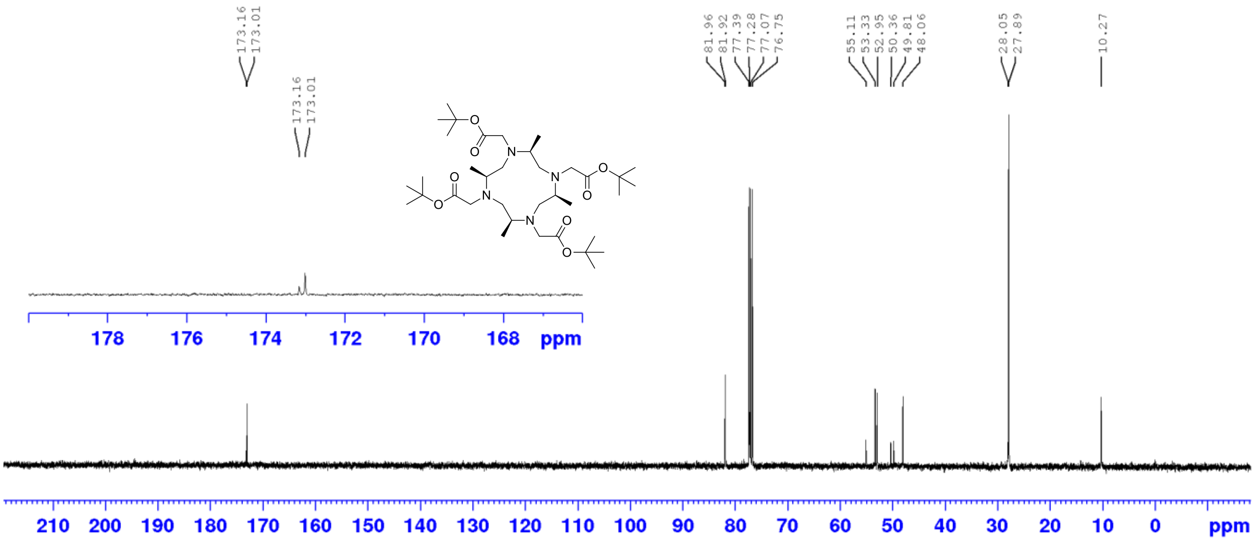

Supplementary Figure 49 | ^{13}C NMR spectrum of compound 4a.

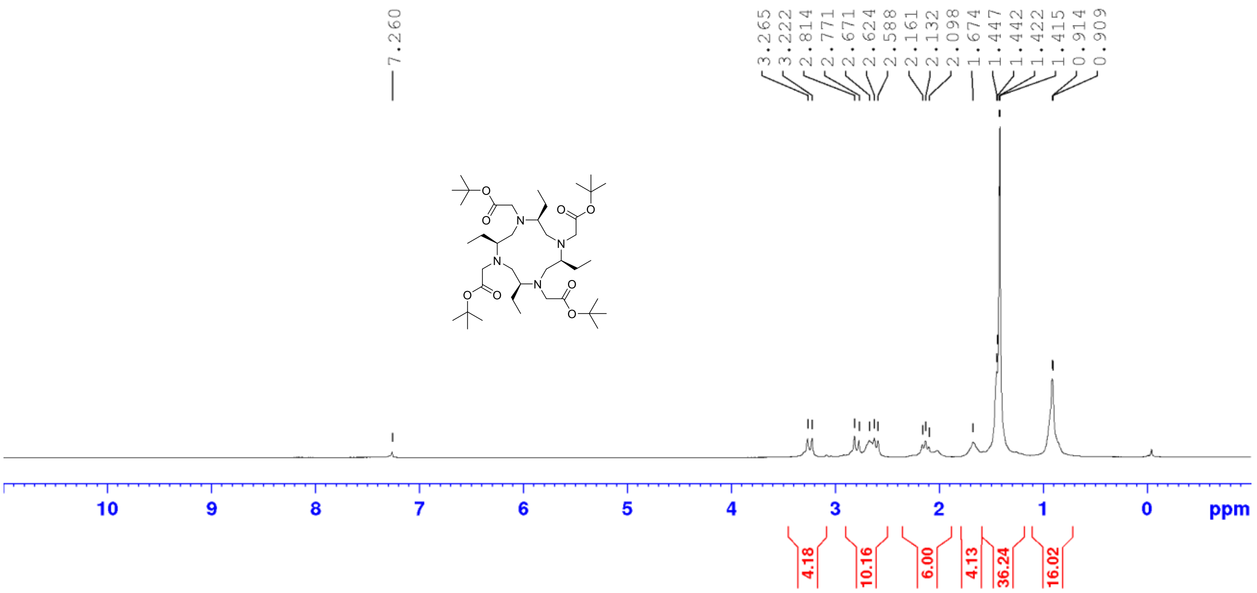

Supplementary Figure 50 | ^{1}H NMR spectrum of compound 4b.

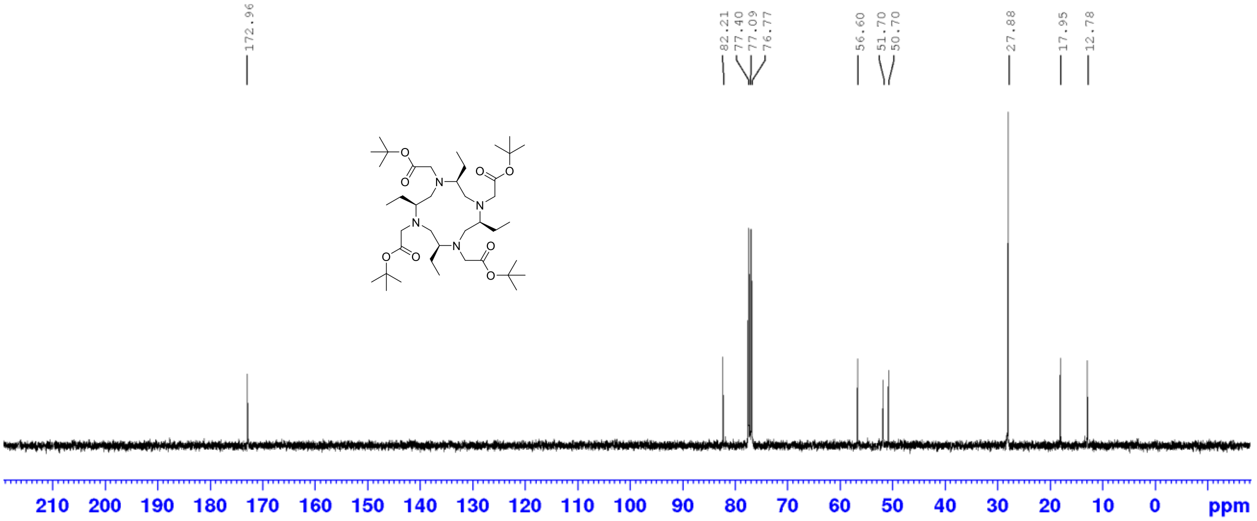

Supplementary Figure 51 | ^{13}C NMR spectrum of compound 4b.

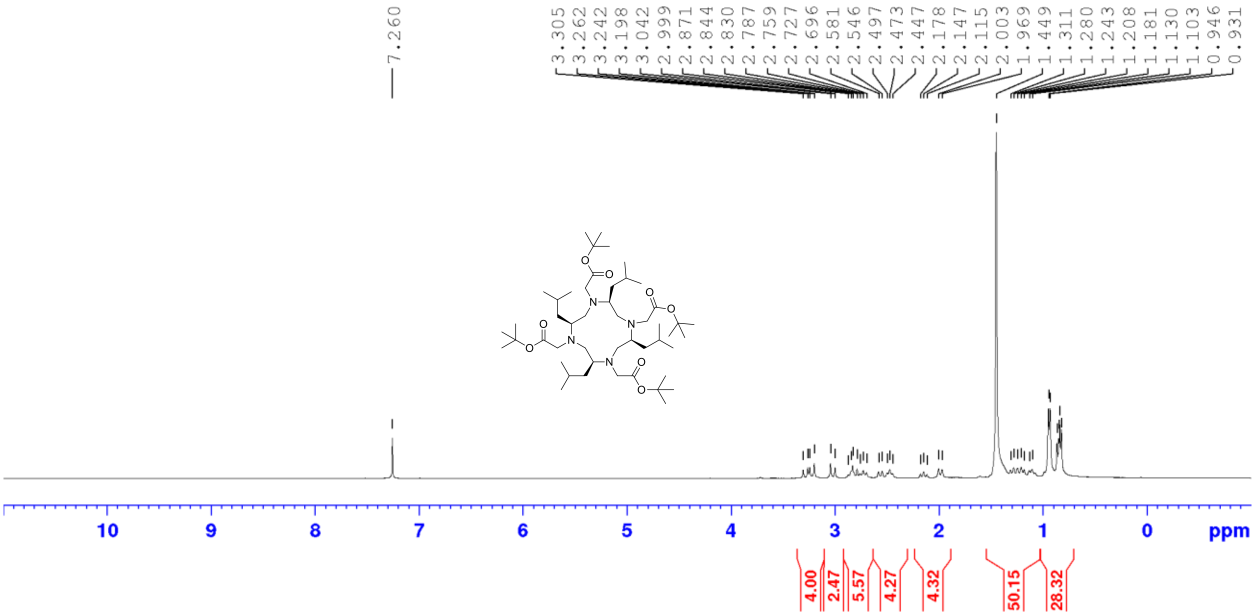

Supplementary Figure 52 | ^{1}H NMR spectrum of compound 4c.

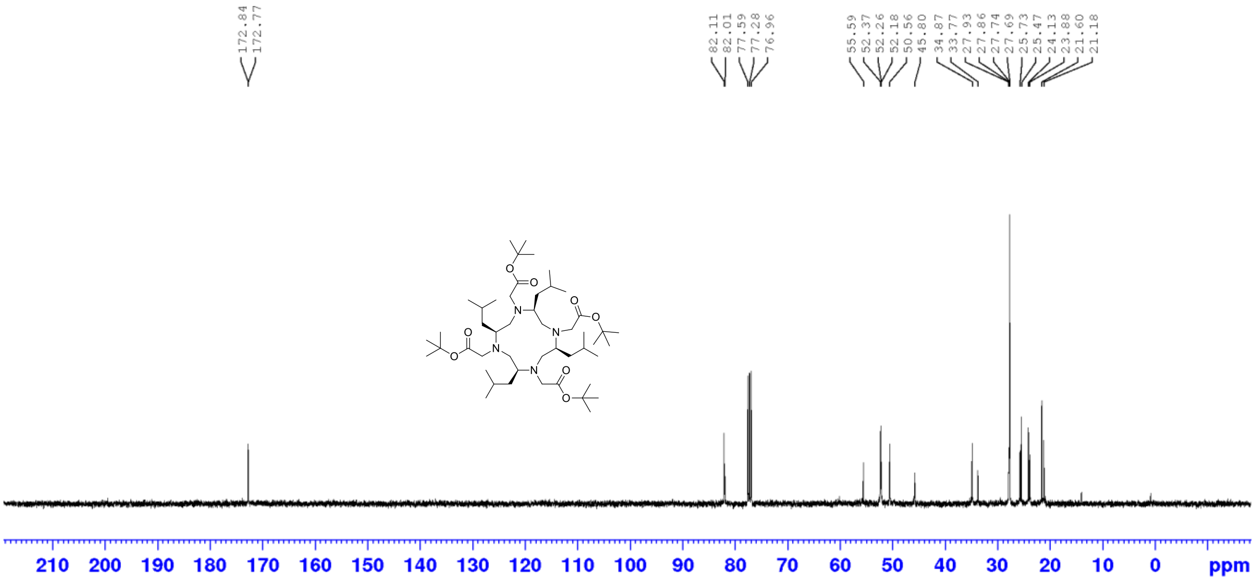

Supplementary Figure 53 | ^{13}C NMR spectrum of compound 4c.

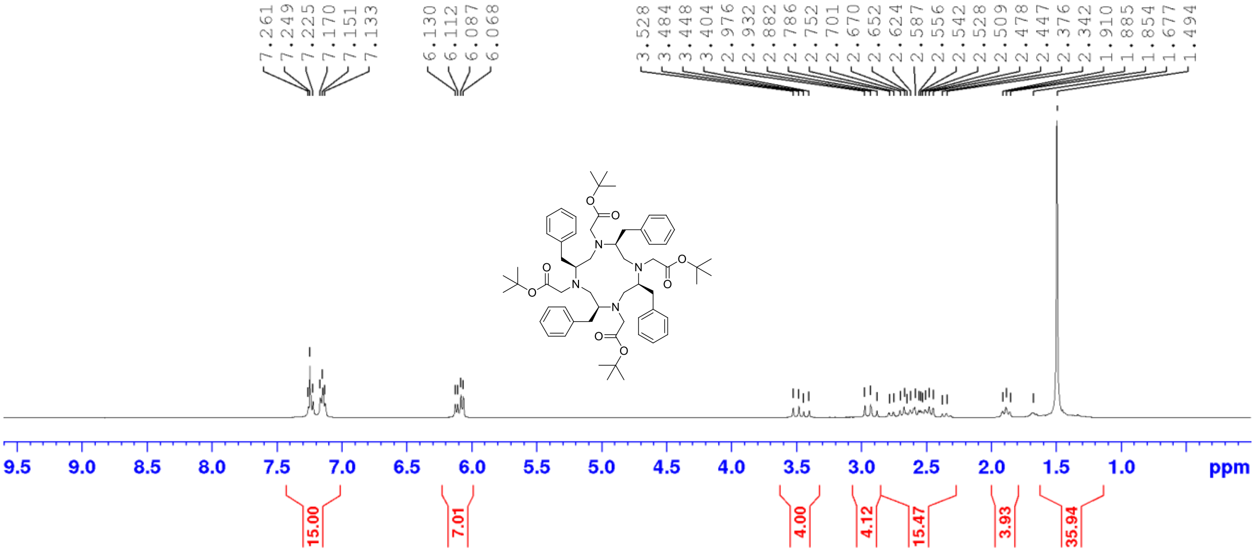

Supplementary Figure 54 | ^{1}H NMR spectrum of compound 4d.

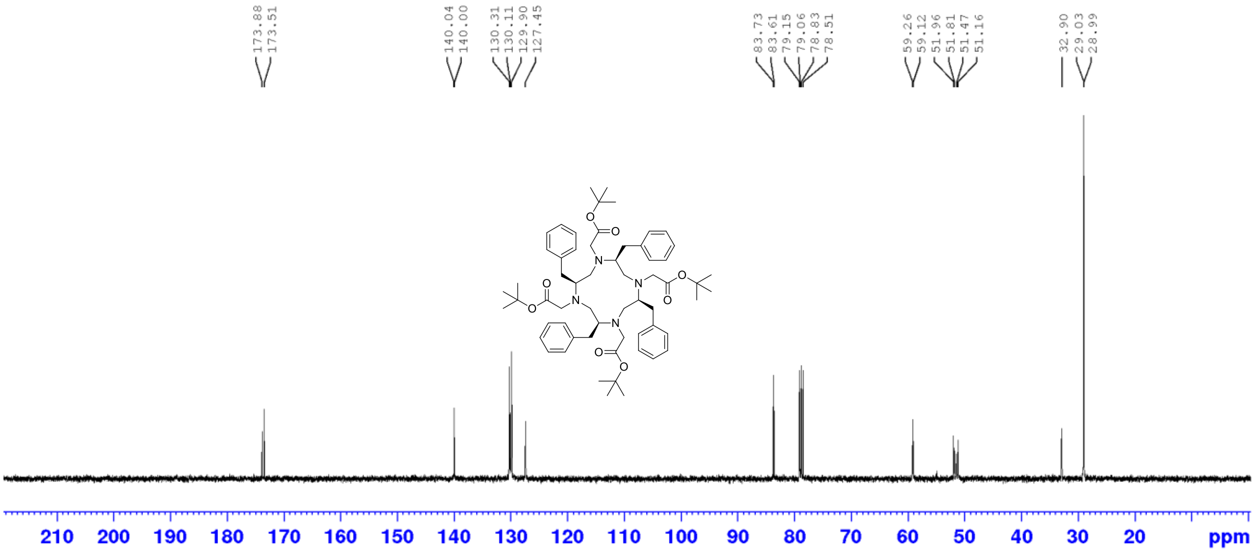

Supplementary Figure 55 | ^{13}C NMR spectrum of compound 4d.

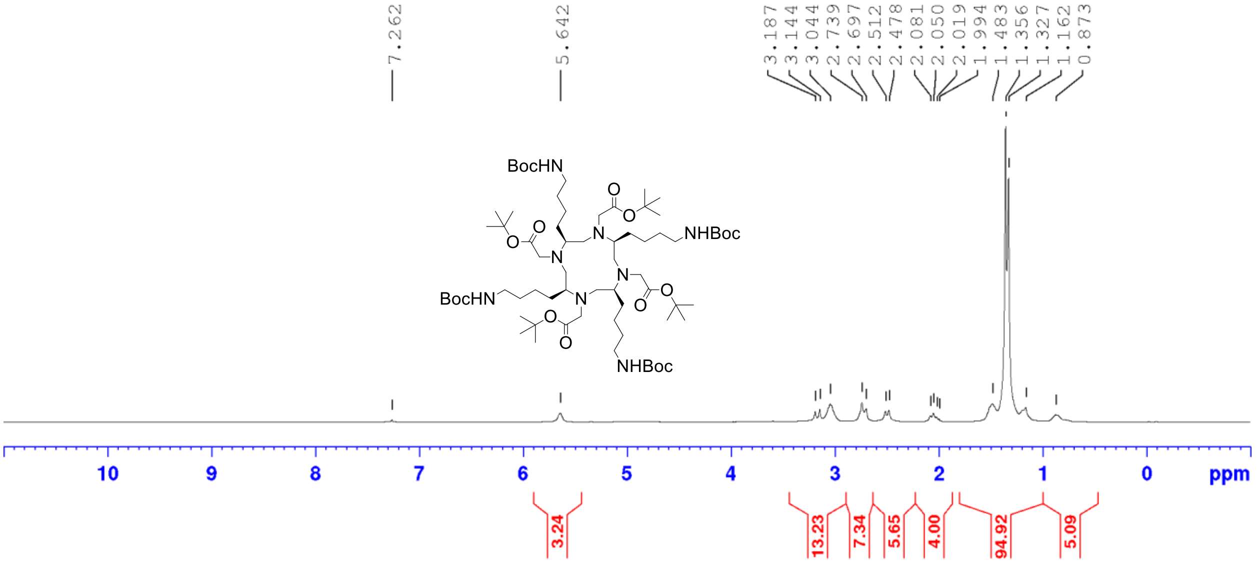

Supplementary Figure 56 | ^{1}H NMR spectrum of compound 4e.

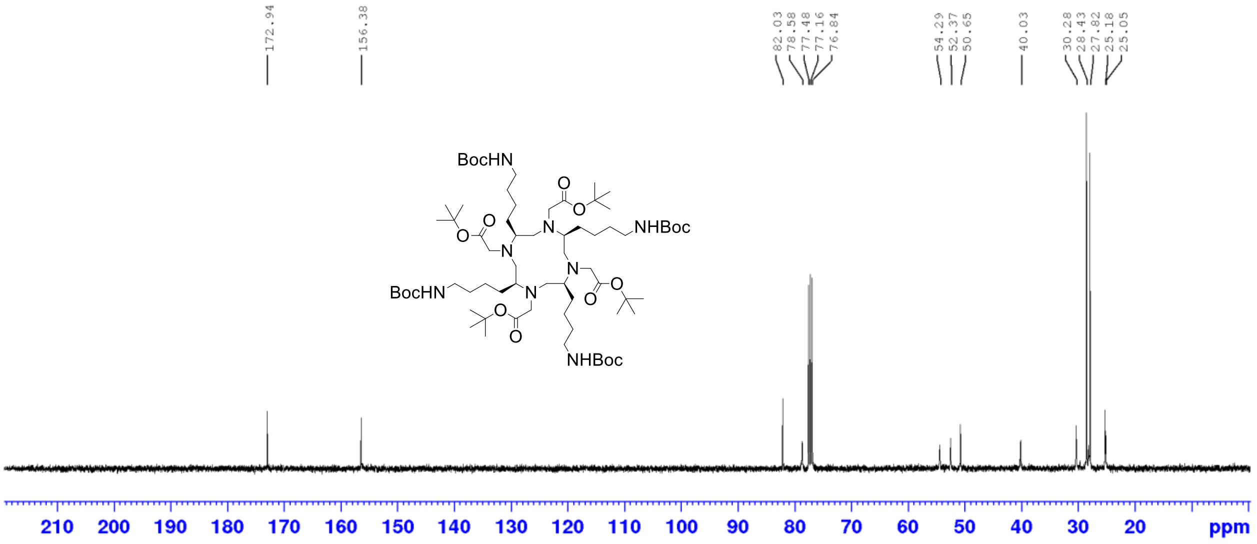

Supplementary Figure 57 | ^{13}C NMR spectrum of compound 4e.

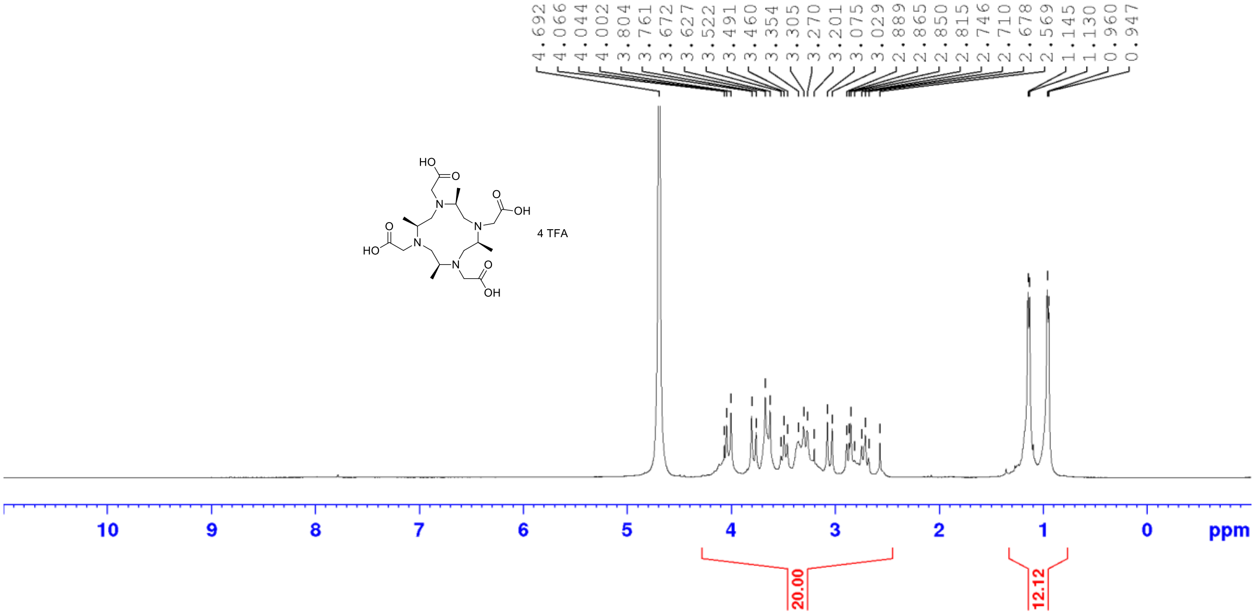

Supplementary Figure 58 | ^{1}H NMR spectrum of compound L1.

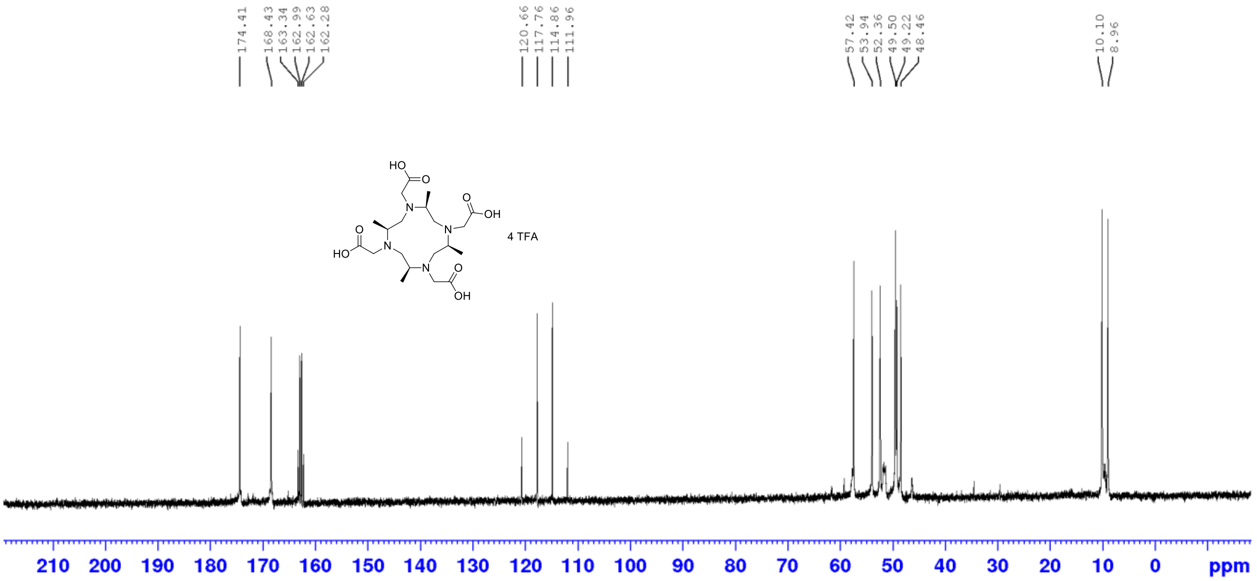

Supplementary Figure 59 | ^{13}C NMR spectrum of compound L1.

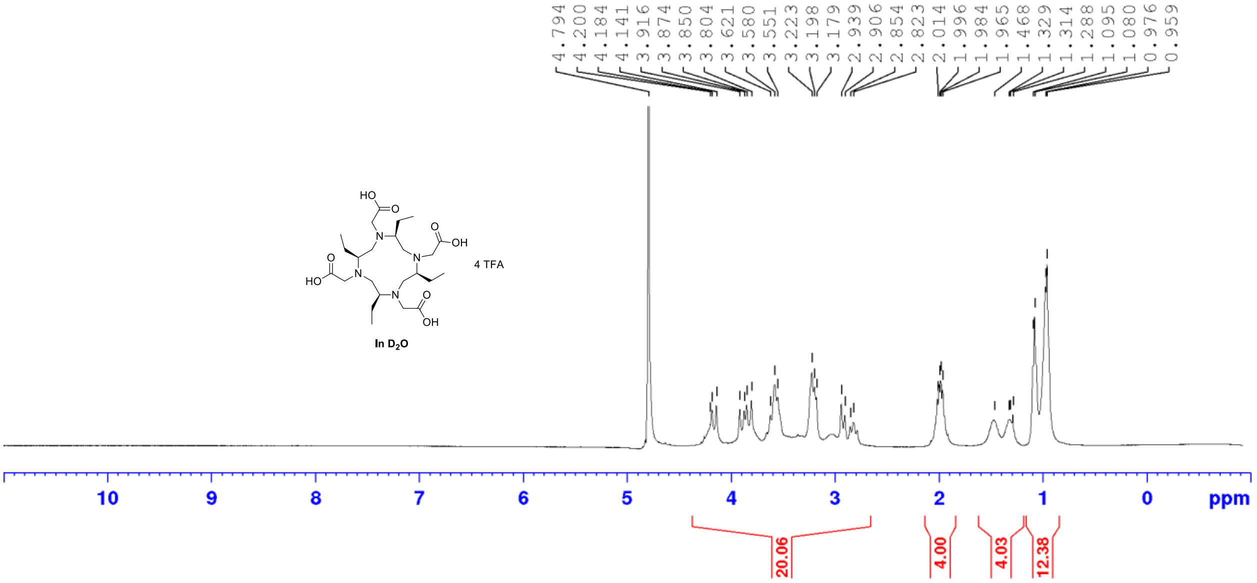

Supplementary Figure 60 | ^{1}H NMR spectrum of compound L2.

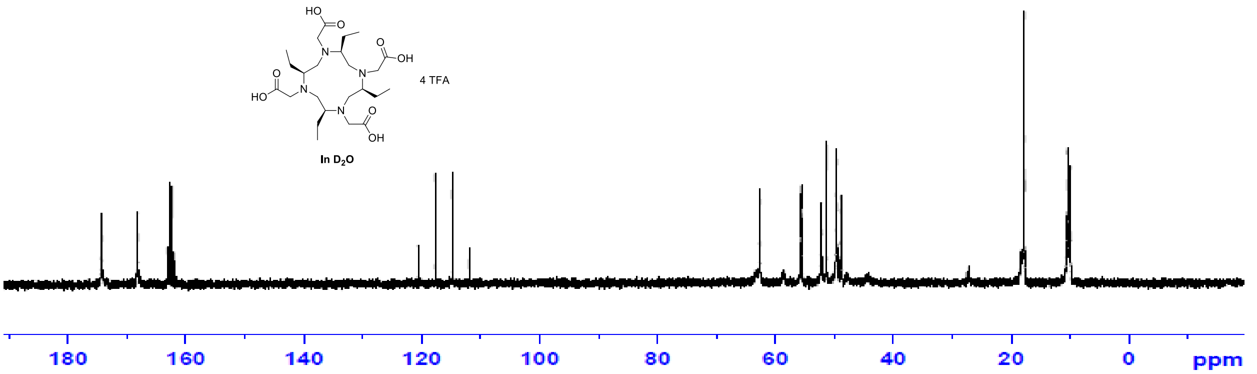

Supplementary Figure 61 | ^{13}C NMR spectrum of compound L2.

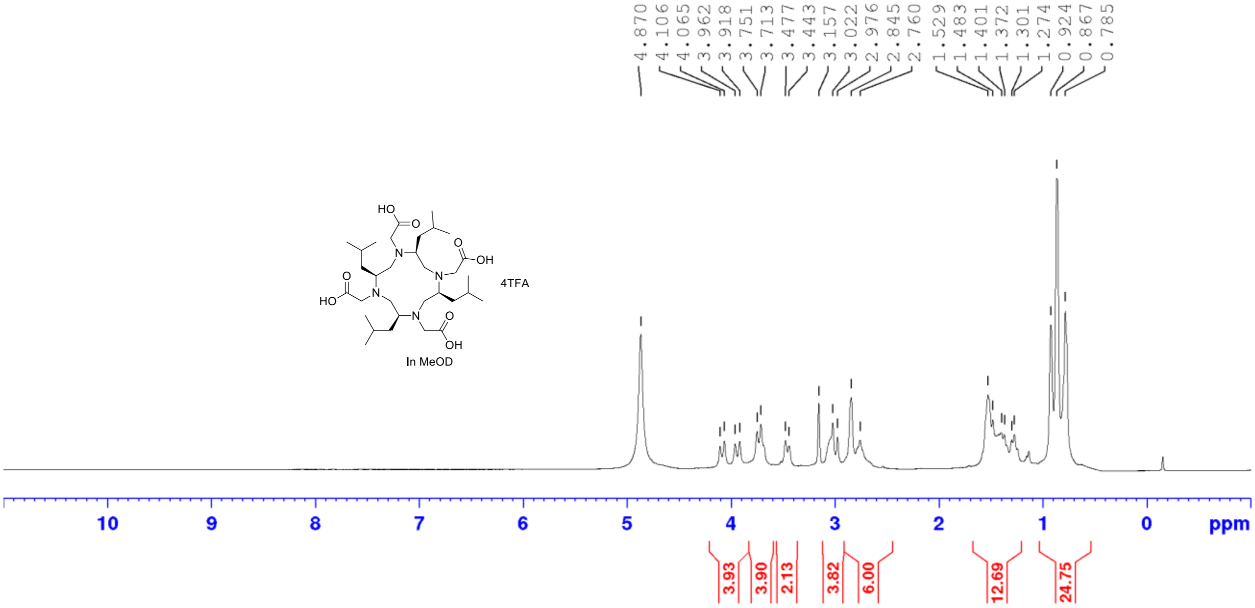

Supplementary Figure 62 | ^{1}H NMR spectrum of compound L3.

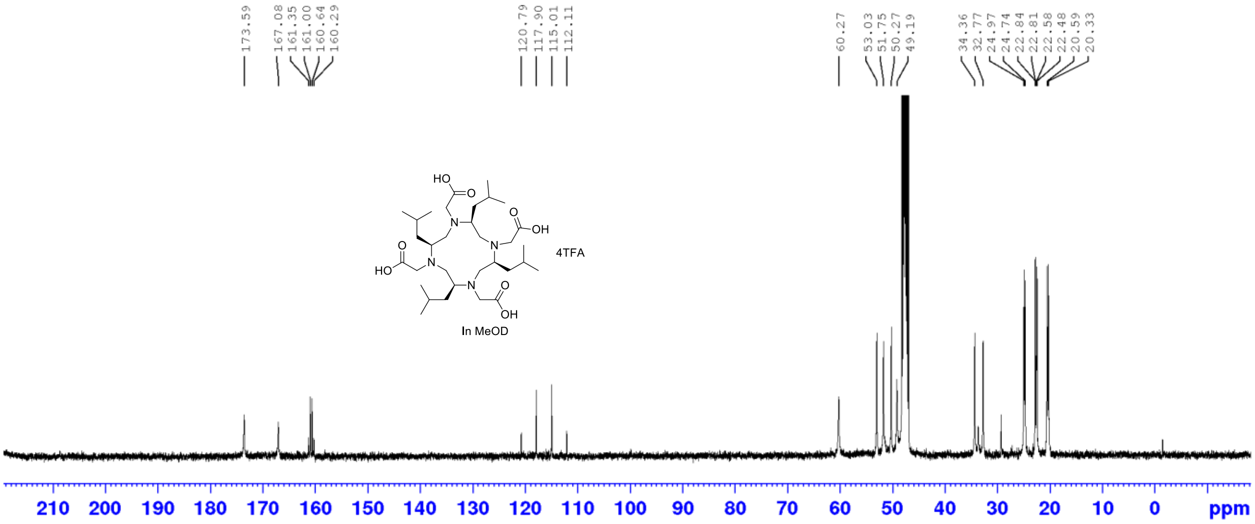

Supplementary Figure 63 | ^{13}C NMR spectrum of compound L3.

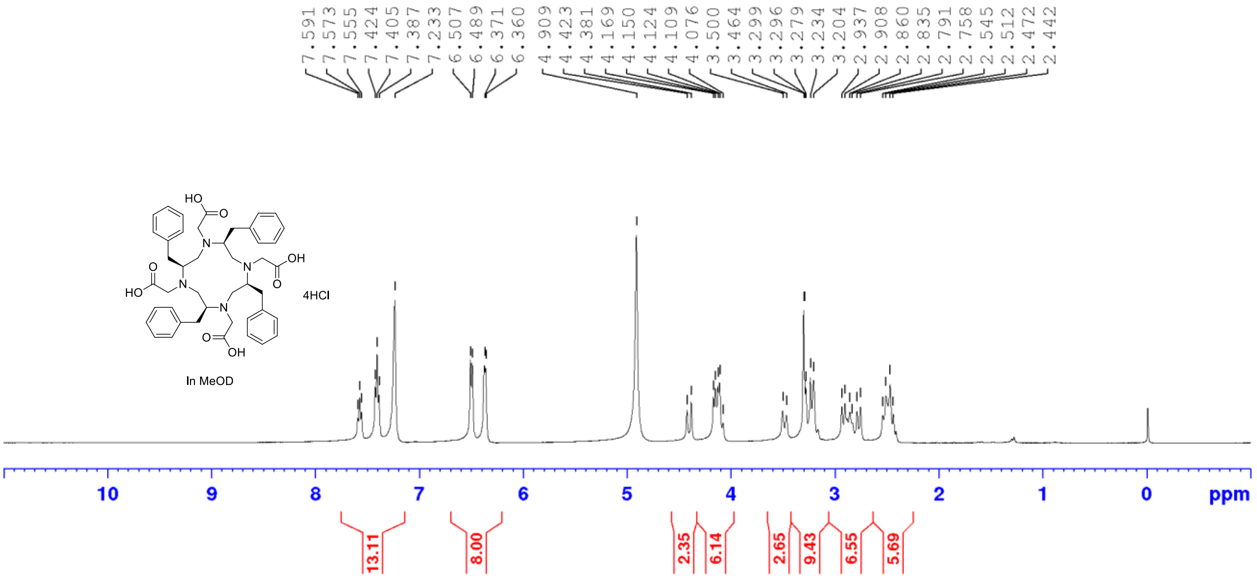

Supplementary Figure 64 | ^{1}H NMR spectrum of compound L4.

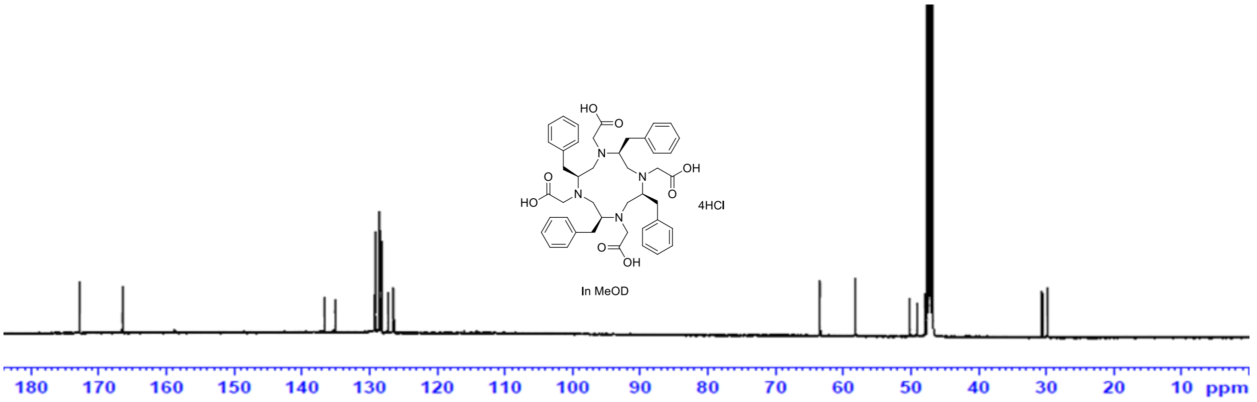

Supplementary Figure 65 | ^{13}C NMR spectrum of compound L4.

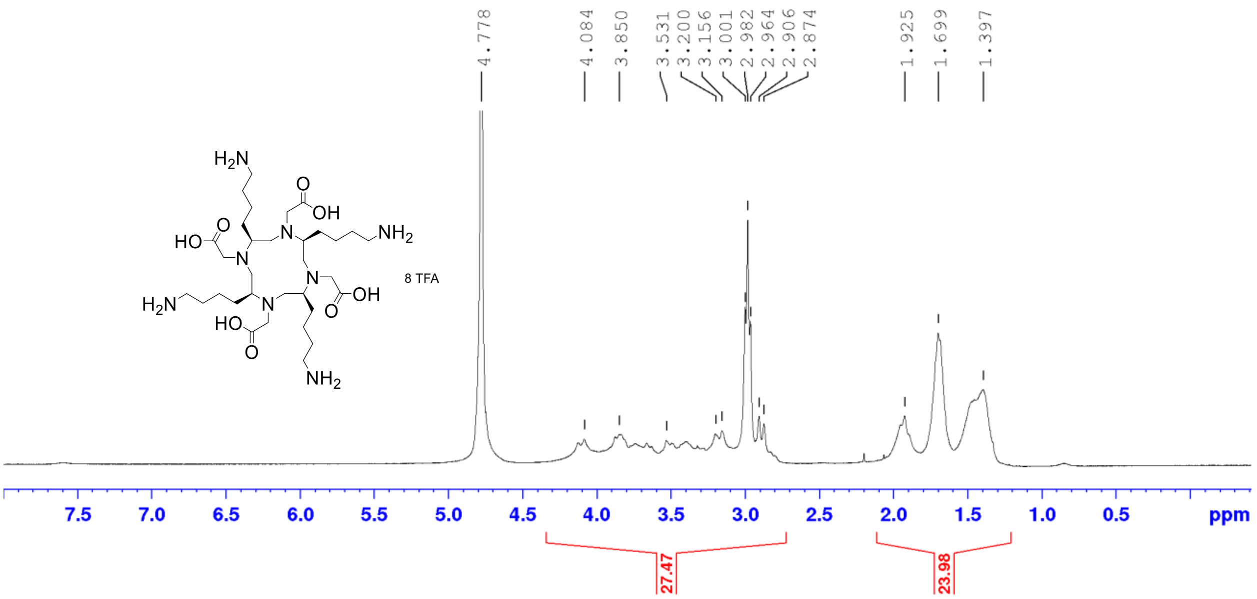

Supplementary Figure 66 | ^{1}H NMR spectrum of compound L5.

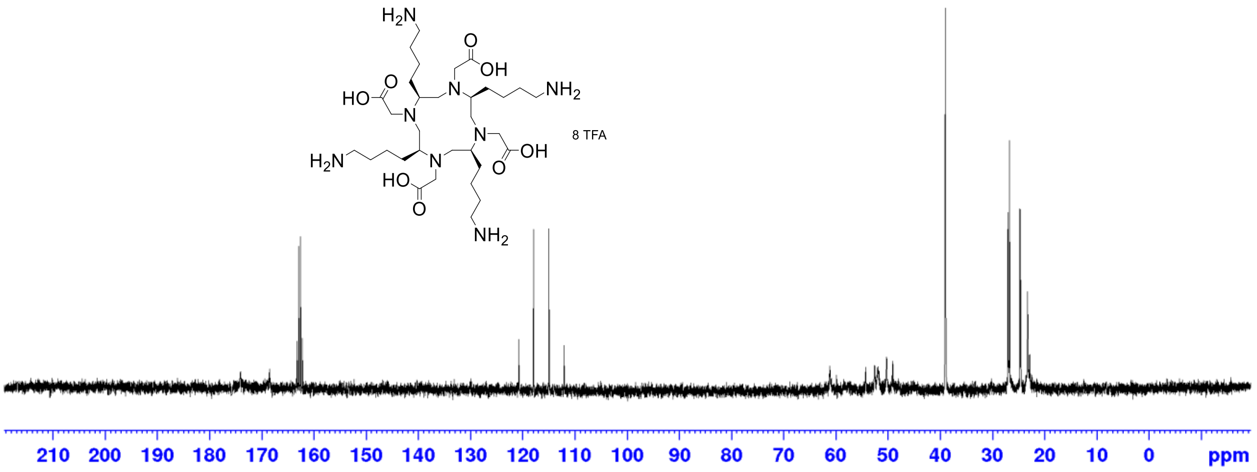

Supplementary Figure 67 | ^{13}C NMR spectrum of compound L5.

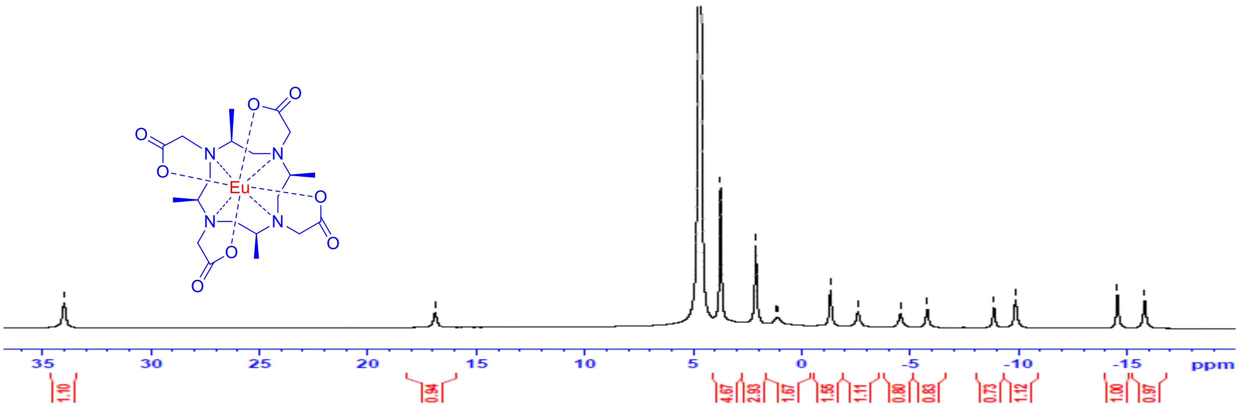

Supplementary Figure 68 | ^{1}H NMR spectrum of compound [EuL1]$^{-}$.

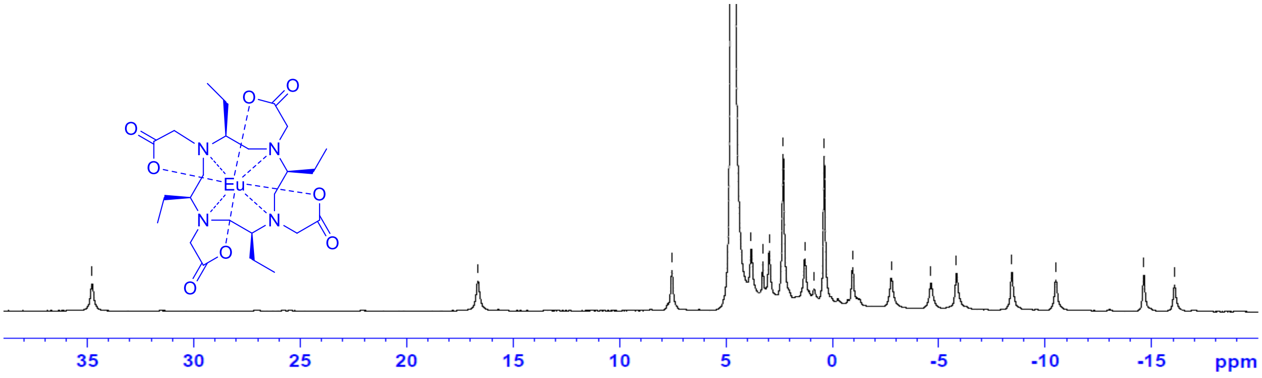

Supplementary Figure 69 | ^{1}H NMR spectrum of compound [EuL2]$^{-}$.

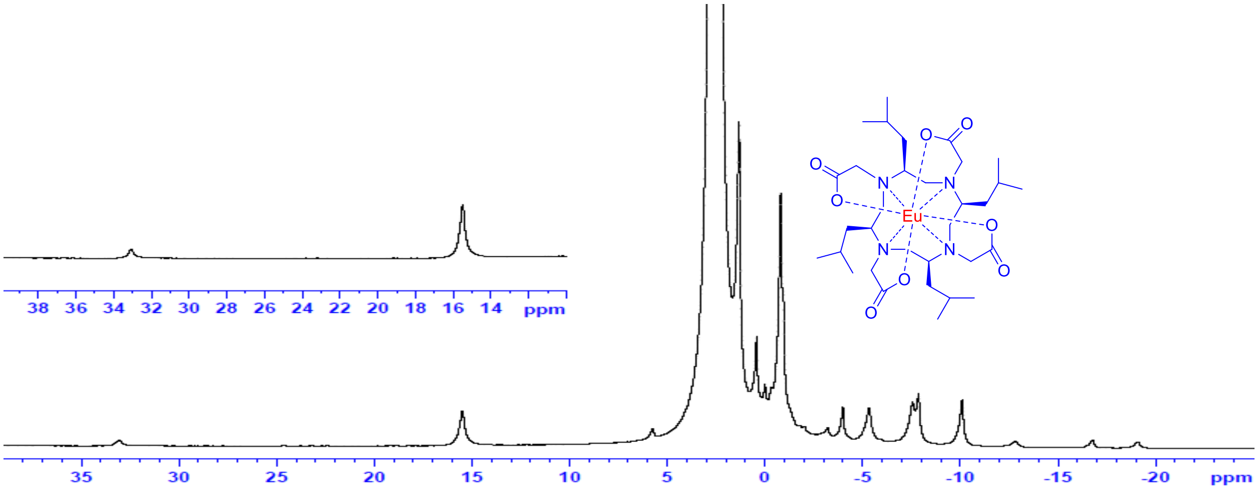

Supplementary Figure 70 | ^{1}H NMR spectrum of compound [EuL3]$^{-}$.

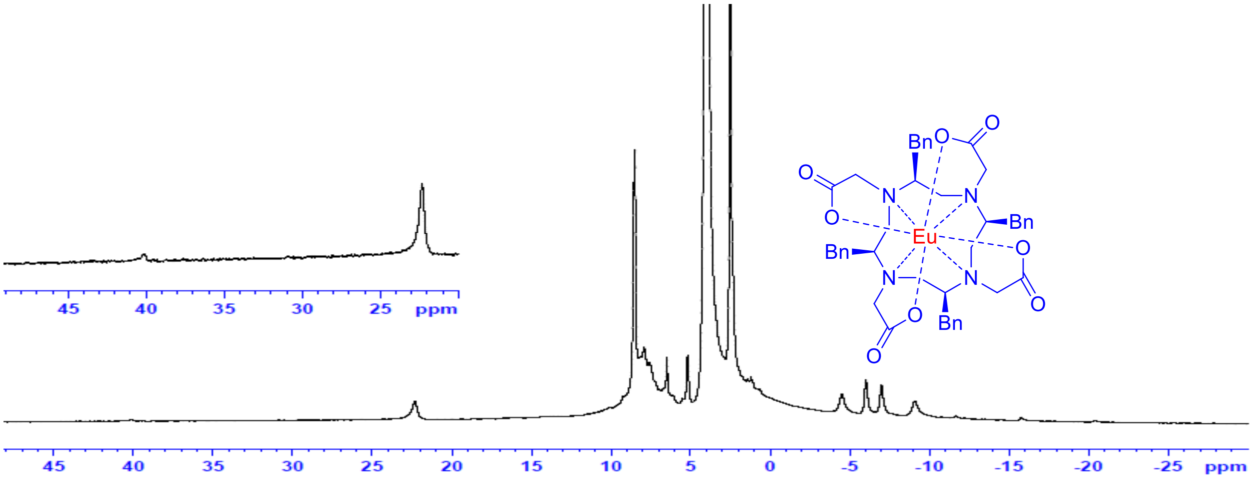

Supplementary Figure 71 | ^{1}H NMR spectrum of compound $[EuL4]^{-}$.

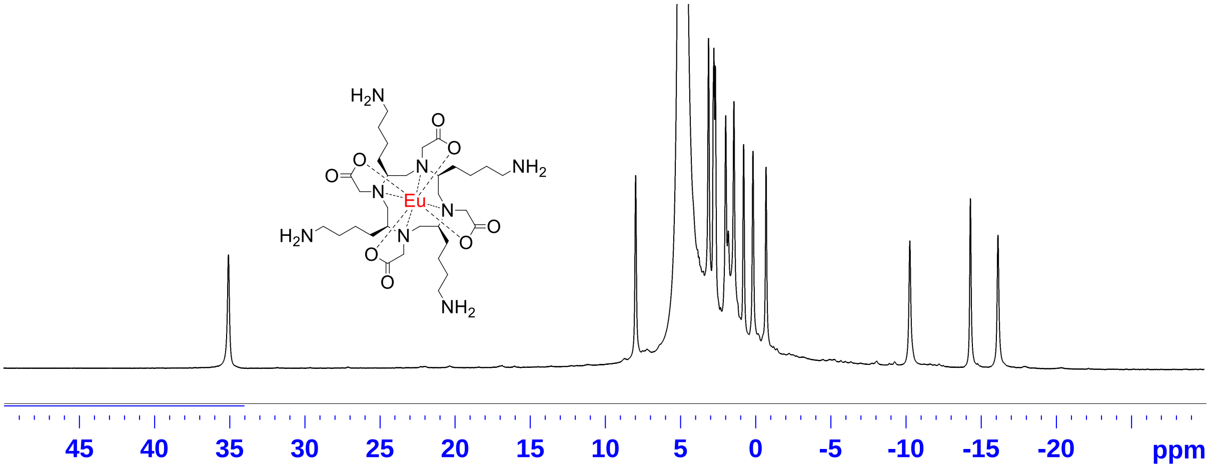

Supplementary Figure 72 | ^{1}H NMR spectrum of compound $[EuL5]^{-}$.

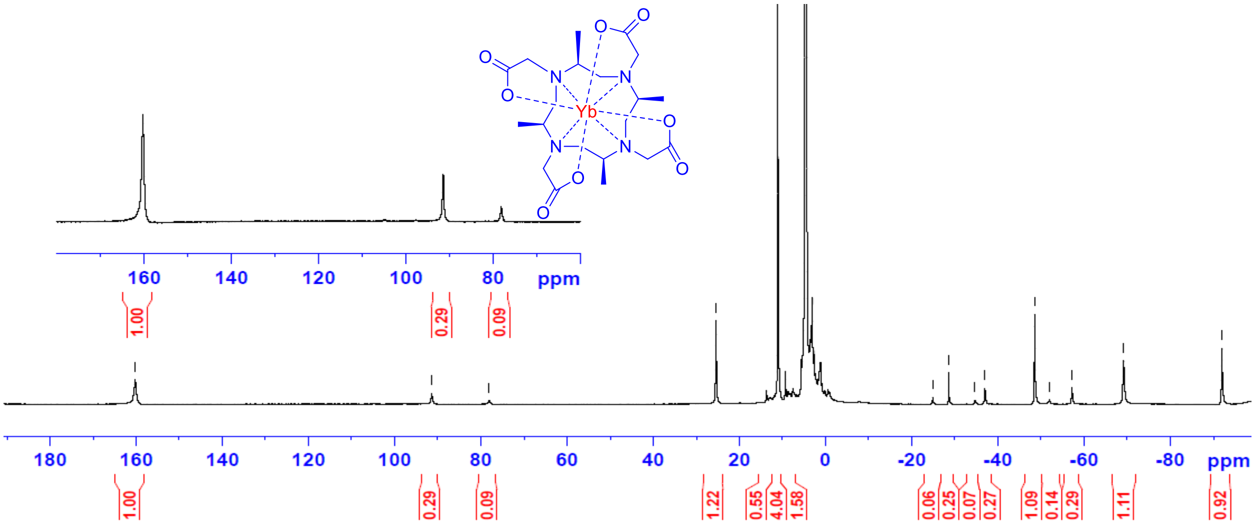

Supplementary Figure 73 | ^{1}H NMR spectrum of compound [YbL1]$^{-}$.

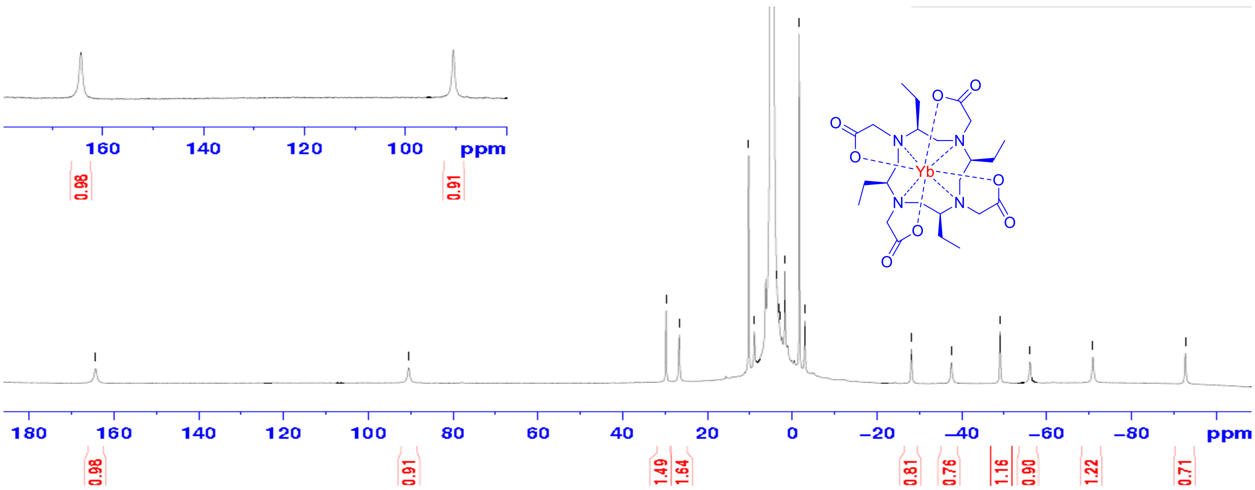

Supplementary Figure 74 | ^{1}H NMR spectrum of compound [YbL2]$^{-}$.

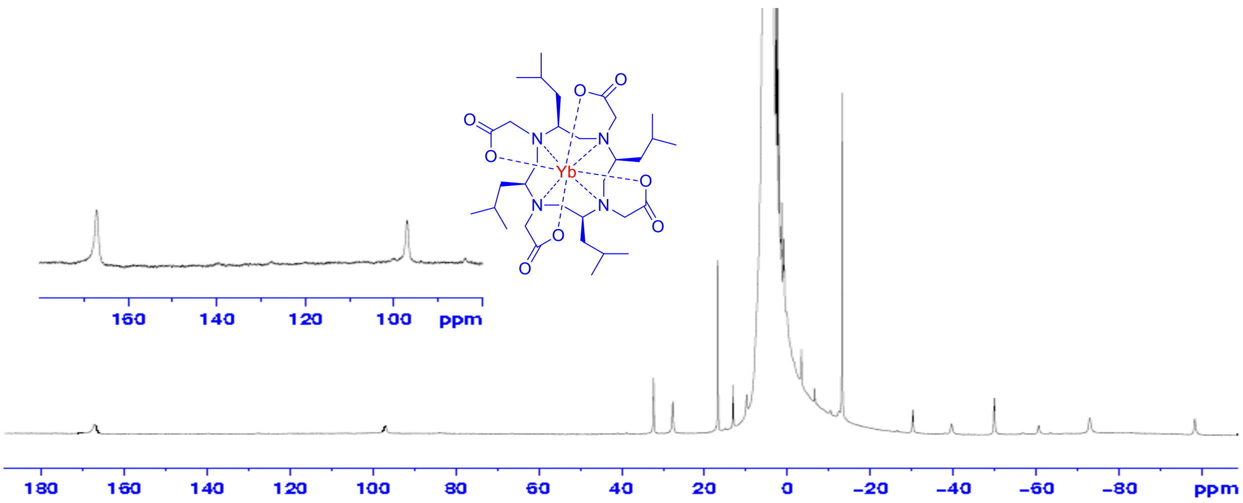

Supplementary Figure 75 | ^{1}H NMR spectrum of compound [YbL3]$^{-}$.

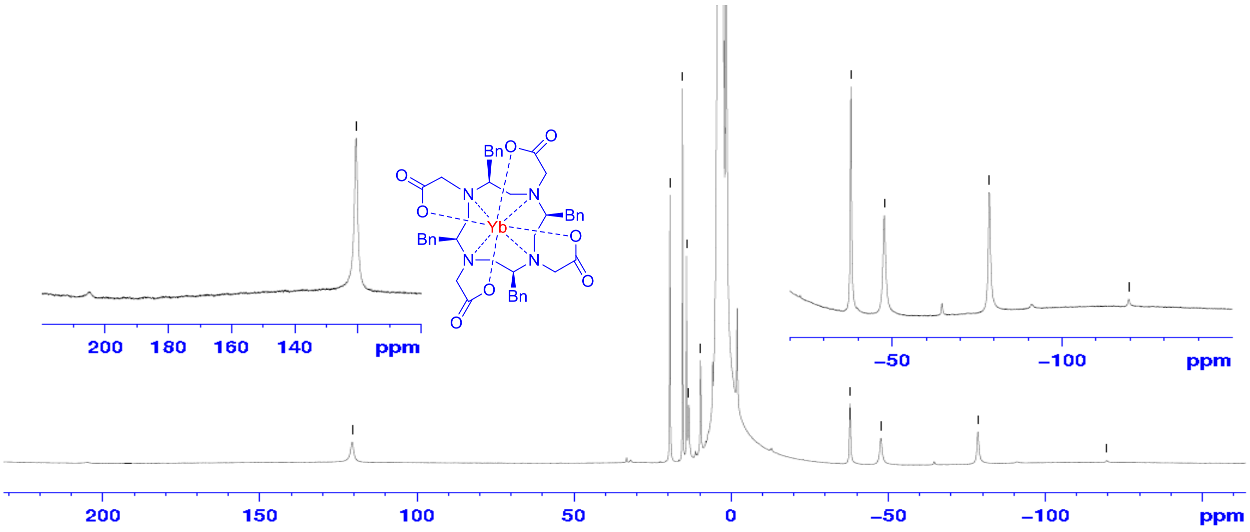

Supplementary Figure 76 | ^{1}H NMR spectrum of compound [YbL4]$^{-}$.

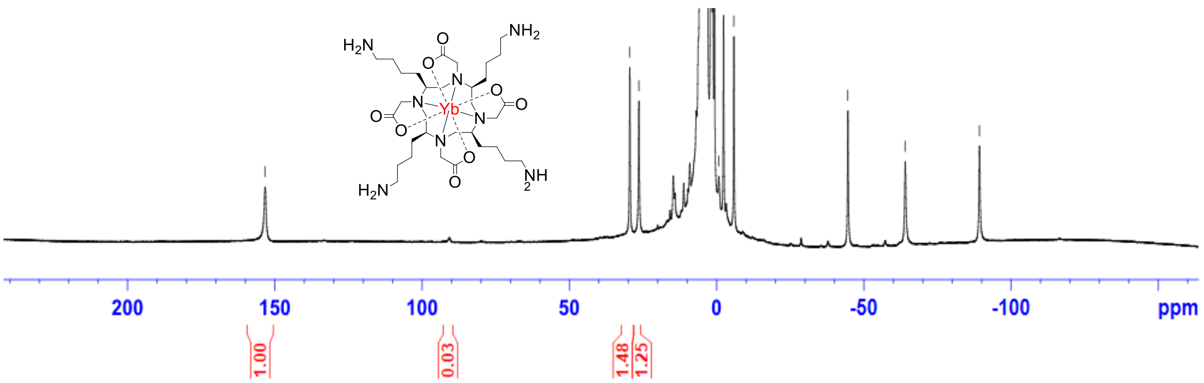

Supplementary Figure 77 | ^{1}H NMR spectrum of compound [YbL5]-.

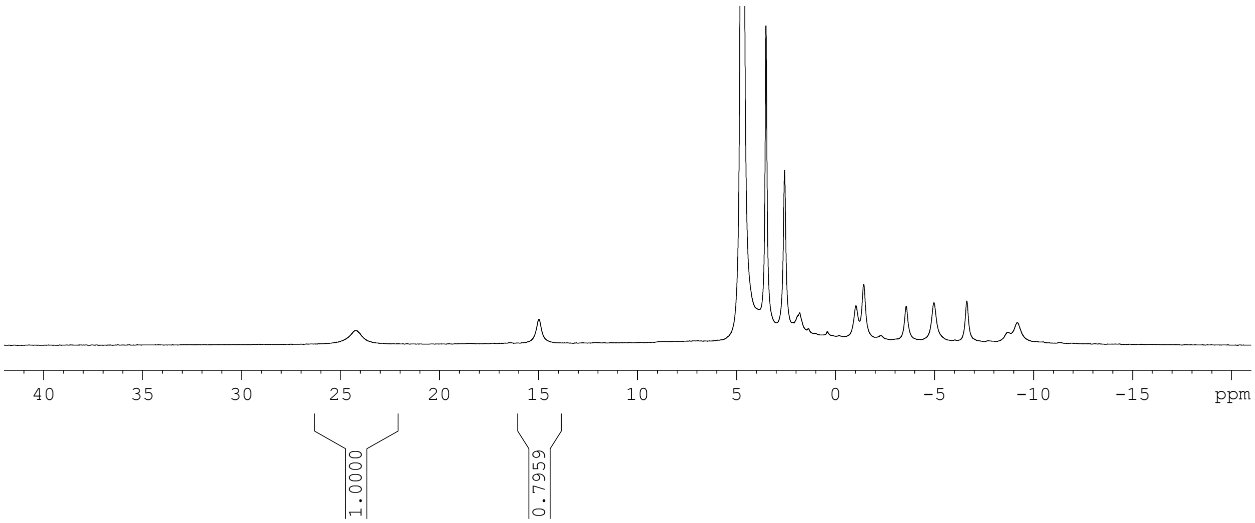

Supplementary Figure 78 | ^{1}H NMR spectrum of compound [EuL1]- at 363 K.

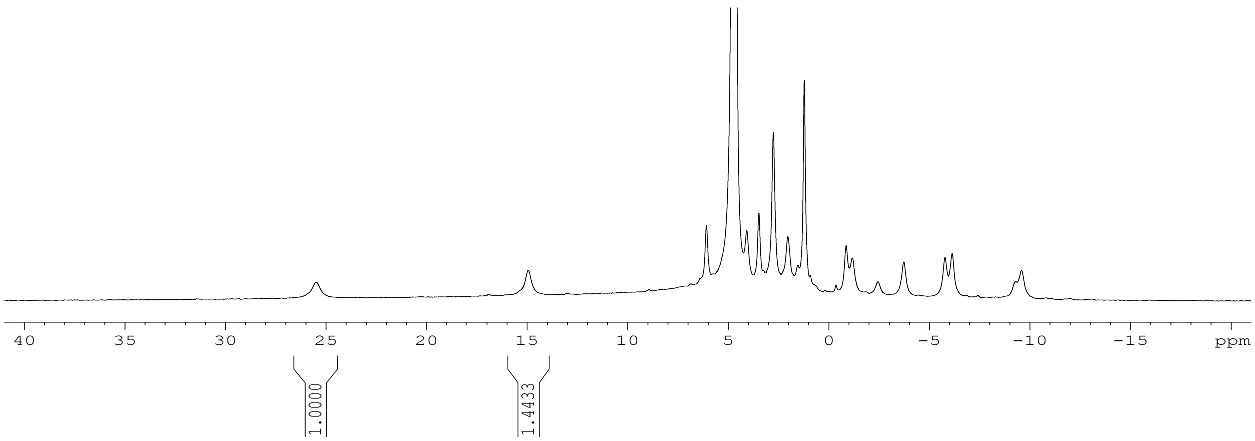

Supplementary Figure 79 | ^{1}H NMR spectrum of compound [EuL2]- at 363 K.

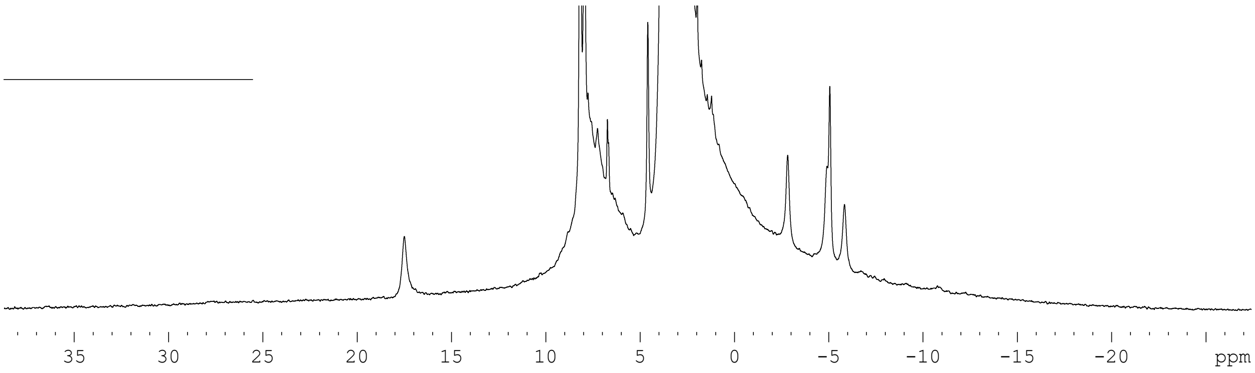

Supplementary Figure 80 | ^{1}H NMR spectrum of compound [EuL3]$^-$ at 363 K.

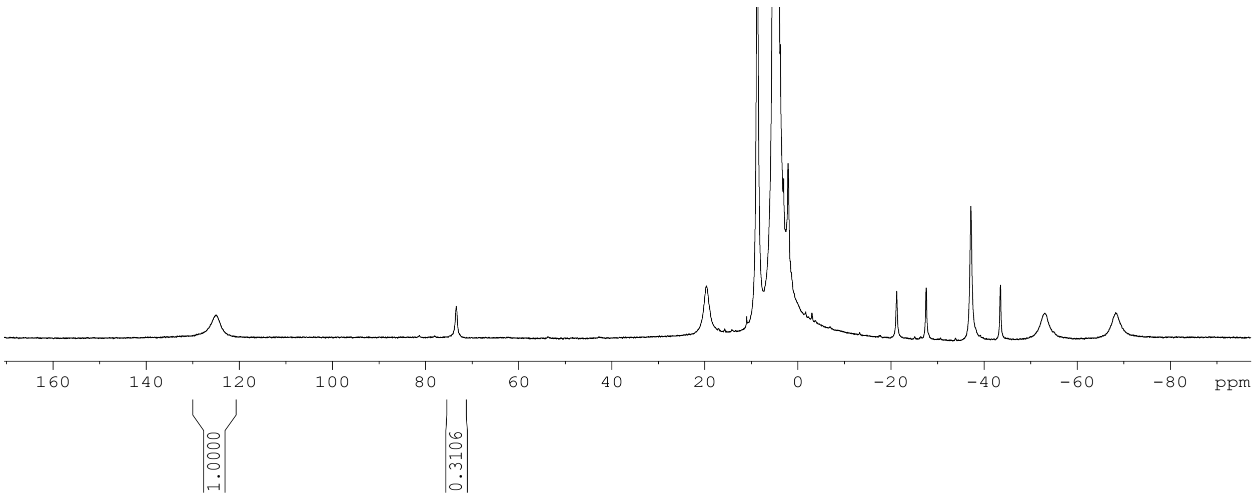

Supplementary Figure 81 | ^{1}H NMR spectrum of compound [YbL1]$^-$ at 363 K.

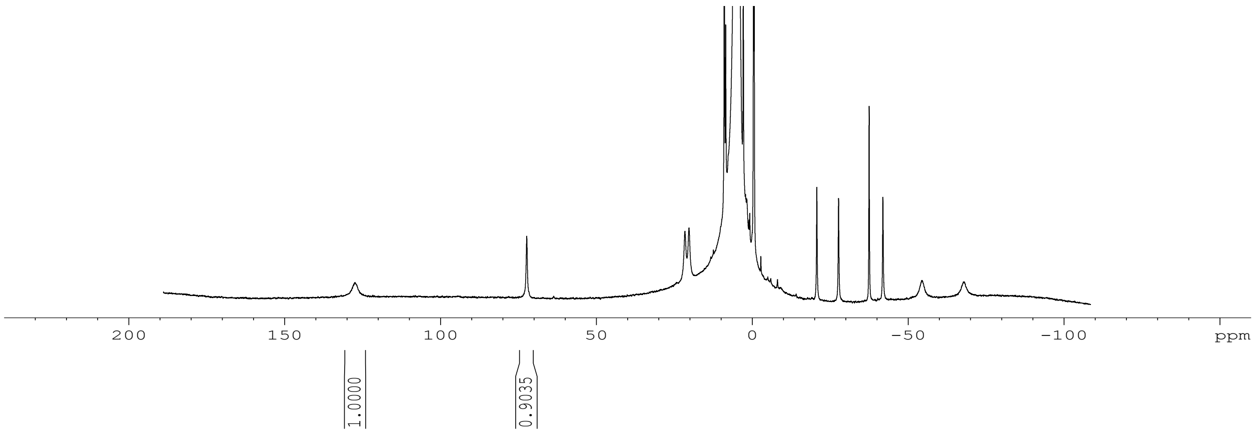

Supplementary Figure 82 | ^{1}H NMR spectrum of compound [YbL2]$^-$ at 363 K.

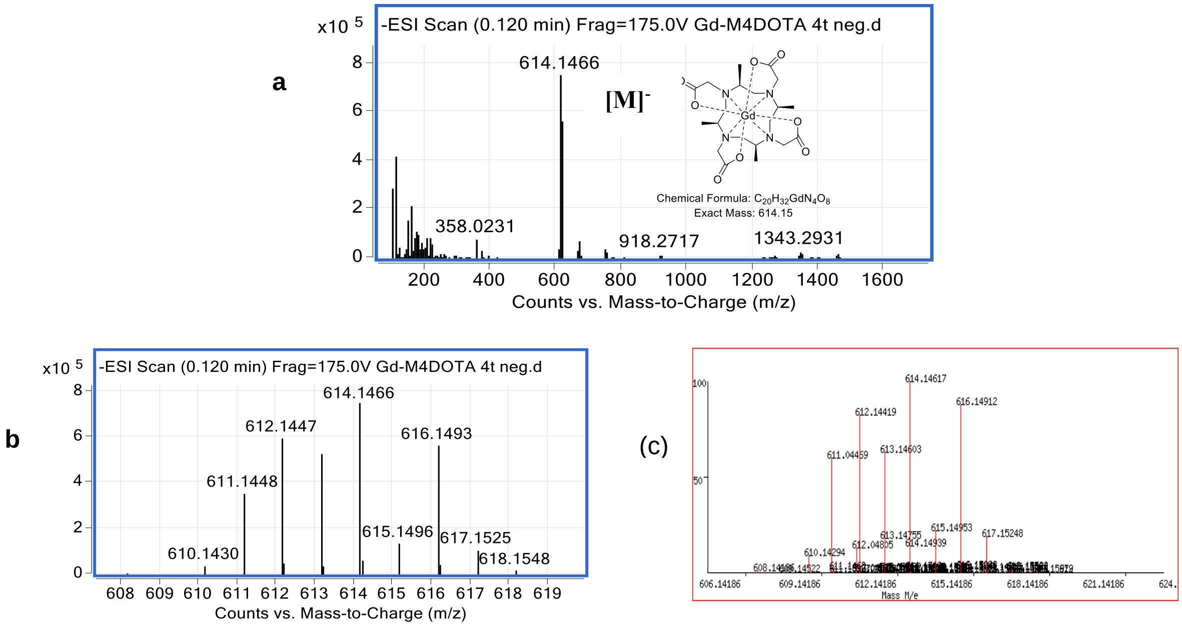

Supplementary Figure 83 | ESI-MS of [GdL1]⁻. a Full range of the MS in negative pattern; **b** Zoom in spectrum; **c** Calculated isotope distribution of the mass spectrum which was calculated using the online system of Scientific Instrument Services, INC. (SIS).

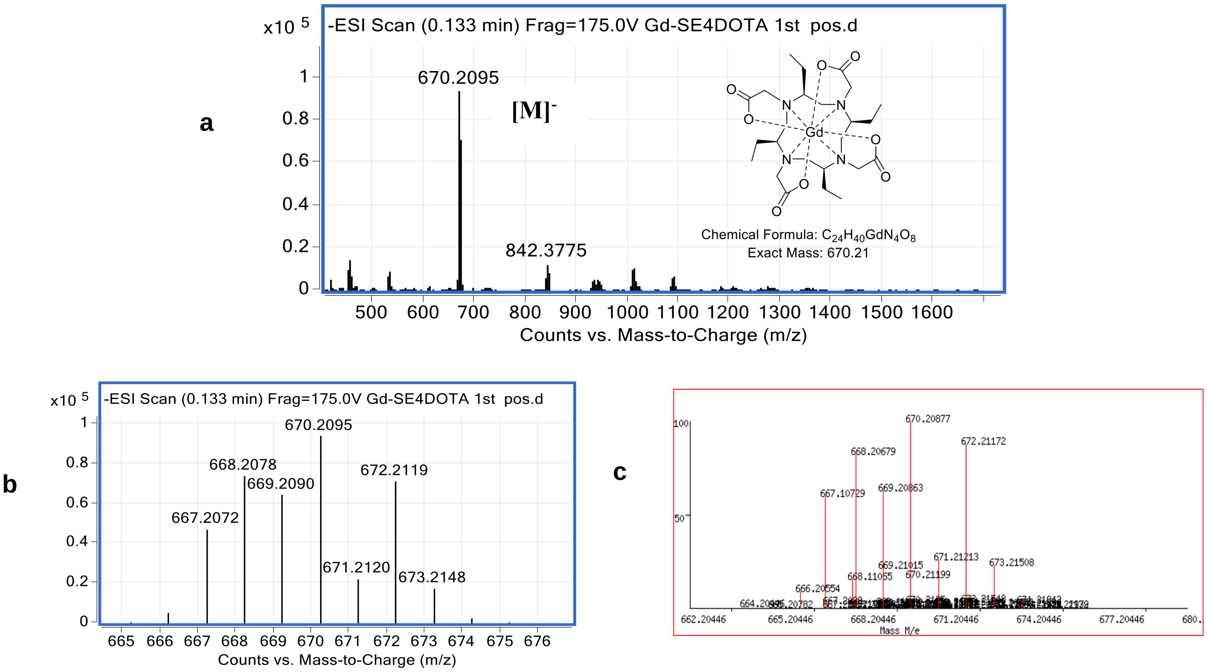

Supplementary Figure 84 | ESI-MS of [GdL2A]⁻. a Full range of the MS in negative pattern; **b** Zoom in spectrum; **c** Calculated isotope distribution of the mass spectrum.

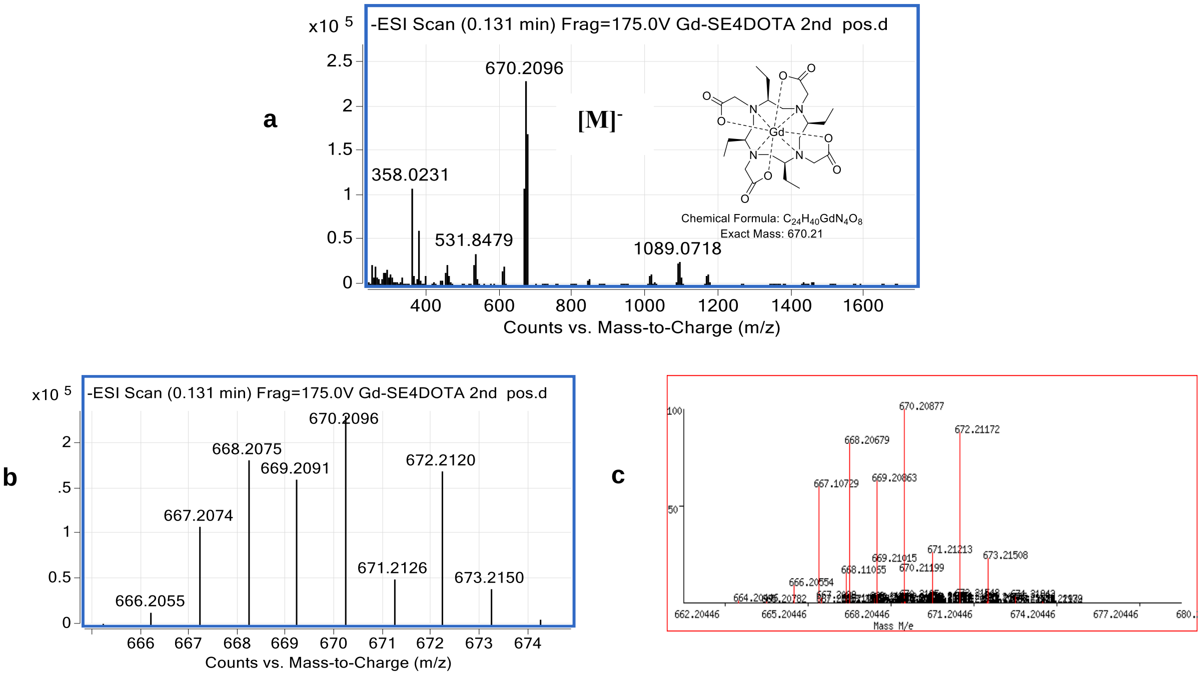

Supplementary Figure 85 | ESI-MS of [GdL2B]$^-$. a Full range of the MS in negative pattern; **b** Zoom in spectrum; **c** Calculated isotope distribution of the mass spectrum.

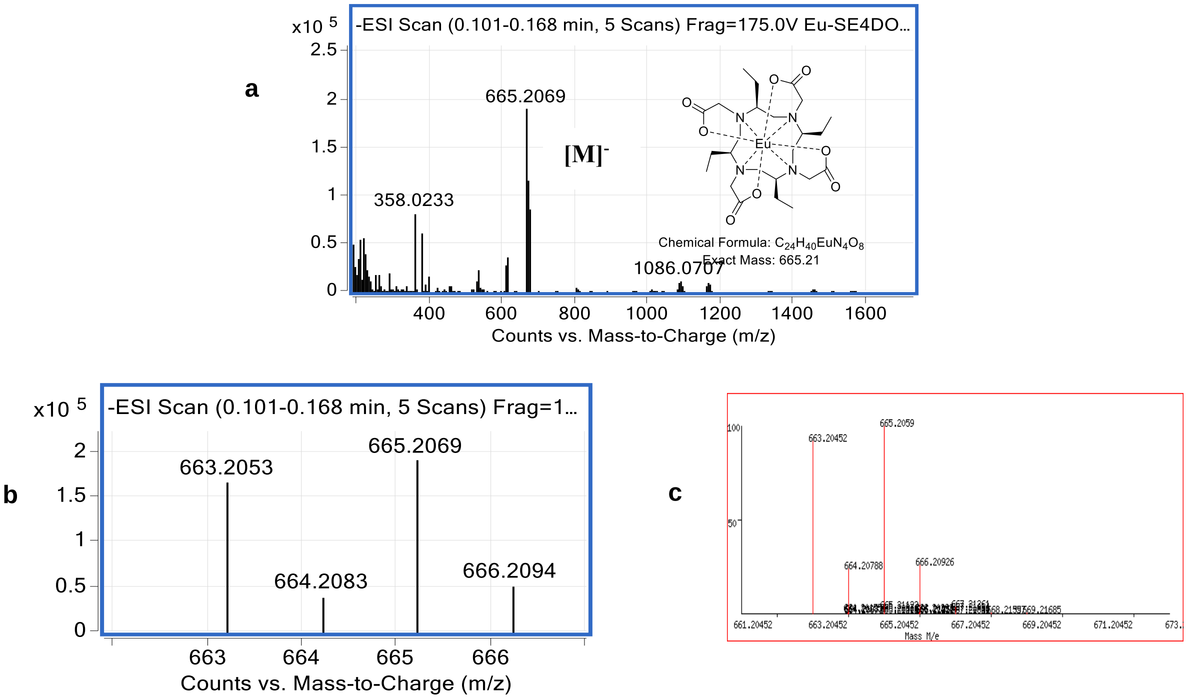

Supplementary Figure 86 | ESI-MS of [EuL2A]$^-$: a Full range of the MS in negative pattern; **b** Zoom in spectrum; **c** Calculated isotope distribution of the mass spectrum.

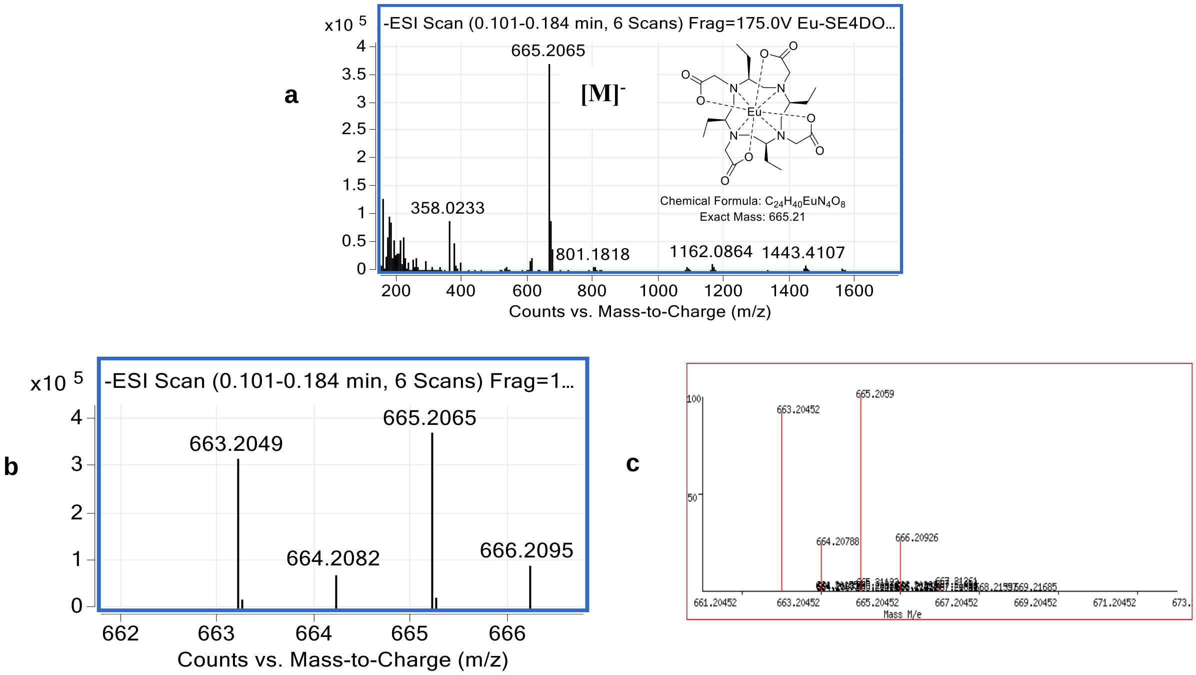

Supplementary Figure 87 | ESI-MS of [EuL2B]$^-$: a Full range of the MS in negative pattern; **b** Zoom in spectrum; **c** Calculated isotope distribution of the mass spectrum.

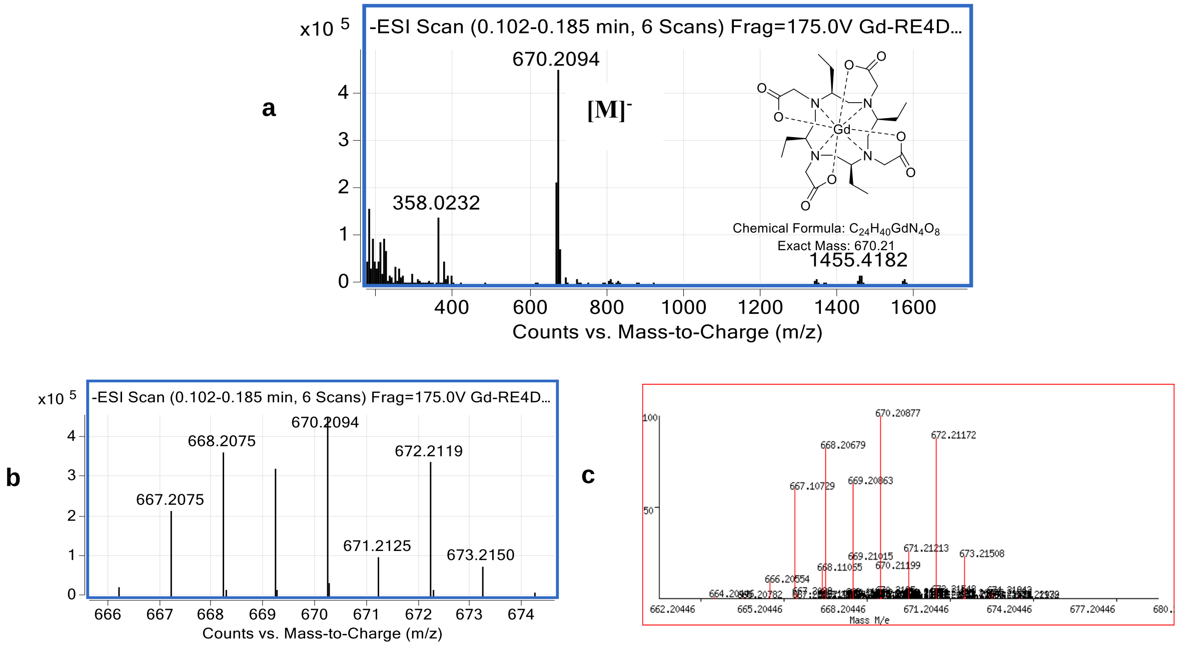

Supplementary Figure 88 | ESI-MS of [Gd(R)L2A]$^-$: a Full range of the MS in negative pattern; **b** Zoom in spectrum; **c** Calculated isotope distribution of the mass spectrum.

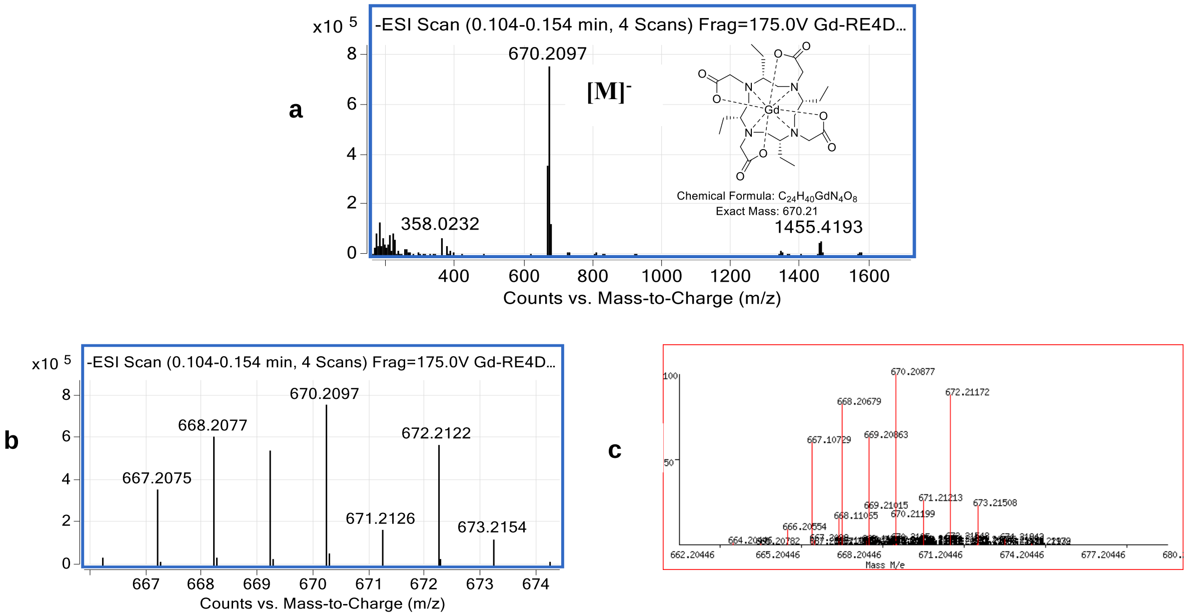

Supplementary Figure 89 | ESI-MS of [Gd(R)L2B]$^-$: a Full range of the MS in negative pattern; **b** Zoom in spectrum; **c** Calculated isotope distribution of the mass spectrum.

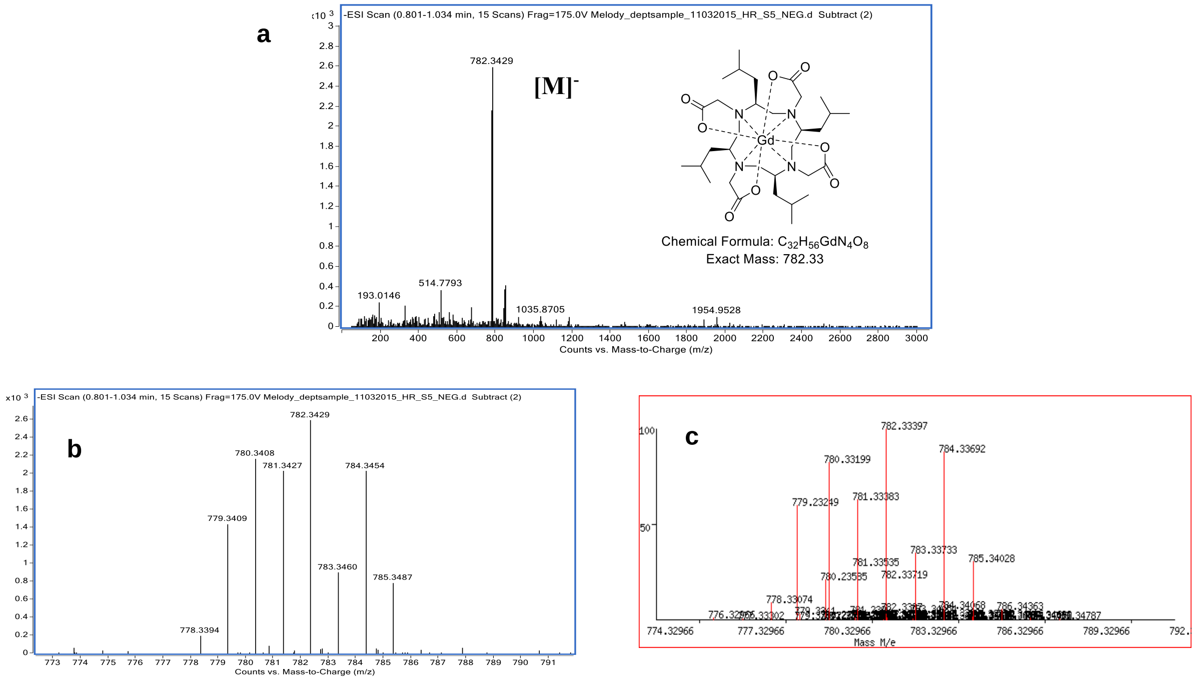

Supplementary Figure 90 | ESI-MS of [GdL3]$^-$: a Full range of the MS in negative pattern; **b** Zoom in spectrum; **c** Calculated isotope distribution of the mass spectrum.

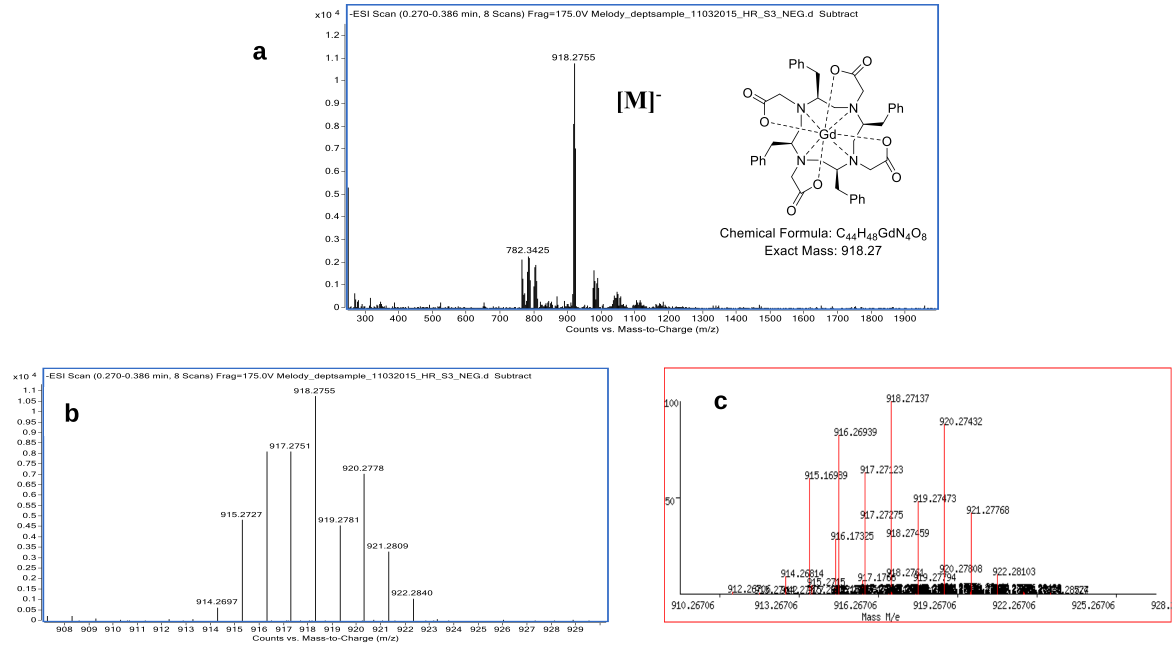

Supplementary Figure 91 | ESI-MS of [GdL4]$^-$: a Full range of the MS in negative pattern; **b** Zoom in spectrum; **c** Calculated isotope distribution of the mass spectrum.

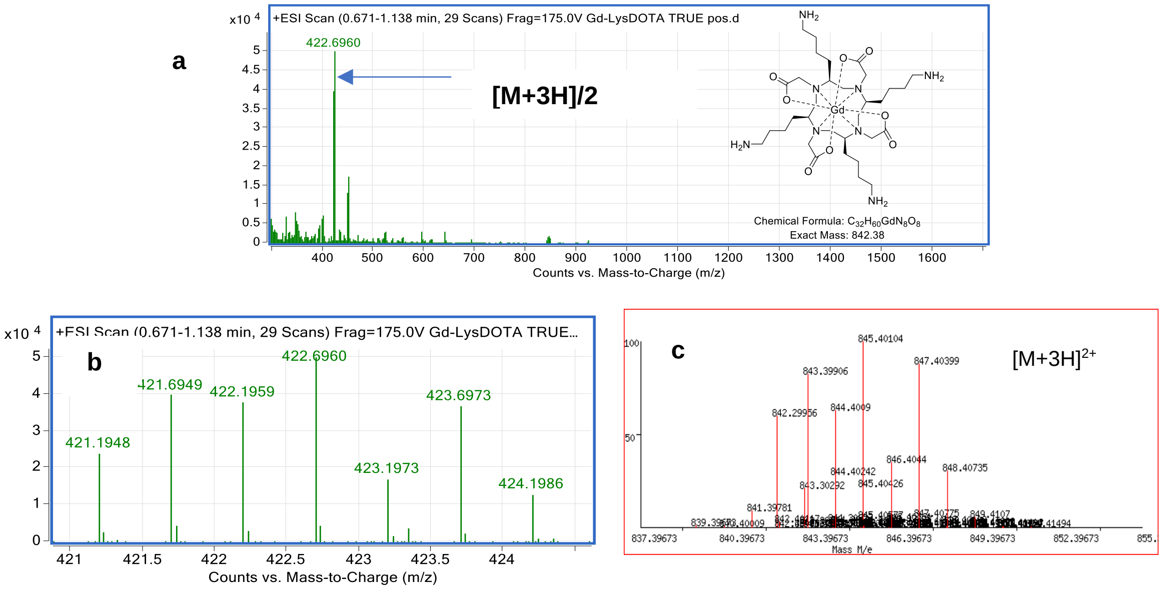

Supplementary Figure 92 | ESI-MS of [GdL5]$^-$: a Full range of the MS in positive pattern; **b** Zoom in spectrum; **c** Calculated isotope distribution of the mass spectrum for $[M+3H]^{2+}$.

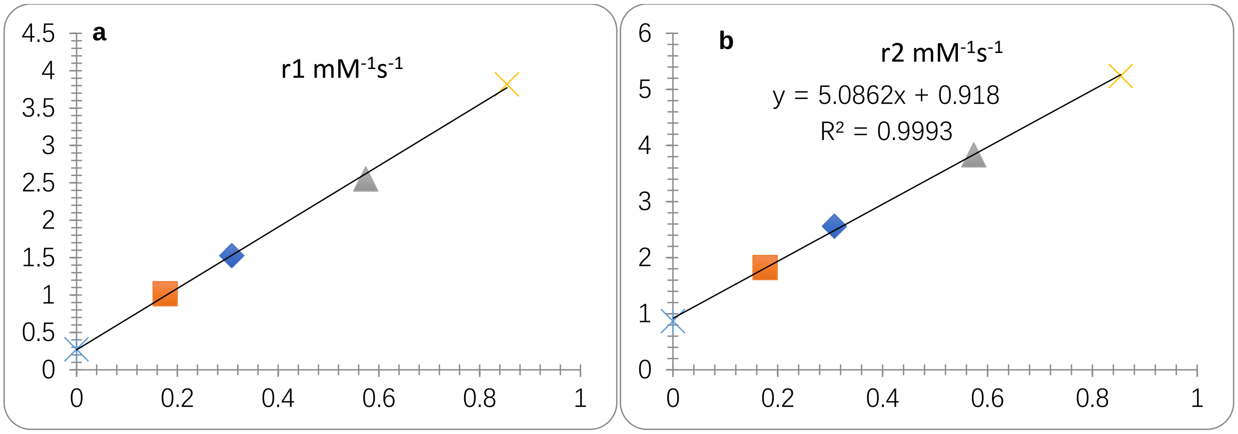

Supplementary Figure 93 | R1 and r2 of [GdL1]$^-$ in water. a Longitudinal (r_1) relaxation rate; **b** Transversal (r_2) relaxation rate.

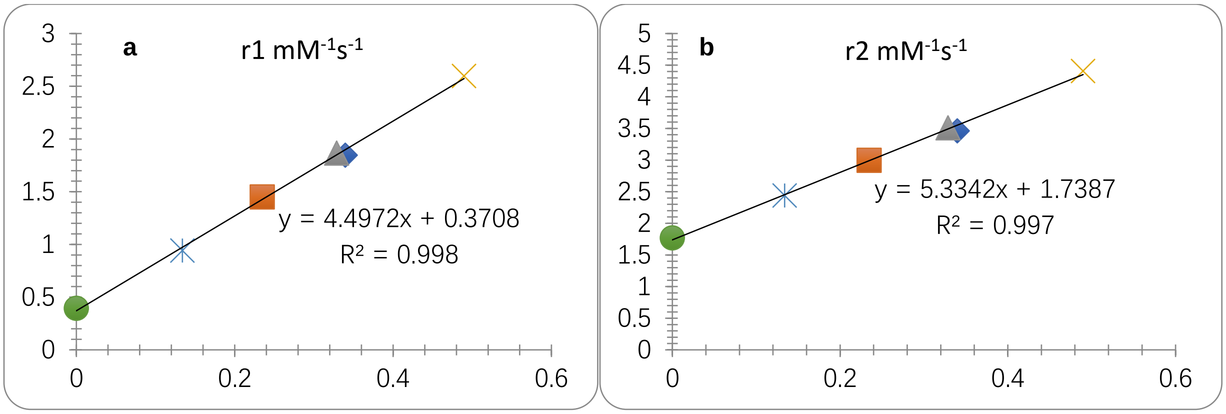

Supplementary Figure 94 | R1 and r2 of [GdL1]$^-$ in HSA. a Longitudinal (r_1) relaxation rate; **b** Transversal (r_2) relaxation rate.

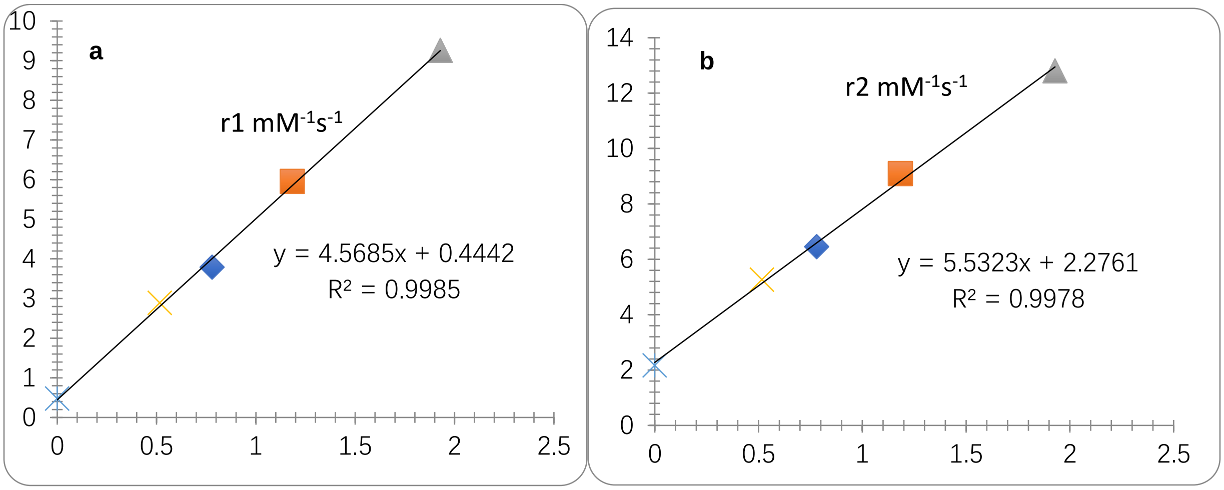

Supplementary Figure 95 | R1 and r2 of [GdL1]$^-$ in Human Plasma. a Longitudinal (r_1) relaxation rate; **b** Transversal (r_2) relaxation rate.

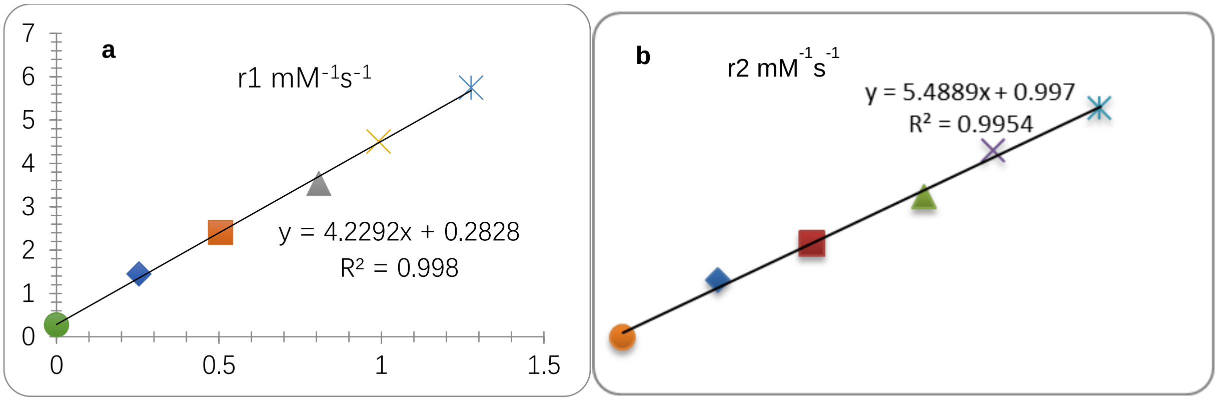

Supplementary Figure 96 | R1 and r2 of [GdL2A]⁻ in water. a Longitudinal (r_1) relaxation rate; **b** Transversal (r_2) relaxation rate.

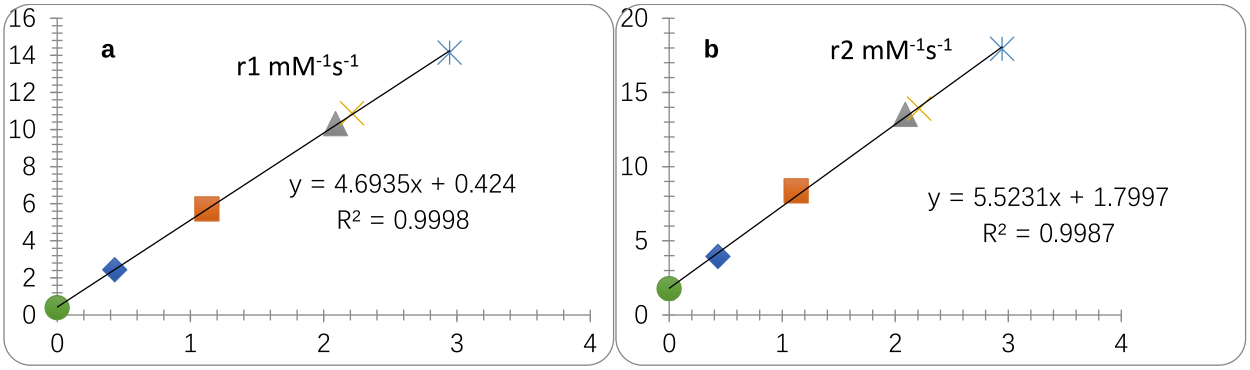

Supplementary Figure 97 | R1 and r2 of [GdL2A]⁻ in HSA. a Longitudinal (r_1) relaxation rate; **b** Transversal (r_2) relaxation rate.

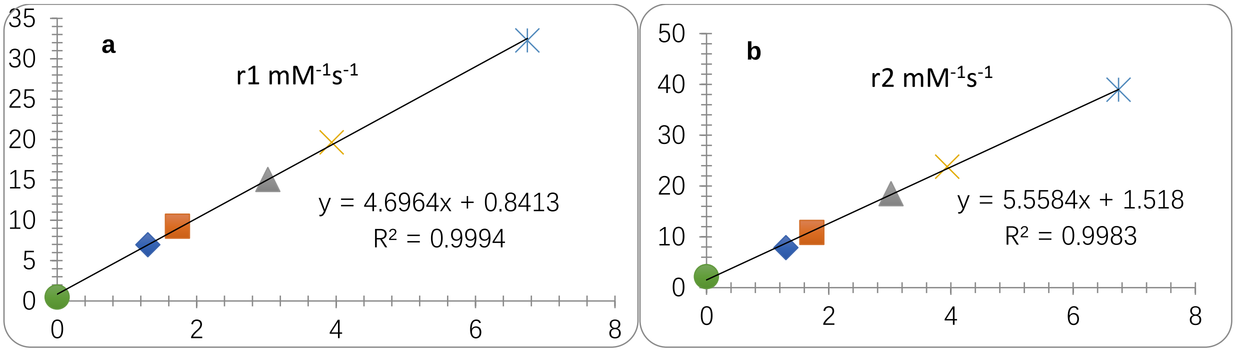

Supplementary Figure 98 | R1 and r2 of [GdL2A]⁻ in Human Plasma. a Longitudinal (r_1) relaxation rate; **b** Transversal (r_2) relaxation rate.

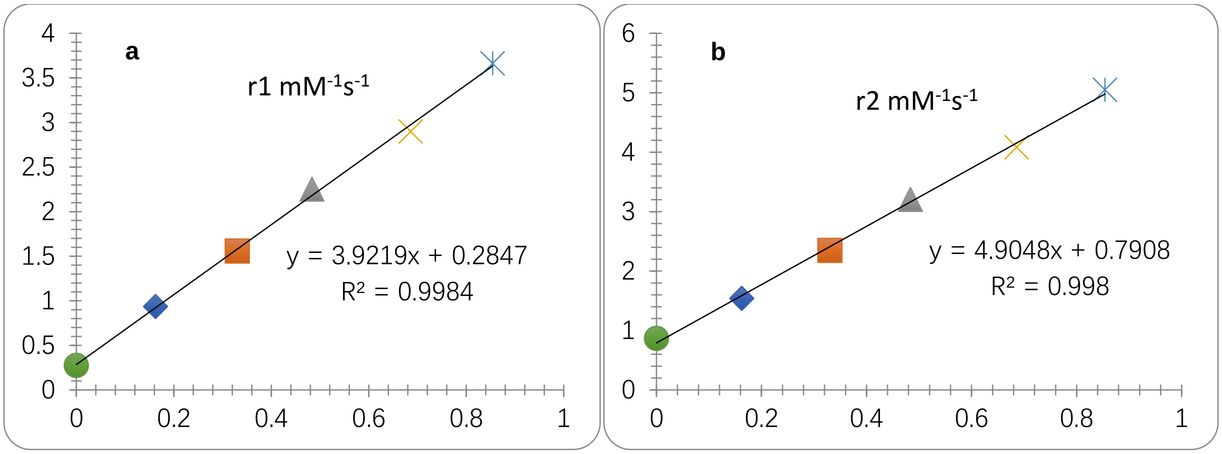

Supplementary Figure 99 | R1 and r2 of [GdL2B]⁻ in water. a Longitudinal (r_1) relaxation rate; **b** Transversal (r_2) relaxation rate.

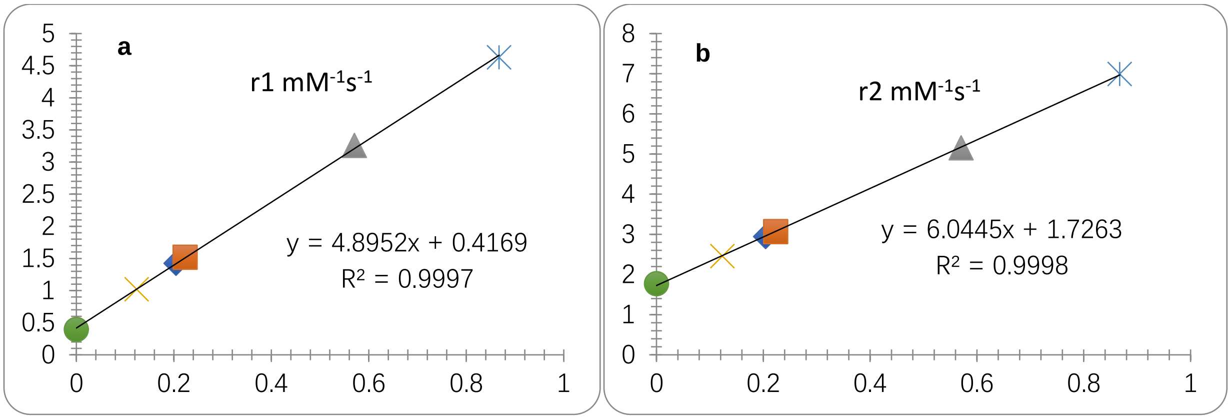

Supplementary Figure 100 | R1 and r2 of [GdL2B]⁻ in HSA. a Longitudinal (r_1) relaxation rate; **b** Transversal (r_2) relaxation rate.

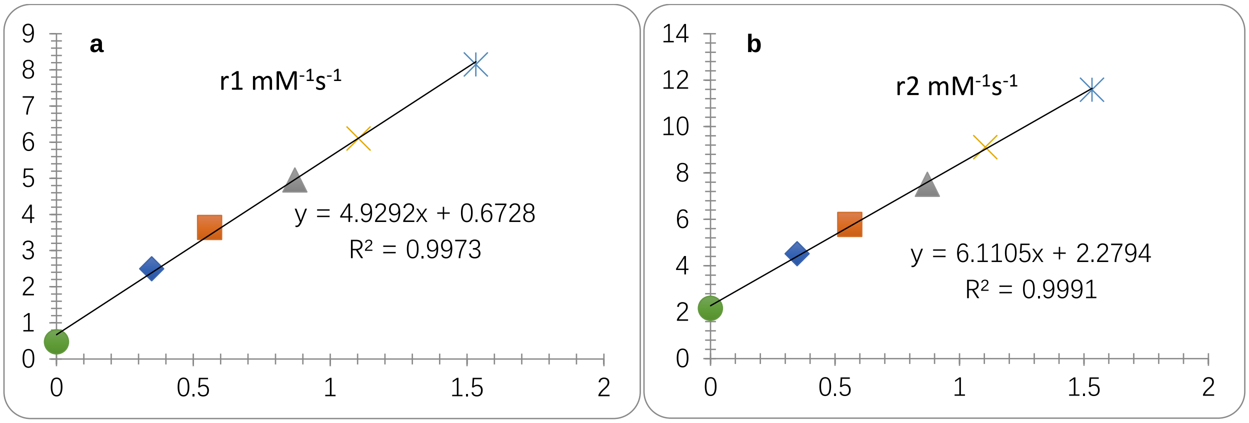

Supplementary Figure 101 | R1 and r2 of [GdL2B]⁻ in Human Plasma: a Longitudinal (r_1) relaxation rate; **b** Transversal (r_2) relaxation rate.

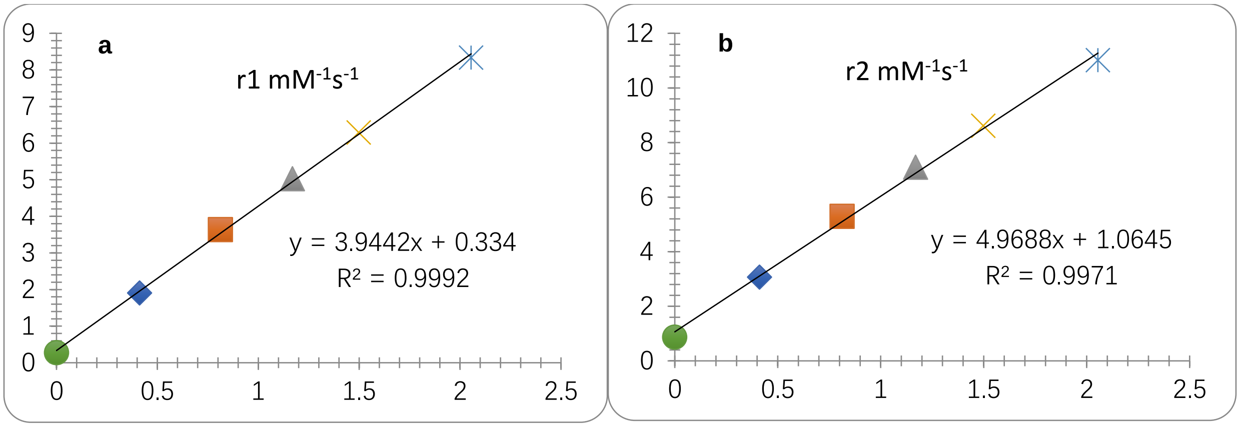

Supplementary Figure 102 | R1 and r2 of [Gd(*R*)L2A]$^-$ water: a Longitudinal (r_1) relaxation rate; **b** Transversal (r_2) relaxation rate.

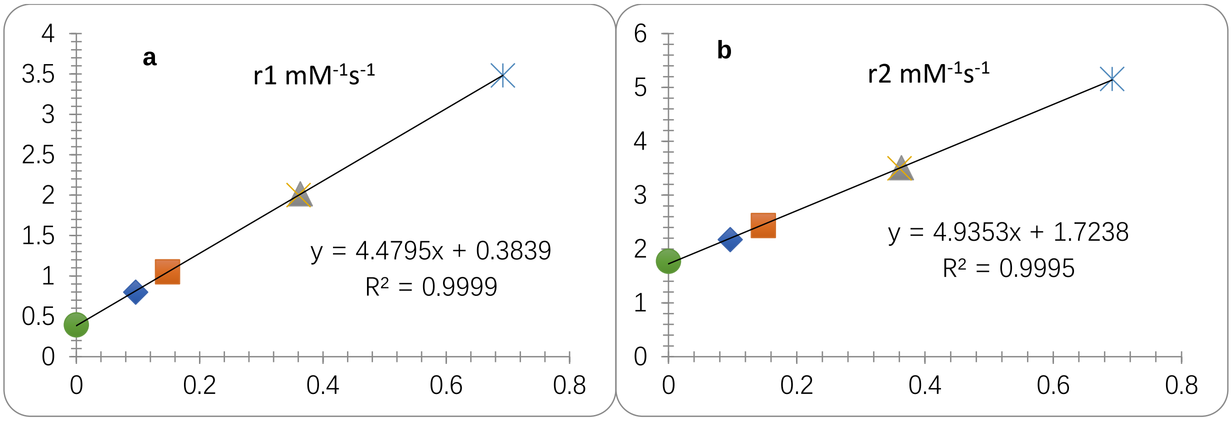

Supplementary Figure 103 | R1 and r2 of [Gd(*R*)L2A]$^-$ in HSA: a Longitudinal (r_1) relaxation rate; **b** Transversal (r_2) relaxation rate.

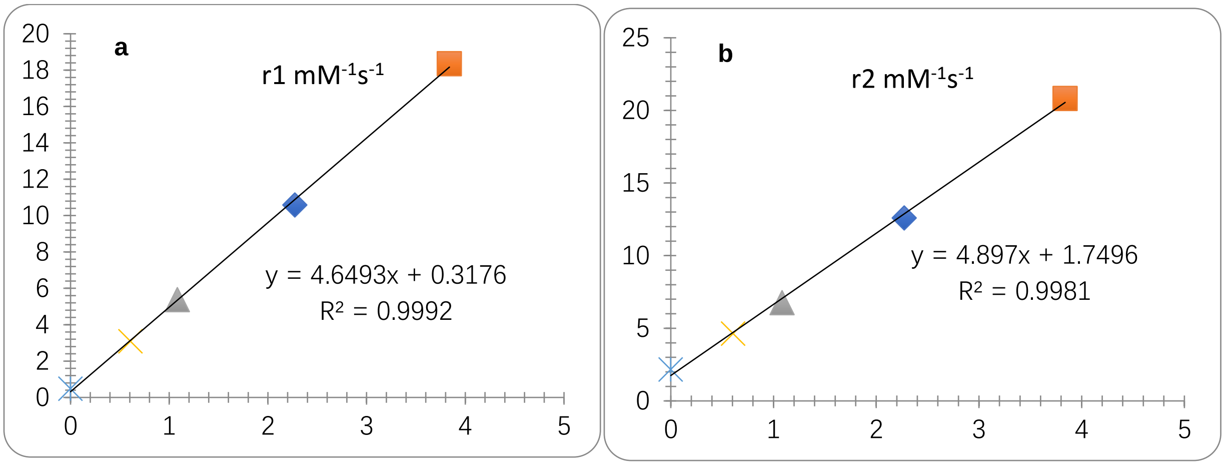

Supplementary Figure 104 | R1 and r2 of [Gd(*R*)L2A]$^-$ in Human Plasma: a Longitudinal (r_1) relaxation rate; **b** Transversal (r_2) relaxation rate.

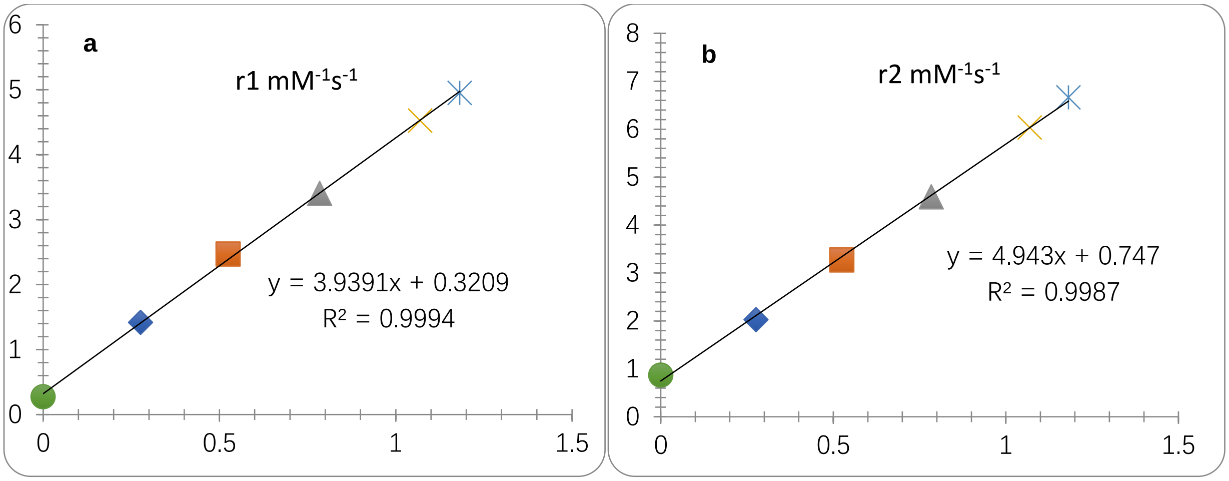

Supplementary Figure 105 | R1 and r2 of [Gd(*R*)L2B]⁻ in water: a Longitudinal (r_1) relaxation rate; **b** Transversal (r_2) relaxation rate.

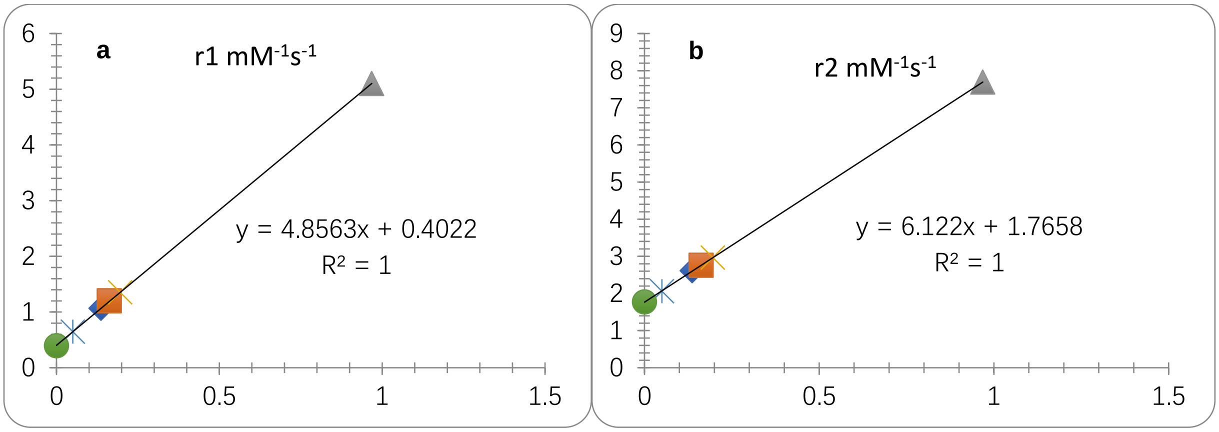

Supplementary Figure 106 | R1 and r2 of [Gd(*R*)L2B]⁻ in HSA: a Longitudinal (r_1) relaxation rate; **b** Transversal (r_2) relaxation rate.

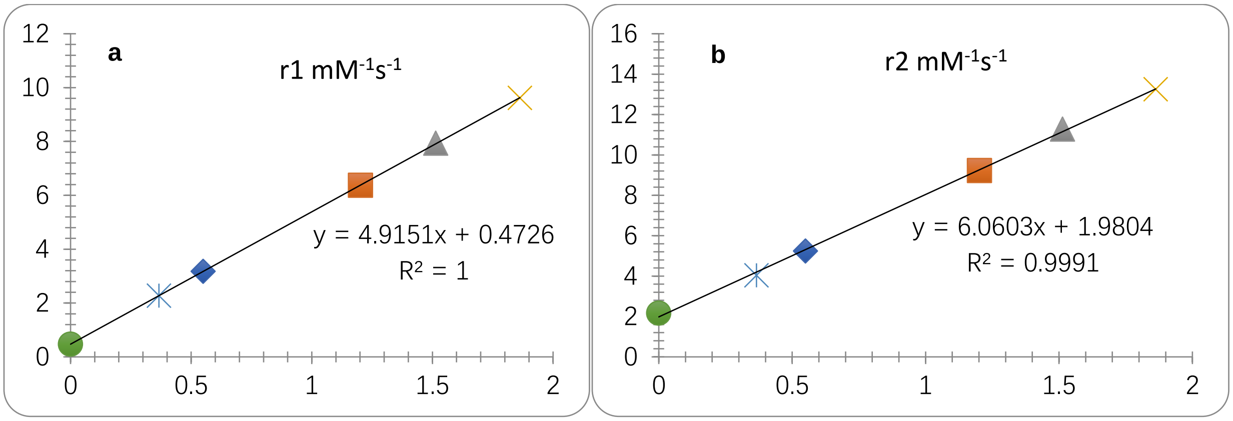

Supplementary Figure 107 | R1 and r2 of [Gd(*R*)L2B]⁻ in Human Plasma: a Longitudinal (r_1) relaxation rate; **b** Transversal (r_2) relaxation rate.

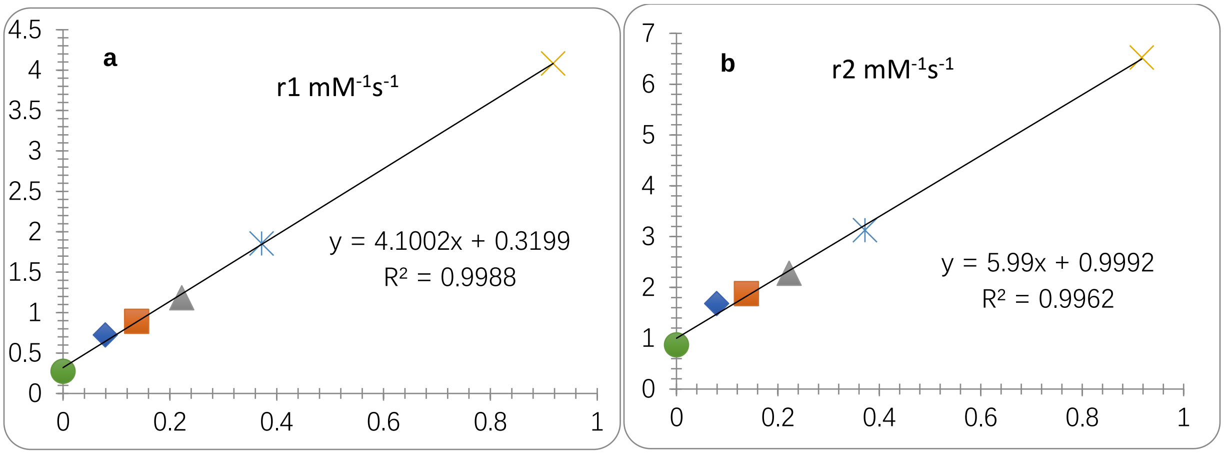

Supplementary Figure 108 | R1 and r2 of [GdL5]⁻ in water: a Longitudinal (r_1) relaxation rate; **b** Transversal (r_2) relaxation rate.

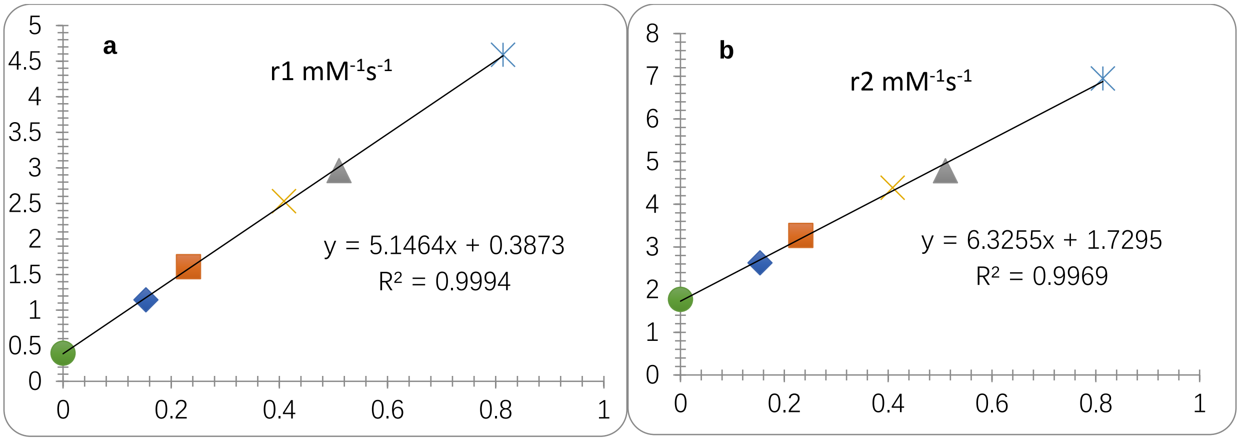

Supplementary Figure 109 | R1 and r2 of [GdL5]⁻ in HSA: a Longitudinal (r_1) relaxation rate; **b** Transversal (r_2) relaxation rate.

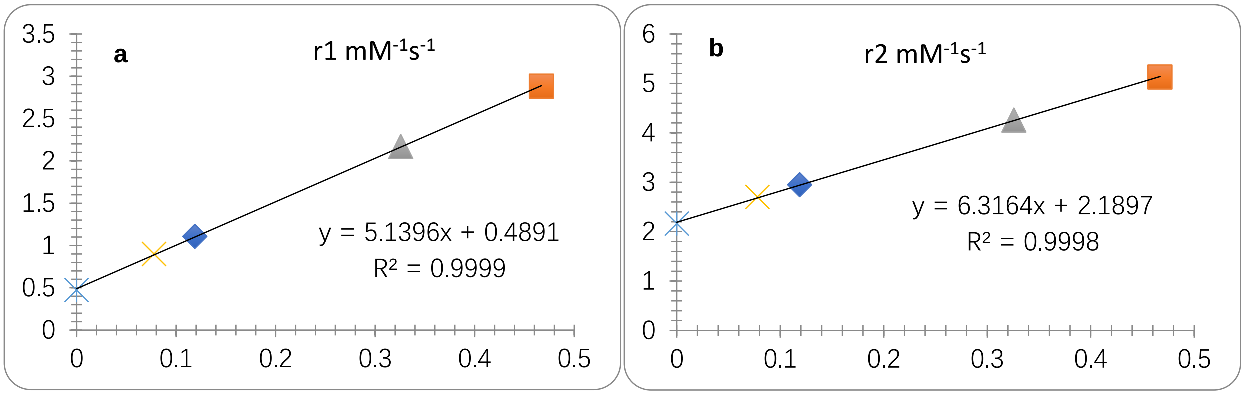

Supplementary Figure 110 | R1 and r2 of [GdL5]⁻ in Human Plasma: a Longitudinal (r_1) relaxation rate; **b** Transversal (r_2) relaxation rate.

Supplementary Tables

	**2b** (*SSSS*-λλλλ)	**2b** (*SSSS*-δδδδ)	**2c**	**[GdL4]$^-$**
Formula	$C_{53}H_{72}N_4$	$C_{50}H_{66}N_4$	$C_{51}H_{68}N_4$	$C_{44}H_{50}Cl_3GdN_4O_9$
Formula weight	765.15	723.07	737.09	1042.48
Temperature (K)	296(2)	296(2)	296(2)	150(2)
Wavelength (Å)	0.71073	0.71073	0.71073	0.71073
Crystal system	Tetragonal	Tetragonal	Tetragonal	Triclinic
Space group	$P4_1$	$P4_1$	$P4_1$	$P1$
a (Å)	12.1060(6)	12.0643(2)	12.0384(3)	10.2782(10)
b (Å)	12.1060(6)	12.0643(2)	12.0384(3)	10.3856(10)
c (Å)	32.510(4)	31.0325(12)	31.9297(16)	23.027(2)
α (deg)	90.00	90.00	90.00	82.380(3)
β (deg)	90.00	90.00	90.00	87.431(3)
γ (deg)	90.00	90.00	90.00	71.094(3)
V ($Å^3$)	4764.5(6)	4516.7(2)	4627.4(3)	2304.9(4)
Z	4	4	4	2
D_c (Mg/m^3)	1.067	1.063	1.058	1.502
μ (mm^{-1})	0.062	0.062	0.061	1.670
F(000)	1672.0	1576.0	1608.0	1058.0
R_1^a [I > 2σ(I)]	0.0464	0.0548	0.0556	0.0725
wR_2^b [I > 2σ(I)]	0.0902	0.1215	0.1336	0.2071
R_1^a (all data)	0.1007	0.1176	0.1190	0.0781
wR_2^b (all data)	0.1077	0.1517	0.1684	0.2109
GOF_c	1.000	1.006	1.017	1.094

[a] $R_1 = \sum||F_o| - |F_c||/ \sum|F_o|$; [b] $wR = \{\sum[w(F_o^2 - F_c^2)]^2\}/\sum[w(F_o^2)_2]\}^{1/2}$. [c] Goodness-of-fit = $\{\sum[w(F_o^2 - F_c^2)]^2\}/(N_{obs} - N_{param})\}^{1/2}$, based on all data.

Supplementary Table 1 | Crystallographic data for compound 2b, 2C and [GdL4]$^-$.

	GdL1	GdL2A	GdL2B	GdL5
blood	1.04	0.49	0.60	0.80
heart	1.68	1.82	3.09	2.18
lung	1.81	3.61	2.63	1.49
liver	1.64	10.47	1.12	2.01
spleen	6.63	4.72	4.82	4.43
kidney	16.71	20.92	7.42	93.23
stomach	11.35	3.14	1.05	1.11
intestines	7.75	0.05	47.07	2.68
tail	4.57	2.13	2.64	2.28
muscle	4.29	5.18	5.43	2.97
gall bladder	139.87	854.21	1066.22	60.17

Supplementary Table 2 | Biodistribution data after MRI study. Data shows the Gd concentration of **[GdL1]**$^-$, **[GdL2A]**$^-$, **[GdL2B]**$^-$ and **[GdL5]**$^-$ at 2 hours post injection in C57/Bl6 mice after the MRI study. Numbers are in nmol Gd per gram tissue.

<table>
<tr><th>Ligand</th><th>Radio metal</th><th>Buffer pH</th><th>Rxn temp (°C)</th><th>Rxn time (min)</th><th>Isomer A%</th><th>Isomer B%</th><th>Overall yield%</th><th>Specific activity (μCi/nmol)</th></tr>
<tr><td rowspan="2">L2</td><td rowspan="2">Cu-64</td><td rowspan="2">6.05</td><td rowspan="2">40</td><td>0</td><td>79.14</td><td>20.86</td><td>100</td><td rowspan="4">200</td></tr>
<tr><td>20</td><td>76.21</td><td>23.79</td><td>100</td></tr>
<tr><td rowspan="2">DOTA</td><td rowspan="2">Cu-64</td><td rowspan="2">6.05</td><td rowspan="2">40</td><td>0</td><td colspan="2">N/A</td><td>88.99</td></tr>
<tr><td>20</td><td colspan="2">N/A</td><td>95.06</td></tr>
</table>

Supplementary Table 3 | Radiolabelling conditions and results for Cu-64.

**Ligand**	**Radio metal**	**Buffer pH**	**Rxn temp (°C)**	**Rxn time (min)**	**Isomer A %**	**Isomer B %**	**Overall yield %**	**SAP/T SAP ratio**	**Specific activity (µCi/nmol)**
**L2**	Lu-177	7.00	95	20	86.41	10.83	97.24	7.98	75
		5.01	95	5	57.83	13.27	71.10	4.36	1250
				10	70.29	15.21	85.50	4.62	
				20	74.53	17.39	91.92	4.29	
				30	76.22	18.11	94.33	4.21	
				40	77.95	17.52	95.47	4.45	
			85	10	62.20	13.20	75.40	4.71	
				20	68.40	14.28	82.68	4.79	
				40	73.20	13.03	86.23	5.62	
				80	73.65	12.77	86.42	5.77	
				80	74.81	11.72	86.53	6.38	
DOTA	Lu-177	5.01	95	5			22.25		1000
				10			27.86		
				20			33.93		
				30			36.14		
				40			38.17		

Supplementary Table 4 | Radiolabelling conditions and results for Lu-177.

Supplementary Methods

Synthesis of (2*S*)-2-(Benzylamino)propan-1-ol.[2] A solution of benzaldehyde (64.2 g, 0.61 mol) and (*S*)-2-amino-1-propanol (50.0 g, 0.67 mol) in dichloromethane (650 ml) and methanol (100 ml) was stirred at room temperature under nitrogen for 16 hours. It was concentrated and the white solid was added with petroleum ether (200 ml), cooled to 0°C and filtered, the filter cake was dried (95 g) and was used to the next step without any further purification. The white solid was dissolved in dichloromethane (650 ml) and methanol (100 ml) and cooled in an ice bath. Powdered sodium borohydride (20.0 g, 0.53 mol) was added in portions and the solution was stirred for 2 hours, water (300 ml) was added and the solution was stirred for overnight. The organic and aqueous phases were separated and the organic phase was washed with brine (200 ml), dried with anhydrous sodium sulphate (50 g), filtered and the filtrate was concentrated under vacuum yielded product (2*S*)-1-Benzyl-2-methylaziridine as a white solid (93 g, 93%). ^{1}H NMR (400 MHz, $CDCl_3$) δ 7.21 – 7.31 (m, 5H), 3.79 (d, *J* = 13.0 Hz, 1H), 3.64 (d, *J* = 13.0 Hz, 1H), 3.49 (dd, *J* = 7.2, 10.7 Hz, 1H), 3.26 (dd, *J* = 7.2, 10.7 Hz, 1H), 2.72 – 2.80 (m, 1H), 1.01 (d, *J* = 6.4 Hz, 3H); ^{13}C NMR (100 MHz, $CDCl_3$) δ 140.10, 128.50, 128.20, 127.09, 65.50, 53.81, 51.10, 16.81.

Synthesis of (*S*)-1-benzyl-2-methylaziridine (1a).[2] Into a soliton of (2*S*)-2-(Benzylamino)propan-1-ol (15.0 g) in diethyl ether (150 ml), added with PPh_3 (33.3 g), then cooled the reaction mixture to 0 - 10 °C, then dropped with DEAD (19.0 g), the resulted mixture was reacted for 16 h at room temperature, then added 500 ml of hexane and the solid was removed by filtration. The filtrate was concentrated and the residue was purified by distillation under vacuum, this resulted in the product as a colourless liquid (12.5 g, yield 93.5%). $[\alpha]^{20}_D$ = +11.44° (c = 0.010034 g / ml, ethanol). ^{1}H NMR (400 MHz, $CDCl_3$) δ 7.25 – 7.37 (m, 5H), 3.43 (q, *J* = 13.6 Hz, 2H), 1.58(d, *J* = 3.6 Hz, 1H), 1.50 – 1.52 (m, 1H), 1.38 (d, *J* = 6.3 Hz, 1H), 1.20 (d, *J* = 5.4 Hz, 3H); ^{13}C NMR (100 MHz, $CDCl_3$) δ 139.56, 128.33, 127.84, 126.88, 64.69, 34.94, 34.79, 18.33.

Synthesis of (*S*)-2-amino-6-((tert-butoxycarbonyl)amino)hexanoic acid. To a solution of L-lysine (40 g, 0.27 mol) and $NaHCO_3$ (23 g, 0.27 mmol) in water (600 ml) and acetone (200 ml) was added $CuSO_4 \cdot 5H_2O$ (34.2 g, 0.14 mol), after stirring at room temperature for 2.5 hours, another 23 g of $NaHCO_3$ was added, then added $(Boc_2)O$ (71 g, 0.33 mol), the reaction mixture was stirring for another 16 hours. The precipitates were collected by filtration, washed with water (500 ml) and ethyl acetate (200 ml). The solid was transferred into another 3 L flask, added with water (600 ml), NaHS (14 g) and $NaHCO_3$ (30 g), then CbzCl (39 g) and THF (300 ml) were added, the mixture was stirring at room temperature for another 16 hours. After that, the pH was adjusted to 4.0 by adding 2 M HCl, ethyl acetate (500 ml) was added, filtrated and the two phases were separated, the aqueous layer was extracted with ethyl acetate (500 ml) again. The combined organic phases were washed one time with water (200 ml). Then dried with anhydrous sodium sulphate, after filtration and concentration, this resulted in 80 g of product and it was used to the next step reaction without any further purification.

Synthesis of (*S*)-benzyl tert-butyl (6-hydroxyhexane-1,5-diyl)dicarbamate.[3] A solution of (*S*)-2-amino-6-((tert-butoxycarbonyl)amino)hexanoic acid (40 g) in dry THF (200 ml) was cooled to 0 – 10 °C, 1,1'-carbonyldiimidazole (CDI) (17.2 g) was added and stirred at this temperature for 1 hour. Then the mixture was transferred into another solution of $NaBH_4$ (8 g) and water (100 ml) carefully (cooled with ice batch first, stirring vigorous), after stirring at room temperature for overnight, the solution was extracted with ethyl acetate (two times, each time 500 ml), combined the organic phases and washed with brine (200 ml), dried with anhydrous sodium sulphate, filtered and concentrated, the residue was purified by column chromatography on silica gel with ethyl acetate and petroleum ether (1:10 to 1:1). This resulted in the product as a colourless oil (25 g, overall yield of 50% from L-lysine) which solidified after several days. ^{1}H NMR (400 MHz, $CDCl_3$): δ 7.32 (m, 5H), 5.12 (s, 1H), 5.11 (s, 2H), 4.62 (s, 1H), 3.62 (m, 3H), 3.11 (m, 2H), 2.04(s, 1H), 1.30 – 1.55 (m, 15H). ^{13}C NMR (100 MHz, $CDCl_3$) δ 156.93, 156.58, 136.37, 128.54, 128.16, 127.96, 79.48, 66.84, 64.84, 53.02, 39.83, 30.57, 28.41, 22.69.

Synthesis of (*S*)-tert-butyl (5-amino-6-hydroxyhexyl)carbamate. Into a solution of (*S*)-benzyl tert-butyl (6-hydroxyhexane-1,5-diyl)dicarbamate (30 g) in ethanol (300 ml) was added Pd/C (10%, wet) (2.5 g), the mixture was hydrogenated under an H_2 atmosphere at room temperature overnight. The mixture was filtrated over celite and evaporated to dryness, this resulted in the product (18 g, yield 94 %) as a colourless oil. ^{1}H NMR (400 MHz,

$CDCl_3$) δ 4.61 (s, 1H), 3.75 – 3.38 (m, 1H), 3.39 – 3.19 (m, 1H), 3.11 (d, *J* = 4.9 Hz, 2H), 2.80 (s, 1H), 1.99 (s, 3H), 1.57 – 1.18 (m, 15H). ^{13}C NMR (100 MHz, $CDCl_3$) δ 156.10, 79.13, 66.66, 52.71, 40.24, 33.98, 30.19, 28.42, 23.16.

Synthesis of (*S*)-tert-butyl (5-(benzylamino)-6-hydroxyhexyl)carbamate. A solution of benzaldehyde (8.0 g, 75.4 mmol) and (*S*)-tert-butyl (5-amino-6-hydroxyhexyl)carbamate (16.0 g, 68.9 mmol) in dichloromethane (208 ml) and methanol (48 ml) was stirred at room temperature under nitrogen for 16 hours. It was concentrated and the residue was pumped with oil pump for two days and washed with petroleum ether for two times (each 200 ml) before the next step reaction. The oil mixture was dissolved in methanol (400 ml) and cooled in an ice bath. Powder sodium borohydride (5.0 g, 0.13 mol) was added in portions and the solution was stirred for 2 hours, water (300 ml) was added and the solution was quenched with 4 M HCl (4 ml), extracted with ethyl acetate (three times, each time 300 ml), combined the organic phases and washed with brine (200 ml), dried with anhydrous sodium sulphate, filtered and the filtrate was concentrated, the residue was purified by column chromatography on silica gel with ethyl acetate and petroleum ether (1:5 to 1:2). This resulted in the product as a colourless oil (13 g, two steps yield 59.1%). ^1H NMR (400 MHz, $CDCl_3$) δ 7.41 – 7.09 (m, 5H), 4.68 (s, 1H), 3.91 – 3.66 (m, 2H), 3.66 – 3.49 (m, 1H), 3.31 (dd, *J* = 10.8, 6.0 Hz, 1H), 3.07 (d, *J* = 6.0 Hz, 2H), 2.71 – 2.47 (m, 1H), 1.55 - 1.06 (m, 15H). ^{13}C NMR (100 MHz, $CDCl_3$) δ 156.12, 140.32, 128.46, 128.12, 127.06, 79.07, 62.83, 58.19, 51.06, 40.24, 31.14, 30.27, 28.43, 23.09.

Synthesis of (*S*)-tert-butyl (4-(1-benzylaziridin-2-yl)butyl)carbamate (1e). A solution of (*S*)-tert-butyl (5-(benzylamino)-6-hydroxyhexyl)carbamate (13 g, 40.3 mmol) in dry THF (150 ml) was cooled to 0 - 10°C, then added PPh_3 (14.8 g, 56.3 mmol) and DEAD (9.8 g, 56.3 mmol), the mixture was stirred for overnight at room temperature. Then concentrated and the residue was purified by column chromatography on silica gel with ethyl acetate and petroleum ether (1:10 to 1:1). This resulted in the product as a colourless oil (8 g, 65.2%). ^1H NMR (400 MHz, $CDCl_3$) δ 7.67 – 7.06 (m, 5H), 4.78 – 4.38 (m, 1H), 4.28 (dd, *J* = 14.1, 7.0 Hz, 1H), 4.14 (ddd, *J* = 28.5, 14.0, 6.9 Hz, 1H), 3.52 (d, *J* = 13.1 Hz, 1H), 3.23 (d, *J* = 13.1 Hz, 1H), 2.99 (d, *J* = 5.7 Hz, 2H), 1.53 – 1.17 (m, 15H). ^{13}C NMR (100 MHz, $CDCl_3$) δ 155.96, 139.32, 128.29, 128.26, 127.03, 78.92, 64.93, 40.52, 39.46, 34.01, 32.44, 29.53, 28.43, 24.73.

Synthesis of (2*S*,5*S*,8*S*,11*S*)-1,4,7,10-tetrabenzyl-2,5,8,11-tetramethyl-1,4,7,10-tetraazacyclododecane (2a).[2] Into a stirring solution of (*S*)-1-benzyl-2-methylaziridine (2.0 g) in methanol (25 ml) was added the catalyst of TsOH (100 mg), the reaction mixture was stirring at room temperature and additional TsOH was added every other day (100 mg each time), after stirring for 6 days, the resulted mixture was purified by column chromatography by methanol. The fractions of the product were combined and concentrated to 5 ml, then 3 ml of ammonia solution was added, then the precipitate was collected by filtration and washed thoroughly by methanol. The filter cake was dried by air to get a white solid (240 mg, yield 12%). $[\alpha]^{20}_D$ = -55.24° (c = 0.01016 g / ml, benzene). ^1H NMR (400 MHz, $CDCl_3$) δ 7.18 - 7.60 (m, 20H), 3.32 - 3.50 (m, 12H), 3.01 (dd, *J* = 7.9, 13.0 Hz, 4H), 1.99 (dd, *J* = 5.4, 13.1 Hz, 4H), 0.90 (d, *J* = 6.5 Hz, 12H); ^{13}C NMR (100 MHz, $CDCl_3$) δ 140.99, 128.96, 127.87, 126.40, 54.52, 51.81, 51.21, 12.48; HRMS (m/z): $[M+H]^+$ calcd. for $C_{40}H_{53}N_4$, 589.4270; found 589.4269.

Synthesis of (2*S*,5*S*,8*S*,11*S*)-1,4,7,10-Tetrabenzyl-2,5,8,11-tetraethyl-1,4,7,10-tetraazacyclododecan (2b).[4] To a solution of (2*S*)-1-Benzyl-2-ethylaziridine (6 g, 37.2 mmol) in dried benzene (75 ml) was added $BF_3 \cdot Et_2O$ (0.1 ml), the mixture was stirred under reflux for 24 hrs. Then cooled and 2 ml of saturated sodium bicarbonate was added, then concentrated and the residue was purified by recrystallization in benzene and ethyl acetate, inorganic salts were washed by water. The solid was dried in oven and this resulted in product as a white solid (1.65 g, 27.5%). $[\alpha]^{20}_D$ = -341.57° (c = 0.01012 g/ml, benzene). ^1H NMR (400 MHz, $CDCl_3$) δ 7.22 - 7.31 (m, 20 H), 3.70(d, J=13.9, 4 H), 3.00 - 3.12 (m, 12 H), 2.12 (m, 4 H), 1.74 (m, 4 H), 1.30(m, 4 H), 1.02 (t, *J*=7.0 Hz, 12 H); ^{13}C NMR (100 MHz, $CDCl_3$) δ140.90, 128.65, 128.03, 126.48, 55.80, 52.69, 47.74, 22.82, 11.87.

Synthesis of (2*S*,5*S*,8*S*,11*S*)-1,4,7,10-tetrabenzyl-2,5,8,11-tetraisobutyl-1,4,7,10-tetraazacyclododecane (2c). To a solution of (2*S*)-1-Benzyl-2-isobutylaziridine (6 g) in dried benzene (70 ml) was added $BF_3 \cdot Et_2O$ (0.1 ml), the mixture was stirred under reflux for 24 hrs. Then cooled and 2 ml of saturated sodium bicarbonate was added, then concentrated and the residue was purified by recrystallization in acetonitrile and methanol, inorganic salts were washed by water. The solid was dried in oven and this resulted in product as a white solid (1.8 g, yield

30%). ^{1}H NMR (400 MHz, $CDCl_3$) δ 7.71 – 6.89 (m, 20H), 3.75 (d, *J* = 14.0 Hz, 4H), 3.30 (s, 4H), 3.14 (d, *J* = 12.4 Hz, 4H), 2.91 (d, *J* = 14.0 Hz, 4H), 2.13 – 1.79 (m, 8H), 1.52 (d, *J* = 25.2 Hz, 4H), 1.23 (d, *J* = 13.7 Hz, 4H), 0.99 (d, *J* = 6.5 Hz, 12H), 0.90 (d, *J* = 6.7 Hz, 12H); ^{13}C NMR (100 MHz, $CDCl_3$) δ 140.92, 128.49, 128.08, 126.51, 52.24, 50.38, 47.51, 40.22, 24.37, 23.98, 22.39. ESI-MS (m/z): $[M+H]^+$ calcd. for $C_{52}H_{77}N_4$, 758; found 758.

Synthesis of (2S,5S,8S,11S)-1,2,4,5,7,8,10,11-octabenzyl-1,4,7,10-tetraazacyclododecane (2d). To a solution of (2*S*)-1-Benzyl-2-benzylaziridine (6 g) in dried benzene (60 ml) was added $BF_3{\cdot}Et_2O$ (0.2 ml), the mixture was stirred under reflux for 24 hrs. Then cooled and 0.5 ml of triethyl amine was added, then concentrated and the residue was purified by recrystallization in ethyl acetate and methanol. The solid was dried in oven and this resulted in product as a white solid (2.1 g, yield 35%). ^{1}H NMR (400 MHz, $CDCl_3$) δ 7.15 (dtd, *J* = 53.1, 14.1, 6.8 Hz, 32H), 6.70 (d, *J* = 7.0 Hz, 8H), 3.76 (d, *J* = 14.4 Hz, 4H), 3.36 – 3.08 (m, 8H), 2.99 (dd, *J* = 12.7, 2.9 Hz, 4H), 2.47 – 2.24 (m, 8H), 2.18 (dd, *J* = 12.5, 8.1 Hz, 4H); ^{13}C NMR (100 MHz, $CDCl_3$) δ 141.12, 140.03, 129.77, 128.39, 128.08, 126.52, 125.82, 56.80, 52.30, 47.31, 35.47. ESI-MS (m/z): $[M+H]^+$ calcd. for $C_{64}H_{69}N_4$, 894; found 894.

Synthesis of tetra-tert-butyl (((2*S*,5*S*,8*S*,11*S*)-1,4,7,10-tetrabenzyl-1,4,7,10-tetraazacyclododecane-2,5,8,11-tetrayl)tetrakis(butane-4,1-diyl))tetracarbamate (2e). Into a solution of (*S*)-tert-butyl (4-(1-benzylaziridin-2-yl)butyl)carbamate, **1e** (5.3 g, 17.4 mmol) in acetonitrile (212 ml) was added $TsOH{\cdot}H_2O$ (230 mg), the solution was stirred at room temperature and added 230 mg of $TsOH{\cdot}H_2O$ every 24 hours, for 6 days. The reaction was quenched with 2% of K_2CO_3 in water (212 ml). The precipitate formed was collected by filtration followed by washing with water. The product was collected as a white solid (1.3 g, yield 24.5%) after air drying. ^{1}H NMR (400 MHz, $CDCl_3$) δ 7.62 – 6.85 (m, 20H), 4.99 (br, 4H), 3.62 (d, *J* = 13.7 Hz, 4H), 3.32 – 2.74 (m, 20H), 2.12 – 1.90 (m, 4H), 1.60 – 1.05 (m, 60H). ^{13}C NMR (100 MHz, $CDCl_3$) δ 156.15, 140.52, 128.70, 128.11, 126.63, 78.88, 77.22, 52.74, 47.98, 40.79, 30.31, 29.73, 28.51, 24.19. ESI-MS (m/z): $[M+H]^+$ calcd. for $C_{72}H_{113}N_8O_8$, 1218; found 1218.

Synthesis of (2*S*,5*S*,8*S*,11*S*)-2,5,8,11-tetramethyl-1,4,7,10-tetraazacyclododecane (3a). Into the solution of **2a** (600 mg) in trifluoroethanol (15 ml) was added $Pd(OH)_2/C$ (100 mg) and ammonium formate (300 mg), after reacting at 50 °C for 16 h, a filtration was performed and the filtrate was concentrated under vacuum to get the product a light yellow solid (0.22 g, yield 94.5%). ^{1}H NMR (400 MHz, $CDCl_3$+D_2O) δ 2.60 – 2.66 (m, 8H), 2.36 (m, 4H), 0.92 (d, *J* = 6.0 Hz, 12H); ^{13}C NMR (100 MHz, $CDCl_3$) δ 52.02, 47.663, 18.24; ESI-MS (m/z): $[M+H]^+$ calcd. for $C_{12}H_{29}N_4$, 229; found MS: 229.

Synthesis of (2*S*,5*S*,8*S*,11*S*)-2,5,8,11-tetraethyl-1,4,7,10-tetraazacyclododecane (3b). Into the solution of **2b** (3.5 g) in trifluoroethanol (100 ml) was added $Pd(OH)_2/C$ (400 mg) and ammonium formate (900 mg), after reacting at 50 °C for 16 h, a filtration was performed and the filtrate was concentrated under vacuum to get the product a light yellow solid (1.5 g, yield 97%). ^{1}H NMR (400 MHz, $CDCl_3$) δ 2.71 – 2.74 (d, *J* = 10.4 Hz, 4H), 2.23 – 2.43 (m, 12H), 1.18 – 1.29 (m, 4H), 0.87 (t, *J* = 7.5 Hz, 12H); ^{13}C NMR (100 MHz, $CDCl_3$) δ 54.60, 49.17, 25.27, 10.28; HRMS (m/z): $[M+H]^+$ calcd. for $C_{16}H_{37}N_4$, 285.3018; found 285.3022.

Synthesis of (2*S*,5*S*,8*S*,11*S*)-2,5,8,11-tetraisobutyl-1,4,7,10-tetraazacyclododecane (3c). Into the solution of **2c** (2.8 g) in trifluoroethanol (100 ml) was added $Pd(OH)_2/C$ (350 mg) and ammonium formate (800 mg), after reacting at 50 °C for 16 h, a filtration was performed and the filtrate was concentrated under vacuum to get the product as a white solid (1.4 g, yield 95%). ^{1}H NMR (400 MHz, $CDCl_3$) δ 2.86 – 2.63 (m, 4H), 2.55 (d, *J* = 9.0 Hz, 4H), 2.34 (dd, *J* = 13.0, 9.6 Hz, 4H), 1.91 (s, 8H), 1.59 (dq, *J* = 12.8, 6.5 Hz, 4H), 1.38 (ddd, *J* = 13.1, 9.4, 3.6 Hz, 4H), 1.05 (ddd, *J* = 13.5, 8.7, 4.8 Hz, 4H), 0.90 (dd, *J* = 9.5, 6.6 Hz, 24H); ^{13}C NMR (100 MHz, $CDCl_3$) δ 51.32, 50.09, 42.58, 25.28, 23.84, 22.25; HRMS (m/z): $[M+H]^+$ calcd. for $C_{24}H_{53}N_4$, 397.4270; found 397.4273.

Synthesis of (2*S*,5*S*,8*S*,11*S*)-2,5,8,11-tetrabenzyl-1,4,7,10-tetraazacyclododecane (3d). Into the solution of **2d** (2.8 g) in trifluoroethanol (100 ml) was added $Pd(OH)_2/C$ (500 mg) and ammonium formate (1.5 g), after reacting at 75 °C for 16 h, a filtration was performed and the filtrate was concentrated under vacuum, the residue was purified by column chromatography ($CHCl_3$ / EtOH 10:1), this resulted in the product as a light yellow solid (1.2 g, yield 71.9%). ^{1}H NMR (400 MHz, $CDCl_3$) δ 7.65 – 6.99 (m, 12H), 6.91 (d, *J* = 6.7 Hz, 8H), 2.86 – 2.68 (m,

8H), 2.62 (d, *J* = 8.6 Hz, 4H), 2.51 (dd, *J* = 12.9, 9.7 Hz, 4H), 2.32 (dd, *J* = 13.2, 8.4 Hz, 4H); ^{13}C NMR (100 MHz, $CDCl_3$) δ 141.12, 140.03, 129.77, 128.39, 128.08, 126.52, 125.82, 56.80, 52.30, 47.31, 35.47; HRMS (m/z): $[M+H]^+$ calcd. for $C_{36}H_{45}N_4$, 533.3644; found 533.365.

Synthesis of tetra-tert-butyl (((2*S*,5*S*,8*S*,11*S*)-1,4,7,10-tetraazacyclododecane-2,5,8,11-tetrayl)tetrakis(butane-4,1-diyl))tetracarbamate (3e). Into the solution of 2e (1.8 g) in trifluoroethanol (60 ml) was added $Pd(OH)_2$/C (300 mg) and ammonium formate (700 mg), after reacting at 50 °C for 16 h, a filtration was performed and the filtrate was concentrated under vacuum to get the product as a white solid as a light yellow solid (1.2 g, yield 92.3%). ^{1}H NMR (400 MHz, $CDCl_3$) δ 4.81 (s, 4H), 3.10 (t, *J* = 33.9 Hz, 8H), 2.59 (dd, *J* = 40.9, 9.9 Hz, 4H), 2.42 (s, 4H), 2.40 – 2.19 (m, 4H), 1.53 – 0.99 (m, 60H). ^{13}C NMR (100 MHz, $CDCl_3$) δ 156.07, 78.89, 53.34, 48.76, 40.29, 32.15, 30.32, 28.37, 23.16. ESI-MS (m/z): $[M+H]^+$ calcd. for $C_{44}H_{89}N_8O_8$, 858; found 858.

Synthesis of tetra-tert-butyl 2,2',2'',2'''-((2*S*,5*S*,8*S*,11*S*)-2,5,8,11-tetramethyl-1,4,7,10-tetraazacyclododecane-1,4,7,10-tetrayl)tetraacetate (4a). To a solution of **3a** (120 mg, 0.53 mmol) in dry acetonitrile (5 ml) was added potassium carbonate (0.73 g, 5.25 mmol) and tert-butyl 2-bromoacetate (512.4 mg, 2.6 mmol), the mixture was stirred at 50 °C for 16 hours. Then cooled to room temperature and filtered, the filtrate was concentrated, the residue was dissolved into 60 ml of 2% HCl, then extracted with ethyl acetate (30 ml) twice. The pH of the aqueous phase was adjusted to 8 by adding sat. $NaHCO_3$, then extracted with dichloromethane (30 ml) twice. The combined dichloromethane phases were dried over anhydrous magnesium sulphate, filtered and the filtrate was concentrated under vacuum, this resulted in the product **4a** as a white solid (350 mg, yield 97.2%). ^{1}H NMR (400 MHz, $CDCl_3$) δ 3.27 - 3.02 (m, 8H), 2.79 - 1.89 (m, 12H), 1.41 (s, 36H), 0.86 (dd, *J* = 25.2, 4.1 Hz, 12H); ^{13}C NMR (100 MHz, $CDCl_3$) δ 173.16, 173.02, 81.93, 77.28, 55.12, 53.33, 52.96, 50.36, 49.82, 48.07, 27.90, 10.27; ESI-MS (m/z): $[M+H]^+$ calcd. for $C_{36}H_{69}N_4O_8$, 685; found 685.

Synthesis of tetra-tert-butyl 2,2',2'',2'''-((2*S*,5*S*,8*S*,11*S*)-2,5,8,11-tetraethyl-1,4,7,10-tetraazacyclododecane-1,4,7,10-tetrayl)tetraacetate (4b). To a solution of **3b** (306 mg) in dry acetonitrile (15 ml) was added potassium carbonate (0.73 g, 5.25 mmol) and tert-butyl 2-bromoacetate (1.26 g), the mixture was stirred at 50 °C for 16 hours. Then cooled to room temperature and filtered, the filtrate was concentrated, the residue was dissolved into 60 ml of 2% HCl, then extracted with ethyl acetate (30 ml) twice. The pH of the aqueous phase was adjusted to 8 by adding sat. $NaHCO_3$, then extracted with dichloromethane (40 ml) twice. The combined dichloromethane phases were dried over anhydrous magnesium sulphate, filtered and the filtrate was concentrated under vacuum, this resulted in a white solid (530 mg, yield 66.5%). ^{1}H NMR (400 MHz, $CDCl_3$) δ 3.24 (d, *J* = 17.4 Hz, 4H), 2.80 – 2.58 (m, 10H), 2.15 – 2.06 (m, 6H), 2.20 – 1.96 (m, 1H), 1.66 (m, 4H), 1.41 (s, 36H), 0.90 (m,16H); ^{13}C NMR (100 MHz, $CDCl_3$) δ 172.96, 82.22, 56.60, 51.70, 50.71, 27.88, 17.95, 12.78. HRMS (m/z): $[M+Na]^+$ calcd. for $C_{40}H_{76}N_4O_8Na$, 763.5561; found 763.5541.

Synthesis of tetra-tert-butyl 2,2',2'',2'''-((2S,5S,8S,11S)-2,5,8,11-tetraisobutyl-1,4,7,10-tetraazacyclododecane-1,4,7,10-tetrayl)tetraacetate (4c). To a solution of **3c** (153 mg, 0.39 mmol) in dried acetonitrile (5 ml) was added potassium carbonate (533.1 mg, 3.9 mmol) and tert-butyl 2-bromoacetate (376 mg, 1.9 mmol), the mixture was stirred at 65 °C for 20 hours. Then cooled to room temperature and filtered, the filtrate was concentrated, the residue was purified by column chromatography on silica gel ($CHCl_3$ with 1% - 5% of EtOH), this resulted in a white solid (160 mg, yield 48.6%). ^{1}H NMR (400 MHz, $CDCl_3$) δ 3.25 (dd, *J* = 25.4, 17.4 Hz, 4H), 3.02 (d, *J* = 17.4 Hz, 2H), 2.78 (dt, *J* = 25.4, 14.4 Hz, 4H), 2.62 – 2.37 (m, 4H), 2.24 – 2.07 (m, 2H), 1.99 (d, *J* = 13.5 Hz, 2H), 1.56 – 1.02 (m, 48H), 0.95 (t, *J* = 11.2 Hz, 12H), 0.84 (dd, *J* = 10.9, 6.4 Hz, 12H); ^{13}C NMR (100 MHz, $CDCl_3$) δ 172.84, 172.77, 82.11, 82.01, 55.60, 52.37, 52.27, 52.18, 50.57, 45.81, 34.87, 33.77, 27.87, 27.75, 27.70, 25.73, 25.48, 24.13, 23.88, 21.60, 21.19; HRMS (m/z): $[M+Na]^+$ calcd. for $C_{48}H_{92}N_4O_8Na$, 875.6813; found 875.6827.

Synthesis of tetra-tert-butyl 2,2',2'',2'''-((2*S*,5*S*,8*S*,11*S*)-2,5,8,11-tetrabenzyl-1,4,7,10-tetraazacyclododecane-1,4,7,10-tetrayl)tetraacetate (4d). To a solution of **3d** (204 mg, 0.39 mmol) in dried acetonitrile (5 ml) was added potassium carbonate (529 mg) and tert-butyl 2-bromoacetate (448 mg), the mixture was stirred at 65 °C for 20 hours. Then cooled to room temperature and filtered, the filtrate was concentrated, the residue was purified by column chromatography on silica gel ($CHCl_3$ with 1% - 5% of EtOH), this resulted in

this resulted in a white solid (272 mg, yield 71.8%). ^{1}H NMR (400 MHz, $CDCl_3$) δ 7.27 – 7.14 (m, 13H), 6.11 (dd, *J* = 17.4, 7.3 Hz, 7H), 3.48 (dd, *J* = 32.0, 17.4 Hz, 4H), 3.16 – 2.83 (m, 4H), 2.82 – 2.24 (m, 16H), 1.89 (t, *J* = 11.2 Hz, 4H), 1.50 (s, 36H); ^{13}C NMR (100 MHz, $CDCl_3$) δ 173.88, 173.51, 140.04, 140.00, 130.31, 130.11, 129.90, 127.46, 83.73, 83.61, 59.26, 59.12, 51.96, 51.82, 51.48, 51.17, 32.90, 29.03, 28.99. HRMS (m/z): $[M+Na]^+$ calcd. for $C_{60}H_{84}N_4O_8Na$, 1011.6187; found 1011.6191.

Synthesis of tetra-tert-butyl 2,2',2'',2'''-((2*S*,5*S*,8*S*,11*S*)-2,5,8,11-tetrakis(4-((tert-butoxycarbonyl)amino)butyl)-1,4,7,10-tetraazacyclododecane-1,4,7,10-tetrayl)tetraacetate (4e). To a solution of **3e** (1.4 g) in dried acetonitrile (20 ml) was added potassium carbonate (2.2 g) and tert-butyl 2-bromoacetate (1.9 g), the mixture was stirred at 65 °C for 20 hours. Then cooled to room temperature and filtered, the filtrate was concentrated, the residue was purified by column chromatography on silica gel ($CHCl_3$ with 1% - 5% of EtOH), resulted in the product as a white solid (1.3 g, 65.3%). ^{1}H NMR (400 MHz, $CDCl_3$) δ 5.64 (s, 4H), 3.23 (dd, *J* = 49.7, 12.4 Hz, 4H), 3.04 (d, *J* = 5.8 Hz, 8H), 2.72 (d, *J* = 16.5 Hz, 8H), 2.43 (t, *J* = 45.5 Hz, 4H), 2.23 – 1.89 (m, 4H), 1.68 – 0.95 (m, 96H). ^{13}C NMR (100 MHz, $CDCl_3$) δ 172.94, 156.38, 82.03, 78.58, 54.31, 52.39, 50.66, 40.03, 30.28, 28.44, 28.04, 27.83, 25.18, 25.06. ESI-MS (m/z): $[M+H]^+$ calcd. for $C_{68}H_{129}N_8O_{16}$, 1314; found 1314.

2,2',2'',2'''-((2*S*,5*S*,8*S*,11*S*)-2,5,8,11-tetramethyl-1,4,7,10-tetraazacyclododecane-1,4,7,10-tetrayl)tetraacetic acid (L1). The solution of **4a** (350 mg, 0.51 mmol) in dried dichloromethane (2 ml) and trifluoroacetic acid (2 ml) was stirred at room temperature for two days, the solvents were concentrated and resulted the deprotected product as a light yellow solid (in the form of TFA salt) (460 mg, yield 98.2%), it was used directly in the next step without further purification. ^{1}H NMR (400 MHz, D_2O) δ 4.41 – 2.44 (m, 20H), 1.34 – 1.14 (d, *J* = 6.1 Hz, 6H), 1.05 (d, *J* = 5.2 Hz, 6H); ^{13}C NMR (100 MHz, D_2O) δ 174.42, 168.43, 163.34, 162.99, 162.64, 162.28, 120.66, 117.76, 114.86, 111.96, 57.42, 53.94, 52.37, 49.51, 49.23, 48.46, 10.11, 8.96.

2,2',2'',2'''-((2*S*,5*S*,8*S*,11*S*)-2,5,8,11-tetraethyl-1,4,7,10-tetraazacyclododecane-1,4,7,10-tetrayl)tetraacetic acid (L2). The solution of **4b** (50 mg, 0.067 mmol) in dried dichloromethane (1 ml) and trifluoroacetic acid (1 ml) was stirred at room temperature for 16 hours, the solvents were concentrated and then added 2 ml of 1 M hydrochloride acid, the acidic solution was concentrated under vacuum and the residue was dissolved in 1 ml of water, the solution was lyophilized, this resulted in the product as an off-white powder (42 mg, yield 94.0%), it was used directly in the next step without further purification. TFA salt: ^{1}H NMR (400 MHz, DMSO-d_6) δ 7.50 (br, 2H), 2.74 – 4.23 (m, 20H), 1.94 (m, 4H), 1.22 – 1.37 (m, 4H), 0.97 (m, 12 H); HCl salt: ^{1}H NMR (400 MHz, D_2O) δ 4.33 – 2.54 (m, 20H), 2.01 – 1.69 (m, 4H), 1.45 – 1.10 (m, 4H), 0.90 (dd, *J* = 40.1, 6.5 Hz, 12H); ^{13}C NMR (100 MHz, D_2O) δ 174.30, 168.20, 163.10, 162.75, 162.39, 162.03, 120.57, 117.67, 114.78, 111.88, 62.68, 55.60, 52.23, 51.37, 49.67, 48.88, 17.81, 10.46, 10.02.

2,2',2'',2'''-((2*S*,5*S*,8*S*,11*S*)-2,5,8,11-tetraisobutyl-1,4,7,10-tetraazacyclododecane-1,4,7,10-tetrayl)tetraacetic acid (L3). The solution of **4c** (167 mg) in dried dichloromethane (2 ml) and trifluoroacetic acid (2 ml) was stirred at room temperature for 16 hours, the solvents were concentrated and then added 2 ml of 1 M hydrochloride acid, the acidic solution was concentrated under vacuum and the residue was dissolved in 5 ml of water, the solution was lyophilized, this resulted in the product as an off-white powder (140 mg, yield 96.4%), it was used directly in the next step without further purification. ^{1}H NMR (600 MHz, D_2O) δ 4.08 (t, *J* = 15.9 Hz, 2H), 3.84 (t, *J* = 20.6 Hz, 2H), 3.73 (dd, *J* = 22.4, 15.0 Hz, 4H), 3.58 (d, *J* = 11.6 Hz, 2H), 3.18 (t, *J* = 13.8 Hz, 4H), 3.10 (d, *J* = 16.3 Hz, 2H), 2.94 – 2.76 (m, 4H), 1.60 (m, 6H), 1.46 (dd, *J* = 31.9, 19.8 Hz, 4H), 1.41 – 1.29 (m, 2H), 1.00 – 0.80 (m, 24H); ^{13}C NMR (150 MHz, D_2O) δ 173.73, 168.28, 59.36, 52.32, 51.55, 49.67, 49.43, 48.06, 33.34, 32.28, 24.05, 22.59, 22.26, 20.29, 20.08.

2,2',2'',2'''-((2*S*,5*S*,8*S*,11*S*)-2,5,8,11-tetrabenzyl-1,4,7,10-tetraazacyclododecane-1,4,7,10-tetrayl)tetraacetic acid (L4). The solution of **4d** (250 mg) in pure trifluoroacetic acid (3 ml) was stirred at room temperature for 16 hours, the solvents were concentrated and then added 5 ml of 1 M hydrochloride acid, the acidic solution was concentrated under vacuum and the residue was dissolved in 10 ml of water, the solution was lyophilized, this resulted in a light yellow solid (220 mg, yield 95.6%), it was used directly to the next step without any further purification. ^{1}H NMR (600 MHz, CD_3OD) δ 7.61 (t, *J* = 7.4 Hz, 2H), 7.44 (t, *J* = 7.4 Hz, 4H), 7.35 – 7.15 (m, 6H), 6.53 (d, *J* = 7.4 Hz, 4H), 6.44 – 6.34 (m, 4H), 4.44 (d, *J* = 16.6 Hz, 2H), 4.23 – 4.03 (m, 6H),

3.50 (t, J = 15.3 Hz, 2H), 3.32 – 3.17 (m, 6H), 2.96 (d, J = 12.5 Hz, 2H), 2.89 (t, J = 10.8 Hz, 2H), 2.81 (d, J = 13.3 Hz, 2H), 2.51 (ddd, J = 29.5, 23.4, 11.6 Hz, 6H); ^{13}C NMR (150 MHz, MeOD) δ 172.79, 166.50, 136.72, 135.13, 129.22, 128.55, 128.51, 128.20, 127.32, 126.49, 63.44, 58.26, 50.18, 49.13, 48.00, 30.65, 29.87.

Synthesis of 2,2',2'',2'''-((2*S*,5*S*,8*S*,11*S*)-2,5,8,11-tetrakis(4-aminobutyl)-1,4,7,10-tetraazacyclododecane-1,4,7,10-tetrayl)tetraacetic acid (L5). The solution of **4e** (150 mg, 0.094 mmol) in trifluoroacetic acid (2 ml) was stirring at room temperature for overnight. The mixture was concentrated to get the final ligand **L5** in the form of trifluoroacetic acid salt (150 mg, yield 100%). ^{1}H NMR (400 MHz, D_2O) δ 4.42 – 2.67 (m, 28H), 2.10 – 1.16 (m, 24H). ^{13}C NMR (100 MHz, D_2O) δ 174.32, 168.50, 163.35, 163.00, 162.65, 162.30, 120.75, 117.85, 114.95, 112.05, 61.13, 59.70, 52.52, 51.02, 50.27, 49.66, 49.05, 39.10, 27.09, 26.79, 24.79, 24.62, 23.33.

General method of synthesis [LnL1]$^-$ - [LnL5]$^-$. To a solution of the ligands of **L1** – **L5** (TFA salt or HCl salt) in water was added $LnCl_3\cdot 6H_2O$ (~ 1.05 eq.), then adjusted the pH value to 7.0 by adding 0.01 M NaOH, the mixture was stirred at 80 °C for 12 hours. Cooled to room temperature and the pH was adjusted to 10, filtered and the solution was adjusted pH to 7.0 again, concentrated under vacuum. The salt of NaCl or CF_3COONa in the complexes of **[GdL1]$^-$** and **[GdL2]$^-$** was removed by semi-preparative reverse phase HPLC, with the mobile phases of acetonitrile and the solution of ammonium formate or ammonium acetate (10 mM), higher than 90% yields were obtained. **[LnL2A]$^-$** and **[LnL2B]$^-$** as well as their enantiomers **of [Ln(*R*)L2A]$^-$** and **[Ln(*R*)L2B]$^-$** were isolated by HPLC with the same method. They were confirmed by elemental analysis and HR-MS. **[GdL1]$^-$**: HRMS (m/z): [M]$^-$ calcd. for $C_{20}H_{32}GdN_4O_8$, 614.1462; found 614.1466; CNH elemental analysis (calcd., found for $C_{20}H_{34}GdN_4NaO_9\cdot 5.55H_2O\cdot 0.95NH_4COO$): C (30.92, 30.95), H (6.06, 6.09), N (8.52, 8.55). **[GdL2A]$^-$**: HRMS (m/z): [M]$^-$ calcd. for $C_{24}H_{40}GdN_4O_8$, 670.2088; found 670.2095; CNH elemental analysis (calcd., found for $C_{24}H_{42}GdN_4NaO_9\cdot 5\ H2O\cdot 1.5\ NH_4COO$): C (34.26, 33.99), H (6.54, 6.85), N (8.62, 8.59). **[GdL2B]$^-$**: HRMS (m/z): [M]$^-$ calcd. for $C_{24}H_{40}GdN_4O_8$, 670.2088; found 670.2096; CNH elemental analysis (calcd., found for $C_{24}H_{42}GdN_4NaO_9\cdot 2.6\ H_2O\cdot 0.95\ NH_4COO$): C (36.70, 36.91), H (6.29, 6.52), N (8.49, 8.70). **[EuL2A]$^-$**: HRMS (m/z): [M]$^-$ calcd. for $C_{24}H_{40}EuN_4O_8$, 665.2059; found 665.2069; CNH elemental analysis (calcd., found for $C_{24}H_{42}EuN_4NaO_9\cdot 5\ H_2O\cdot 1.75\ NH_4COO$): C (34.20, 33.81), H (6.58, 6.77), N (8.91, 8.54). **[EuL2B]$^-$**: HRMS (m/z): [M]$^-$ calcd. for $C_{24}H_{40}EuN_4O_8$, 665.2059; found 665.2065; CNH elemental analysis (calcd., found for $C_{24}H_{42}EuN_4NaO_9\cdot 2.55\ H_2O\cdot 0.95\ NH_4COO$): C (36.98, 37.27), H (6.33, 6.62), N (8.55, 8.83). **[Gd(R)L2A]$^-$**: HRMS (m/z): [M]$^-$ calcd. for $C_{24}H_{40}GdN_4O_8$, 670.2088; found 670.2094. **[Gd(R)L2B]$^-$**: HRMS (m/z): [M]$^-$ calcd. for $C_{24}H_{40}GdN_4O_8$, 670.2088; found 670.2097. **[GdL3]$^-$**: HRMS (m/z): [M]$^-$ calcd. for C32H56GdN4O8, 782.3340; found 782.3429; CNH elemental analysis (calcd., found for $C_{32}H_{58}GdN_4NaO_9\cdot 6.05\ H_2O\cdot 6.4\ NaCl$): C (29.43, 29.41), H (5.41, 5.39), N (4.29, 4.31). **[GdL4]$^-$**: HRMS (m/z): [M]$^-$ calcd. for $C_{44}H_{48}GdN_4O_8$, 918.2714; found 918.2755; CNH elemental analysis (calcd., found for $C_{44}H_{50}GdN_4NaO_9\cdot 8.4\ H_2O\cdot 4.35\ NaCl$): C (38.73, 38.75), H (4.93, 4.95), N (4.11, 4.08). **[GdL5]$^-$**: HRMS (m/z): [M+3H]$^{2+}$/2 calcd. for 1/2[$C_{32}H_{63}GdN_8O_8$], 422.7003; found 422.6960.

General procedure for the isomerism study of [LnL1]$^-$ - [LnL5]$^-$: Into an NMR test tube was added ~ 5 mg of ligand, dissolved in deuterium oxide (0.45 ml) or DMSO-d^6/D_2O (1:1) (0.45 ml), then added 1.05 equivalents of $EuCl_3\cdot 6H_2O$ or $YbCl_3\cdot 6H_2O$, the solution was adjusted pH to ~ 7.0 by diluted sodium deuteroxide in deuterium oxide solution. Then the mixture was heated at 60 ~ 80 °C for about 5 mins, which was cooled to room temperature and performed the measurements directly. Samples were performed these measurements after several days to months later again, there was no obvious change. **[EuL1]$^-$**: HRMS (m/z): [M]$^-$ calcd. for C20H32EuN4O8, 609.1433; found 609.1443. **[EuL2]$^-$**: HRMS (m/z): [M+2H]$^+$ calcd. for $C_{24}H_{42}EuN_4O_8$, 667.2216; found 667.2207. **[EuL3]$^-$**: HRMS (m/z): [M]$^-$ calcd. for $C_{32}H_{56}EuN_4O_8$, 777.3311; found 777.3300. **[EuL4]$^-$**: HRMS (m/z): [M+2H]$^+$ calcd. for $C_{44}H_{50}EuN_4O_8$, 915.2842; found 915.2859. **[EuL5]$^-$**: HRMS (m/z): [M+3H]$^{2+}$/2 calcd. for 1/2[$C_{32}H_{63}EuN_8O_8$], 420.1987; found 420.1983. [YbDOTA]$^-$: HRMS (m/z): [M]$^-$ calcd. for $C_{16}H_{24}N_4O_8Yb$, 574.0983; found 574.0989. **[YbL1]$^-$**: HRMS (m/z): [M+2H]$^+$ calcd. for $C_{20}H_{34}N_4O_8Yb$, 632.1766; found 632.1754. **[YbL2]$^-$**: HRMS (m/z): [M+2H]$^+$ calcd. for $C_{24}H_{42}N_4O_8Yb$, 688.2392; found 688.2392. **[YbL3]$^-$**: HRMS (m/z): [M+2H]$^+$ calcd. for $C_{32}H_{58}N_4O_8Yb$, 800.3644; found 800.3643. **[YbL4]$^-$**: HRMS (m/z): [M+2H]$^+$ calcd. for $C_{44}H_{50}N_4O_8Yb$, 936.3018; found 936.3020. **[YbL5]$^-$**: HRMS (m/z): [M+3H]$^{2+}$/2 calcd. for 1/2[$C_{32}H_{63}YbN_8O_8$], 430.7076; found 430.7072.

General method of synthesis CuDOTA and **CuL2.** Into a flask was added ligand (20 mg), water (2 ml) and $CuCl_2$ (1.05 eq.), then the reaction mixture was adjusted pH to ~ 7.0 by diluted sodium oxide solution. After stirring at room temperature of 1 h, two isomers were isolated by semi-preparative reversed-phase HPLC, with the mobile phases of acetonitrile and the solution of ammonium formate (10 mM). The fractions were dried through lyophilization. CuDOTA: HRMS (m/z): $[M+Na]^+$ calcd. for $C_{16}H_{26}CuN_4O_8Na$, 488.0945; found 488.0930. **CuL2**: HRMS (m/z): $[M+H]^+$ calcd. for $C_{24}H_{43}CuN_4O_8$, 578.2377; found 578.2416.

Crystallographic data. A suitable crystal was picked and mounted using the "oil-drop mounting" technique, and its data was collected using either the Bruker Smart Apex II or Bruker D8-Venture single crystal diffractometer. Multi-scan absorption correction was then applied to the data using the Bruker SAINT/SADABS software[5]. The SHELX program suite[6] was used to calculate the initial structural solution through either direct or Patterson method, which would then be refined by full matrix least-squares on F2.

RP-HPLC conditions for the stability study: Waters UPLC system with fluorescence detection (λ_{exc} = 274 nm, λ_{em} = 311 nm)[7,8]. The mobile phase A was water with 10 mM ammonium formate added; mobile phase B was 90% acetonitrile / 10% water with 10 mM ammonium formate added; Condition A: Using a Waters XBridge Shield RP18 2.1 µm, 50 × 2.1 mm, flow rate 0.2 ml/min, from 90% A / 10% B, the fraction of B increased to 90% over 8 min, then maintained at this fraction for 3 min, the fraction of B was then turned back to 10% within 1 min and re-equilibrated at 10% B for 3 min. Condition B: Using a Waters XBridge HILIC 5 µm, 250 × 4.6 mm, flow rate 0.5 ml/min, from 95% A / 5% B, the fraction of B increased to 30% over 10 min, then maintained at this fraction for 5 min, the fraction of B was then turned back to 5% within 3 min and re-equilibrated at 5% B for 5 min.

Radio-HPLC conditions: Radio-HPLC was performed using an Agilent 1260 Infinity Series apparatus with Phenomenex Luna 5 u C18(2) 100 Å (150 × 4.6 mm); eluent A: water with 0.1% trifluoroacetic acid, eluent B: acetonitrile with 0.1% trifluoroacetic acid. The HPLC method used for is as follows. For **^{64}CuL2**: starting from 95% A for 2 min, the fraction of B was increased to 50% over 10 min. The column was washed with 95% B for 2 min and then ramped to 5% B to re-equilibrate the system; Rapid HPLC method for **^{64}CuL2** or **$[^{177}LuL2]^-$**: starting from 80% A for 1.5 min, the fraction of B was increased to 40% in 0.5 min and hold at 40% for 4 min; For DOTA: isocratic 100% A for 6 min.

Elemental analysis for the complexes: The elemental analysis (CNH) of the complexes of the complexes were performed under the Elementar CHNS elemental analyser and the solvent/salt contents were calculated by the online system of JASPER v2.0 - JavaScript Percentage Elemental Results Calculator (http://www.chem.yorku.ca/profs/potvin/Jasper/jasper2.htm).

Supplementary References

1. Gale, E. M., Kenton, N. & Caravan, P. [Gd(CyPic3A)(H2O)2]−: a stable, bis (aquated) and high-relaxivity Gd (iii) complex. *Chem. Commun.* **49**, 8060-8062 (2013).

2. Ranganathan, R. S. *et al.* Polymethylated DOTA Ligands. 1. Synthesis of Rigidified Ligands and Studies on the Effects of Alkyl Substitution on Acid− Base Properties and Conformational Mobility. *Inorg. Chem.* **41**, 6846-6855 (2002).

3. Raymond, K. N.; Corneillie, T. M.; and Xu, J. Luminescent hydroxyisophthalamide macrocyclic lanthanide complexes and derivatives. U.S. Patent 2007-US76047 2008063721, 20070815 (2008).

4. Tsuboyama, S., Tsuboyama, K., Higashi, I. & Yanagita, M. Cyclic tetramers of optically active aziridines: 1, 4, 7, 10-tetrabenzyl-2, 5, 8, 11-tetra-(R)-ethyl-1, 4, 7, 10-tetraazacyclododecane. *Tetrahedron Lett*. **11**, 1367-1370 (1970).

5. Krause, L., Herbst-Irmer, R., Sheldrick, G. M. & Stalke, D. Comparison of silver and molybdenum microfocus X-ray sources for single-crystal structure determination. *J. Appl. Cryst.* **48**, 3-10 (2015).

6. Sheldrick, G. M. A short history of SHELX. *Acta Cryst.* **64**, 112-122 (2008).

7. Hagan, J. J., Taylor, S. C. & Tweedle, M. F. Fluorescence detection of gadolinium chelates separated by reversed-phase high-performance liquid chromatography. *Anal. Chem.* **60**, 514-516 (1988).

8. Kumar, K., Sukumaran, K. & Tweedle, M. Determination of free gadolinium (3+) as a cyclohexanediaminetetraacetic acid complex by reversed-phase HPLC in ionic gadolinium (III) chelates. *Anal. Chem.* **66**, 295-299 (1994).
